# The Nature of Cell‐Penetrating Poly(disulfide)s

**DOI:** 10.1002/anie.7902673

**Published:** 2026-09-26

**Authors:** Saidbakhrom Saidjalolov, Giacomo Renno, Yuheng Zhang, Michael Cognet, Justin Neuman, Andreas Hennig, Naomi Sakai, Stefan Matile

**Affiliations:** ^1^ Department of Organic Chemistry University of Geneva Geneva Switzerland; ^2^ National Centre of Competence in Research (NCCR) Molecular Systems Engineering Basel Switzerland; ^3^ Center for Cellular Nanoanalytics (CellNanOs) and School of Biology/Chemistry Universität Osnabrück Osnabrück Germany

**Keywords:** cellular uptake, condensates, drug delivery, membrane proteins, membrane reorganization, proteomics, scramblases, tetraspanins, thiol‐mediated uptake

## Abstract

Cell‐penetrating poly(disulfide)s (CPDs) are the most popular thiol‐mediated uptake (TMU) tools for delivering attached substrates of interest (SOIs) into the cytosol. Despite their widespread use, their mode of action remains unknown. Herein, we elucidate how CPDs work using combined data from protein knockdown, µMap proteomics, coacervate assembly, mechanosensitive fluorescent membrane probes, CPD inhibitors, activators, and depolymerization. Their guanidinium sidechains first ion‐pair with anionic clusters on cell surfaces. By enhancing effective CPD concentration, these non‐covalent interactions enable, accelerate, and direct dynamic covalent disulfide exchange cascades with cellular thiols and disulfides. CPD bioconjugation rates match their uptake activity, exceeding those of disulfide monomers by 3000 times. Exchange with proteins capable of membrane remodeling allows CPDs to exploit these proteins' primary functions to mobilize anionic lipids (scramblases, ANO6), form toroidal elastic pores and related membrane deformations, and repair all after CPD‐SOI conjugates have passed through (tetraspanins, TSPAN8, flippases, etc.). Within cells, CPDs form coacervates and integrate into biological condensates, particularly nucleoli, until they depolymerize and release the SOIs. This understanding of the nature of CPDs also clarifies TMU in general and will guide future efforts to control cellular entry for drug delivery and pathogen defense.

## Graphical abstract

The mode of action of cell‐penetrating poly(disulfide)s (CPDs) is elucidated to understand thiol‐mediated uptake (TMU) in general and guide future efforts to control cellular entry for chemicals delivery and pathogen defense.

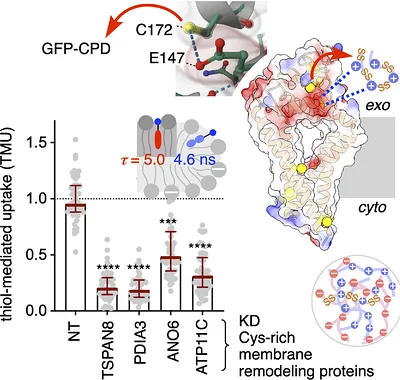

## 1 Introduction

Cell‐penetrating poly(disulfide)s (CPDs) were originally conceived as biodegradable, and therefore less toxic analogs of arginine‐rich cell‐penetrating peptides (CPPs) [1, 2]. Since then, they have proven highly effective for transporting nearly any substrate of interest (SOI) into the cytosol of not only cultured cells but also in live animals, as well as a wide variety of organs, tissues, organelles, and microorganisms [3, 4, 5, 6, 7]. CPDs, such as **1** or **2**, can be prepared by ring‐opening polymerization (ROP) of strained disulfides **3** [8, 9] using thiol‐containing SOIs **4** or **5** as initiators and iodoacetamide **6** as a terminator (Figure 1a). While the regioselectivity of ROP is poor under standard conditions, it can be controlled using, for example, organocatalysis [10, 11]. All components are variable, allowing modification of physical or biological properties to meet users’ needs [7]. The main drawback of this approach was the high SOI concentrations (>30 µM) required to initiate polymerization, which is impractical for precious SOIs. However, this issue was resolved last year with a new “grafting‐to” strategy, which enables the formation of conjugates such as **7** through dynamic covalent thiol‐disulfide exchange between preformed CPDs and thiolated SOIs such as GFP **8** at low concentrations (<50 nM) [12]. This represents an important step toward the broader use of CPDs for delivering biologically important SOIs into the cytosol.

**FIGURE 1:**
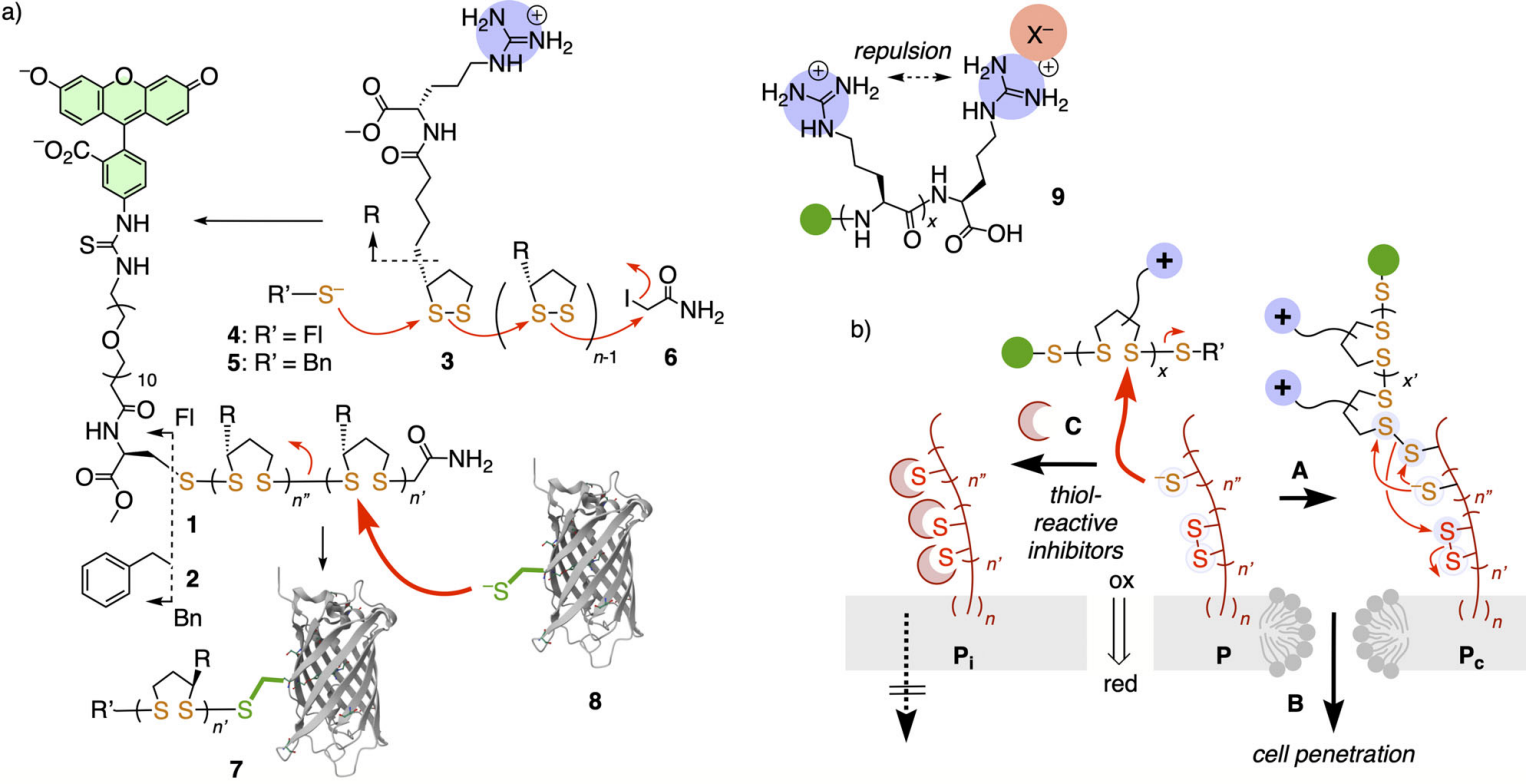
Synthesis and structure of CPD‐SOI conjugates (**1**, **7**), the general concept of TMU (b) and the general structure of arginine‐rich CPP‐SOI conjugates (**9**). (a) Ring‐opening polymerization (ROP) of monomers **3,** initiated by fluorescent and silent thiolates **4** and **5**, respectively, and terminated by **6** to afford Fl‐CPD_n_ **1** and Bn‐CPD_n_ **2**. CPDs **2** can be grafted to SOIs (e.g., S147C GFP **8**) to generate CPD‐SOI conjugates like **7**. CPDs are regio‐ and stereo‐irregular. *n*: number of monomers per polymer, estimated from number‐average molecular weight (dispersity = 1.1∼1.5) from size‐exclusion chromatography analyses (Figure S4 and Table S1). (b) CPDs are proposed to penetrate cells by (A) dynamic covalent disulfide exchange with cell surface proteins (P) to give CPD‐protein conjugates (P_c_), followed by exchange cascades within the same or among different proteins until reaching (B) toroidal elastics to translocate across the plasma membrane along the cellular redox gradient. (C) TMU can be inhibited by blocking cell‐surface thiols/disulfides with thiol‐reactive agents.

Despite this significant impact, the mode of action of CPDs remains unknown. We and many others found that uptake of CPDs is insensitive to endocytosis inhibitors but sensitive to thiol‐reactive agents [2, 3, 4, 5, 7], consistent with the mechanism of thiol‐mediated uptake (TMU) rather than endocytic pathways. TMU proceeds through a dynamic covalent exchange cascade between cellular thiols and multiple disulfides in the CPD backbone to penetrate cells (Figure 1bA). The general mechanism of TMU has been proposed early on based on eclectic findings, including similarities to certain viral entry mechanisms, but with relatively little follow‐up [13, 14, 15, 16, 17].

Direct translocation of giant substrates, from proteins to polymersomes, across the plasma membrane (PM) is only conceivable through toroidal elastics (or toroidal, micellar pores, Figure 1bB) [18]. Similar membrane structures have been proposed to account for the non‐endocytic uptake of CPPs [19, 20, 21]. As predecessors of CPDs, arginine‐rich CPPs such as HIV Tat peptide and oligoarginines (**9**; most commonly *x* = 7 or 8) are much better understood mechanistically [19, 20, 21, 22, 23, 24, 25, 26, 27, 28]. Central to their function is stable yet labile anion binding to guanidinium cations, which minimizes charge repulsion with nearby cations [29], thereby allowing CPPs to adapt to their environment through successive counteranion exchange. The presence of guanidinium groups in CPDs suggests that anion exchange also plays an important role in their function. However, CPDs additionally use TMU, which relies on dynamic covalent exchange chemistry with proteins along the cellular redox gradient. This feature appears advantageous for minimizing toxicity from prolonged membrane disturbance while benefiting from the selectivity inherent in protein chemistry.

Compared to CPDs, related small‐molecule TMU motifs, or cascade exchangers (CAXs), are also better understood mechanistically [30, 31]. CAXs, including CPDs, are dynamic covalent motifs that can undergo multiple exchanges with cellular thiols or disulfides to transport attached SOIs into cells (Figure 1b). The best‐performing small‐molecule CAXs include asparagusic acid (AspA, **10**) [32], epidithiodiketopiperazines (ETP, **11**) [33], Michael acceptors (MAC, **12**) [34, 35], and benzopolysulfanes (BPS, **13**) [36], most of which have been used to successfully deliver conjugated proteins into cells (Figure 2d) [34, 36, 37, 38, 39, 40]. Using patterns generated by inhibitor screening and proximity‐labeling µMap proteomics analysis, we identified orthogonal exchange cascades that account for the uptake of fluorescein‐labeled CAXs [30, 31]. Herein, we aim to elucidate the mode of action of CPDs using these established methodologies.

**FIGURE 2:**
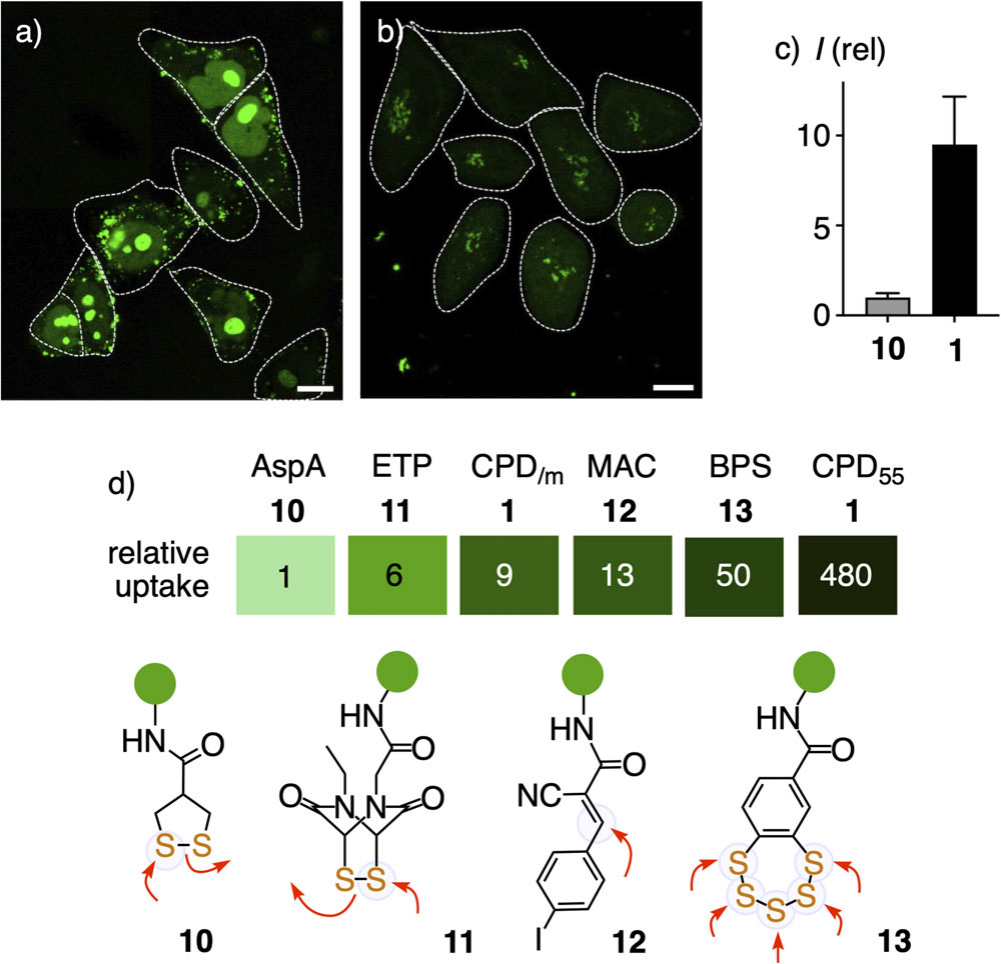
The absolute activity of Fl‐CPDs. (a,b) CLSM images and (c) relative fluorescence intensity of Fl‐CPD_55_ **1** (0.2 µM; (a,c) and fluorescein‐labeled AspA **10** (10 µM, b, c) within HK cells (30 min incubation, L15); mean ± SD (*n* = 10). (d) Relative uptake activity of Fl‐CPD_55_ **1** per polymer and per monomer (Fl‐CPD_/m_) compared to known small‐molecule fluorescein‐CAXs **10–13**. Their relative uptake activities are from reference [30], scale bars = 5 µm.

## 2 Results and Discussion

### 2.1 The Absolute Activity of CPDs

The popularity of CPDs [7] mainly stems from their efficiency at transporting large substrates, such as proteins, into cells. We noted that the cellular uptake of CPD‐protein conjugates is detectable at only 20 nM [12], whereas conjugates of proteins with small‐molecule CAXs typically require 10 µM [34, 36, 37, 38, 39, 40]. For comparison, CPPs usually also require a few µM for non‐endosomal uptake [22, 41].

To quantify the superior performance of CPDs compared to CAX monomers, intracellular fluorescence intensity from Fl‐CPD_55_ **1** was directly compared with Golgi‐tracking fluorescein‐labeled AspA **10** [32, 42] using confocal laser scanning microscopy (CLSM). CLSM imaging was essential for accurate quantification of SOIs within cells, as it allowed us to exclude contributions from PM‐bound species. HeLa Kyoto (HK) cells treated with a 50‐fold lower concentration of Fl‐CPD_55_ **1** exhibited 9.6‐times higher fluorescence, indicating that CPDs are 480‐times more active than AspA (Figure 2). Further comparison was made with other small‐molecule CAXs using previously determined relative uptake activities [30]. CPDs were far more efficient than the best small‐molecule CAX, BPS **13**, an intriguing bioinspired motif that dynamically exchanges into oligosulfide oligomers during TMU and is interesting but demanding to synthesize (Figure 2) [36].

The activity per disulfide monomer in Fl‐CPD_55_ **1** remained higher than that of ETP **11**, which is another intriguing bioinspired motif with high activity due to high ring tension (Figure 2) [33]. AspA was 6.4‐times more active than relaxed disulfide monomers in neutralized Cys dimers [32]. The same structural motif in Fl‐CPD_55_ **1** was thus ≥3000 times more active than as a monomer. A corresponding 3000‐fold rate enhancement was found for grafting‐to bioconjugation of CPDs compared to monomeric disulfides in cystine [12]. This similarity suggests that the acceleration of thiol/disulfide exchange with CPDs compared to disulfide monomers accounts for the high absolute TMU activity. Originating from secondary interactions dominated by repulsion‐driven ion pairing between sidechain guanidiniums and anion clusters on proteins, this privileged dynamic covalent exchange with both SOIs and cellular partners thus emerges as primary characteristic defining the nature of CPDs.

As the activity of CPDs depends on the number of monomer units per polymer [43], even higher activity may be achieved with longer polymers, but at the expense of increased toxicity. It is important to note that, because of the dynamic covalent chemistry involved, the activity of CPDs varies strongly with conditions, including the medium. Applications of CPDs in practice thus require meticulous adherence to reported protocols and insightful attention to modifications made.

### 2.2 CPDs Within Cells: Biomolecular Condensates

After cellular uptake, CPDs with non‐targeting SOIs usually accumulate in nucleoli, as do arginine‐rich CPPs (Figures 2a and 3a) [44]. This behavior is consistent with the high stability of multivalent ionic interactions between cationic CPD/CPP poly‐/oligomers and polyanionic nucleic acids in higher‐order condensates, which limits further anion exchange.

**FIGURE 3:**
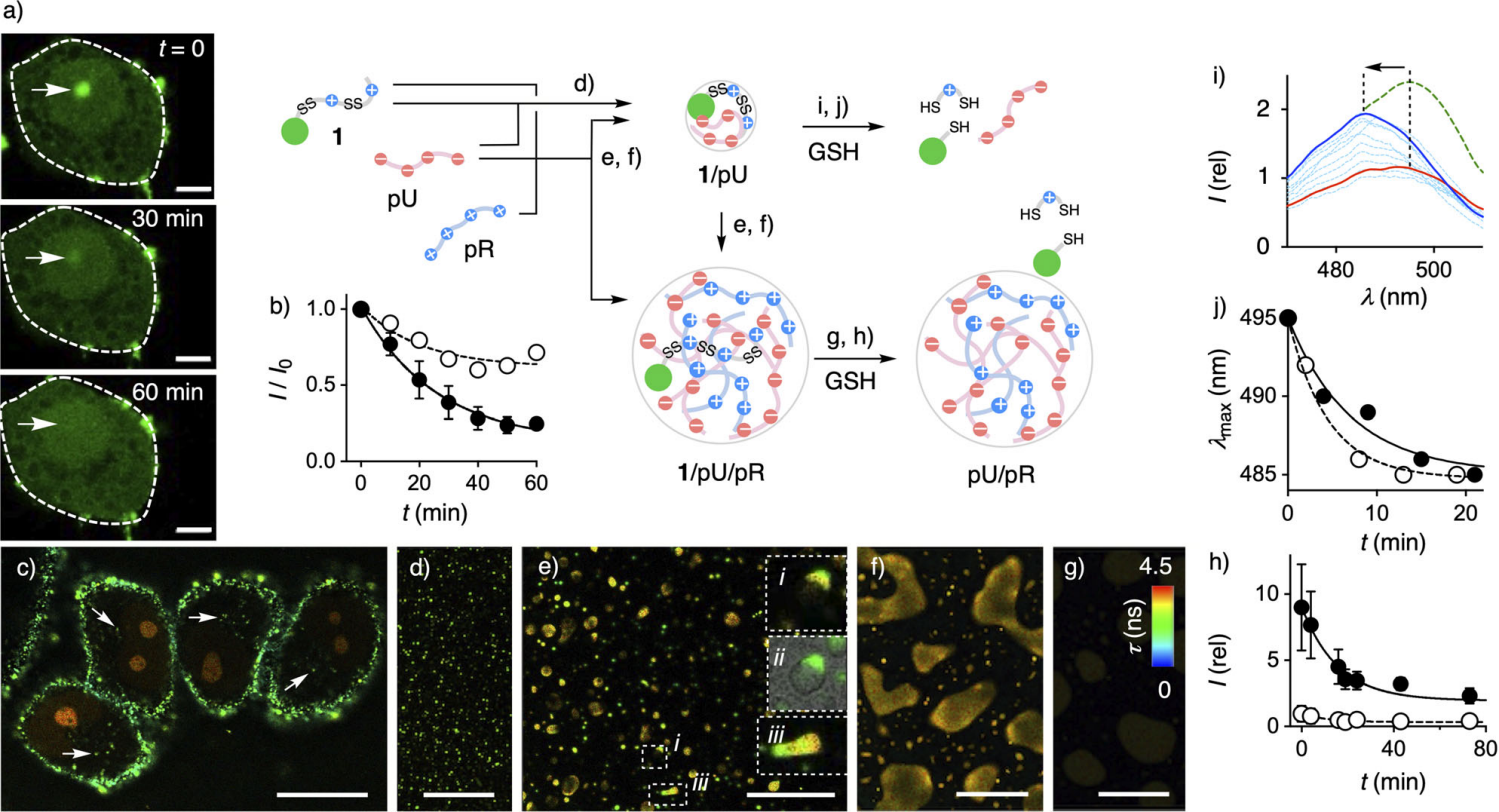
Assembly of CPDs in biomolecular condensates and release of SOIs by CPD depolymerization. (a) CLSM images of an HK cell 0, 30, and 60 min (top to bottom) after incubation with Fl‐CPD_34_ **1** (75 nM, 20 min, L15) and rinsing with heparin‐containing media. (b) Fluorescence intensity of nucleoli (filled circles) and cytosol (open circles) of HK cells incubated with Fl‐CPD_34_ **1** (75 nM, L15, 20 min) as a function of time relative to *I*_0_ at *t*_0_; mean ± SD (*n* = 3). (c–f) FLIM images of Fl‐CPD_34_ **1** with (c) HK cells, (d) pU (**1**/pU), and (e,f) pR/pU (**1**/pU/pR) 10 min (e) and 1 h (f) after mixing, with schematic interpretation (coacervate assembly, ripening). Insets in (e) are zooms of the indicated areas (*i*, *iii*) compared to a merged image of fluorescence (green) and bright field (gray, *ii*). (g) FLIM image of **1**/pU/pR 45 min after GSH addition as an example for (h) fluorescence intensity within (filled) and outside coacervates (open circles) with time after GSH addition, with schematic interpretation (CPD depolymerization, SOI release). (i) Excitation spectra of **1**/pU in PBS with time after GSH addition (blue; green: **1**, red: **1**/pU, *λ*_em_ = 520 nm) as an example for (j) maxima of **1**/pU (filled circles, i) compared to **1** (open circles, Figure S17) in PBS with time after GSH addition, with schematic interpretation (CPD depolymerization, coacervate disassembly). Equal brightness and contrast for (f) and (g); scale bars: (a) 5 µm, (c) 20 µm, and (d–g) 50 µm, kinetics fit to exponential decay, mean ± SD (*n* = 8).

Because the fluorescence lifetime of fluorescein decreases with increasing effective local concentrations from *τ* = 3.7 ns to *τ* = 1.4 ns [45], fluorescence lifetime imaging microscopy (FLIM) was used to elaborate on liquid‐liquid phase separation (LLPS) with CPDs. All FLIM images were analyzed using an assumption‐free phasor method to extract fluorescence lifetimes (Figure S19) [46]. FLIM images of HK cells treated with Fl‐CPDs **1** showed long fluorescence lifetimes for the nucleoli (*τ* = 3.7∼4.1 ns) and short fluorescence lifetimes for the PM and puncta in the cytosolic region (arrows, *τ* = 2∼3 ns; Figure 3c). High local concentrations of Fl‐CPDs **1** at the PM were consistent with CPD binding to cell‐surface anions, as reported for arginine‐rich CPPs [23, 47, 48].

Model coacervates composed of polyuridine (pU) and polyarginine (pR) were used as mimics of nucleoli, which are representative biomolecular condensates [49, 50]. Biomolecular condensates are membraneless cellular compartments with diverse functions, generally composed of RNAs and proteins with intrinsically disordered, positively charged domains [49]. Similar structures can be formed in vitro, by mixing polycations and polyanions, resulting in LLPS of the complexes into dense droplets that sequester various substrates [51, 52, 53].

Large condensates with long (*τ* = 3.6 ns) and small puncta with short fluorescence lifetime (*τ* ≈ 2.5 ns) were generated by mixing Fl‐CPD **1** with pU and pR in PBS (Figure 3e). Small puncta with short lifetime were also obtained by mixing **1** and pU without pR (*τ* = 2.4 ns, Figure 3d). Their similarity implied that the small puncta found in the presence of pR also originate from condensates **1**/pU assembled from Fl‐CPD **1** and pU only (Figure 3e). After a longer incubation period, larger structures grew by Ostwald ripening, while puncta with short lifetimes became invisible (Figure 3f). Coalescence events could be detected between punctate and larger unstained structures (Figure 3e, inset, *ii*), accompanied by a gradual increase in lifetime (*i* and *iii*). These results suggested that small **1**/pU puncta merge with larger pR/pU droplets, diluting **1** locally within the droplets and thus increasing fluorescence lifetimes. Such coalescence events are generally considered as evidence for a liquid‐like nature [53].

The similarity of large condensates with long and small condensates with short fluorescence lifetime in cells and models implied that similar processes might occur in cells. The smaller puncta with high CPD concentrations (*τ* = 2.4 ns, Figure 3c, white arrows) conceivably originated from CPD/oligonucleotide condensates in the cytosol, while CPD integration into the larger nucleoli reduces local concentration and thus increases lifetimes (*τ* ≈ 4.0 ns). Puncta of varying dimensions in the cytosol have been regularly observed in microscopic images of CPD‐treated cells and shown by several groups not to belong to endo/lysosomes [3, 4, 7].

### 2.3 CPDs Within Cells: Dynamic‐Covalent Depolymerization

The nucleolar fluorescence of Fl‐CPD **1** in CLSM images dissipated over time (Figure 3a,b, filled circles, S20). Photobleaching was unlikely to account for this change because the cytosolic fluorescence decreased less over the same period (Figure 3b, open circles). The likely cause of this change therefore was dynamic covalent depolymerization of CPD dynamers by intracellular thiols, particularly glutathione (GSH), leading to the release of the fluorophore (Fl) by diminishing multivalent electrostatic interactions with oligonucleotides (Figure 3g,h). The released fluorophore lacks cellular targets and thus spreads throughout the cytosol and nucleus before being slowly exported from the cells.

In model coacervates, the fluorescence of objects integrating Fl‐CPD **1** diminished in the presence of GSH. In FLIM images, GSH addition to **1**/pU/pR droplets (Figure 3f) caused a gradual decrease in droplet fluorescence (Figure 3g,h). Such fluorescence silencing by GSH was consistent with Fl release from pU/pR coacervates, thus supporting that Fl release from nucleoli originates from CPD depolymerization (Figure 3a) [54, 55]. Similarly, the small puncta originating from **1**/pU coacervates disappeared after their treatment with GSH (Figures 3d and S18), consistent with the disassembly of **1**/pU coacervates upon depolymerization of Fl‐CPD **1** by dynamic covalent disulfide exchange (Figure 3i,j).

Since the excitation and emission maxima of fluorescein in **1** are red‐shifted compared to those of the monomer, likely due to differences in the local hydrogen‐bonding environment of the dye [56], the disassembly of **1**/pU coacervates could be monitored in the excitation spectra of the fluorescein probe. In the absence (green) and the presence of pU (red), the depolymerization of CPDs with GSH caused a blue shift (Figure 3i, blue, arrow). The depolymerization of CPD **1** in neutral buffer (Figure 3j, open circles, *t*_1/2_ ≈ 3 min) was only slightly faster than that in **1**/pU puncta in the presence of GSH (Figure 3j, filled circles, *t*_1/2_ ≈ 5 min). Similar kinetics were consistent with the liquid nature of the puncta, which allows free diffusion of GSH. This corroborative support for **1**/pU assemblies as coacervates was particularly important because their similarity with the puncta in the cytosol of CPD‐treated cells confirmed that after TMU, CPDs do not only integrate into biomolecular condensates in the nucleus (i.e., nucleoli), but also form (or integrate into) condensates in the cytosol.

### 2.4 CPDs and Cellular Membranes

The association of CPDs with the cell surface was expected to affect membrane organization for various reasons. For CPPs, temporary membrane reorganization has been detected with polarity‐sensitive dyes or tension‐sensitive flipper probes [57, 58, 59]. Flipper probes are twisted push‐pull fluorophores that planarize upon application of lateral force by ordered membranes, resulting in a red shift of the excitation maxima and an increase in fluorescence lifetimes (Figure 4) [60, 61]. Such lifetime sensitivity can be exploited for monitoring temporal and localized changes in membrane order and tension using FLIM [62].

**FIGURE 4:**
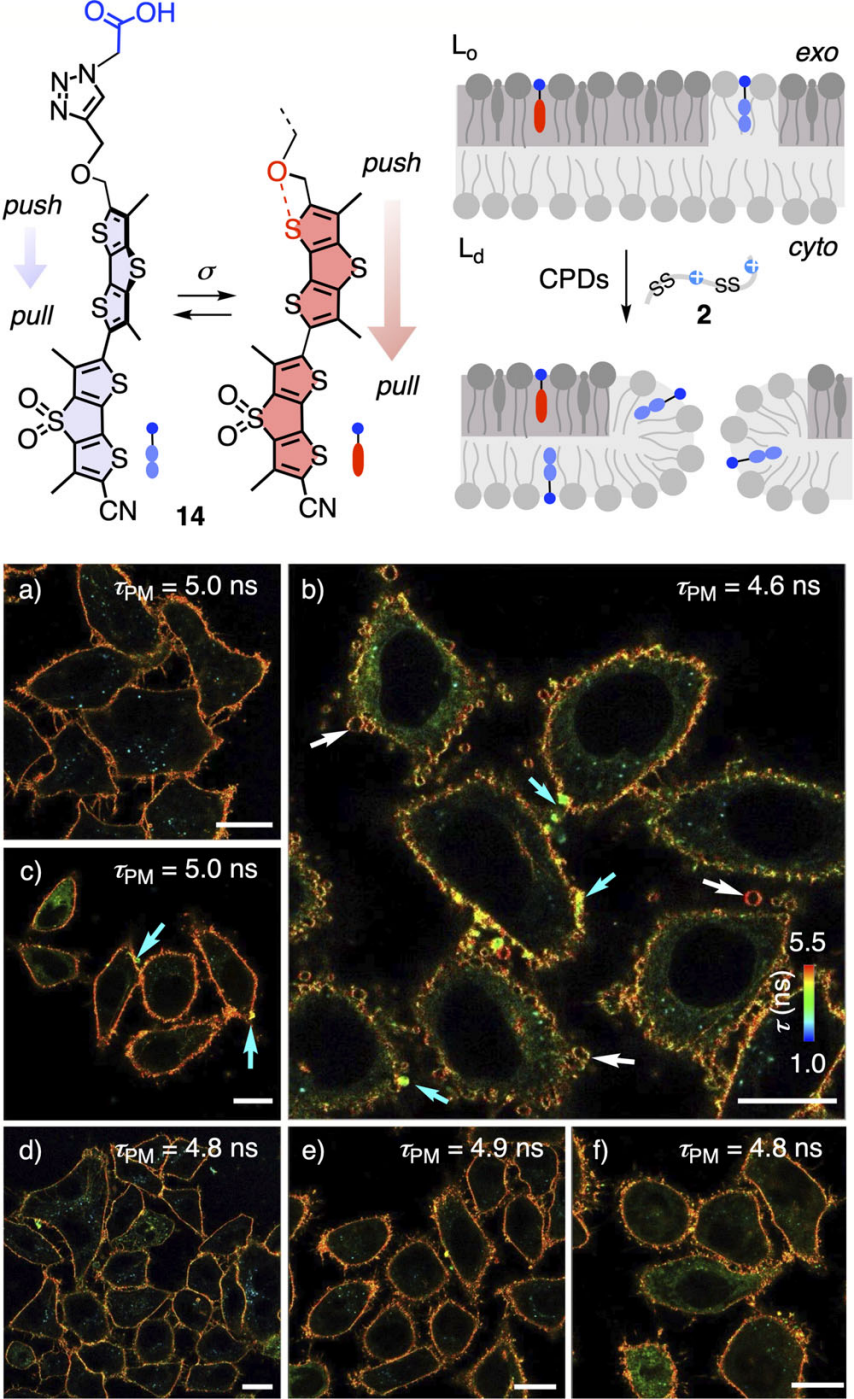
FLIM images of HK cells with flipper **14** (0.2 µM) (a) before and (b,c) after addition of (b) Bn‐CPD **2** (*c*_M_ ≈ 4 µM) and (c) pR (*c*_M_ ≈ 4 µM). (d) Flipper **14** was added after Bn‐CPD **2** treatment and heparin (0.1%) wash. (e) HK cells treated with dMAC (20 µM), then flipper **14**, (f) followed by Bn‐CPD **2**. *σ* = physical compression, *τ*_PM_ = fluorescence lifetime at plasma membranes, scale bars = 20 µm.

The lipid bilayers of the PM are highly asymmetrical with an ordered (L_o_) exoplasmic layer and a disordered (L_d_) cytoplasmic layer [63]. Since Flipper‐TR **14** do not flip‐flop [64], they selectively report the order of the exoplasmic layer with a high fluorescence lifetime in untreated cells. At the toroidal elastic pores proposed to contribute to TMU, the lipid packing should be loosened (Figures 1b and 4). At the same time, these transient micellar defects should allow flippers to flip into the less ordered cytoplasmic layer. Both events should shorten the fluorescence lifetime of the flipper probe.

The addition of nonfluorescent Bn‐CPD_38_ **2** (Figure 1) to HK cells stained with Flipper‐TR **14** (Figures 4a) resulted in a marked shortening of the overall fluorescence lifetime at the PM from *τ*_PM_ = 5.0 to 4.6 ns (Figures 4b and S22). These shortened lifetimes were as expected for membrane disorganization by CPDs. They were consistent with the *τ*_PM_ = 4.2 ns recorded previously with flippers covalently attached to AspA, the classical TMU tag introduced above (Figure 2) [42]. Domains with even lower lifetimes (3.7∼4.0 ns, cyan arrows) were detected, similar to those observed in the presence of polyarginine (pR, Figure 4c) and other CPP mimics [57]. Although electrostatic interactions with pR or CPDs might cause aggregation of Flipper **14**, resulting in shorter fluorescence lifetimes, such changes were barely detectable in control experiments with PM‐mimetic GUVs (Figure S21) [65]. Except for these domains, the order of the PM of cells treated with pR did not change (*τ*_PM_ = 5.0 ns, Figure 4c).

Under the selected conditions, some of the CPD‐treated cells could be seen surrounded by exovesicles with longer lifetimes (4.9∼5.3 ns, Figure 4b, white arrows). They were reminiscent of structures found during the formation of giant PM vesicles (GPMVs), and lipid scrambling has been reported to contribute to exovesiculation [66, 67, 68]. Although such changes in membrane order are potentially catastrophic, exoplasmic membrane order recovered quickly after CPD removal by rinsing with heparin‐containing medium (Figure S9) [2, 69], as evidenced by post‐staining with Flipper **14** (*τ*_PM_ = 4.7∼4.9 ns, Figure 4d). Exovesiculation has also been observed previously upon irradiation of flippers in the absence of CPDs [70]. However, incubation of cells with a known TMU inhibitor, dMAC [71], reduced the effects of CPDs on membrane order (*τ*_PM_ = 4.8 ns) and on exovesiculation (Figure 4e,f).

These results supported that dynamic covalent exchange between CPDs and cellular thiols can induce membrane reorganization, consistent with the proteins identified below. Overall reduced membrane order and the possible emergence of membrane deformations in the presence of CPDs were, of course, in excellent agreement with the formation of toroidal elastic pores that explain how TMU can transduce large substrates directly across the PM into the cytosol (Figures 1 and 4). The inability of pR as CPP mimic to cause similar membrane disorder and deformation supported that the nature of CPDs and arginine‐rich CPPs are not the same.

### 2.5 Inhibitors of Thiol‐Mediated Uptake

The differences between CPDs and arginine‐rich CPPs observed with flipper probes supported the significant contributions of TMU for CPD uptake, which are absent with disulfide‐free CPPs. Occurrence and significance of the dynamic covalent disulfide exchange networks accounting for TMU are routinely assessed by inhibition with thiol‐reactive agents (Figure 1bC). Ellman's reagent, DTNB **15**, has been the most widely used TMU inhibitor [7], while many more potent inhibitors have been developed with different selectivity profiles [30, 72, 73]. Notably, distinct selectivity patterns found for three representative CAX transporters, AspA **10**, ETP **11**, and BPS **13**, have been used to identify the existence of orthogonal exchange networks accounting for TMU [30] (Figure 5).

**FIGURE 5:**
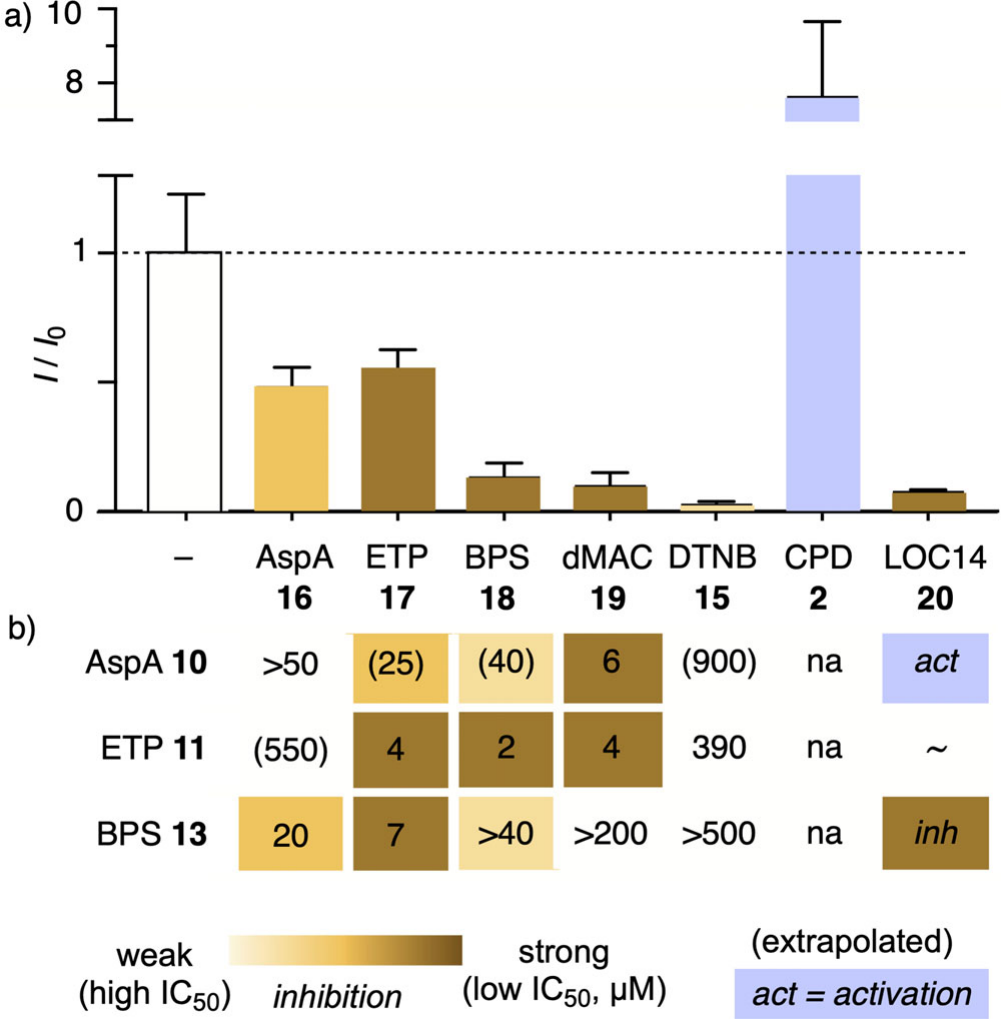
Response of CPDs to TMU inhibitors. (a) Intracellular fluorescence intensity *I/I*_0_ of Fl‐CPD_34_ **1** (75 nM) incubated without (–; *I* = *I*_0_) and with TMU inhibitors AspA (20 µM), ETP (5 µM), BPS (20 µM), dMAC (20 µM), DTNB (500 µM), Bn‐CPD_38_ **2** (75 nM), and LOC14 (20 µM; L15); mean ± SD (*n* = 3). (b) Focused heatmap for fluorescein‐labeled CAXs for comparison. Values correspond to IC_50_ under co‐incubation conditions, na = not applicable, data from ref [30]. For structures of inhibitors **15–20**, please see Figure S2.

The inhibition of CPD uptake has been measured for some TMU inhibitors to compare with these established selectivity patterns [12]. To validate and complement CPD inhibition studies, HK cells were treated for 1 h with TMU inhibitors **15–20** at concentrations near their IC_50_ for pertinent CAX transporters, then with Fl‐CPDs **1**, rinsed, and imaged using CLSM. Strongly reduced intracellular fluorescence was observed in the presence of most TMU inhibitors (Figures 5a and S11–S14). The selectivity profile was not identical to that of any of the CAXs but was most similar to BPS, especially in its sensitivity to the neutral PDI inhibitor LOC14 (**20**, Figure 5b) [30]. Based on this PDI inhibitor sensitivity, we previously designated PDIA3 as a primary protein for BPS uptake [30].

Bn‐CPD_38_ **2** was also tested as an inhibitor to evaluate potential self‐inhibition. However, the uptake of the fluorescent Fl‐CPD **1** increased rather than decreased in the presence of the nonfluorescent Bn‐CPD_38_ **2** (Figure 5a). This CPD self‐activation was probably due to an increased CPD concentration at the PM, which has often been found beneficial for CPP uptake [41, 47]. Dynamic covalent chain transfer [74], which increases polymer length [43], was considered as an appealing alternative explanation but has not yet been confirmed experimentally.

### 2.6 Activators of Cell‐Penetrating Peptides

Since arginine‐rich CPPs operate by proximity‐enhanced ion pairing with anionic lipids [29, 75], their cellular uptake can be improved by adding hydrophobic counteranions, such as pyrenebutyrate **21** [76], boron clusters **22** [77], amphiphilic calixarenes **23** [78], or fluorophile **24** [75]. However, the addition of these counteranions inhibited rather than activated the uptake of Fl‐CPD **1** and GFP‐CPD **7** into HK cells (Figures 6, S15, and S16).

**FIGURE 6:**
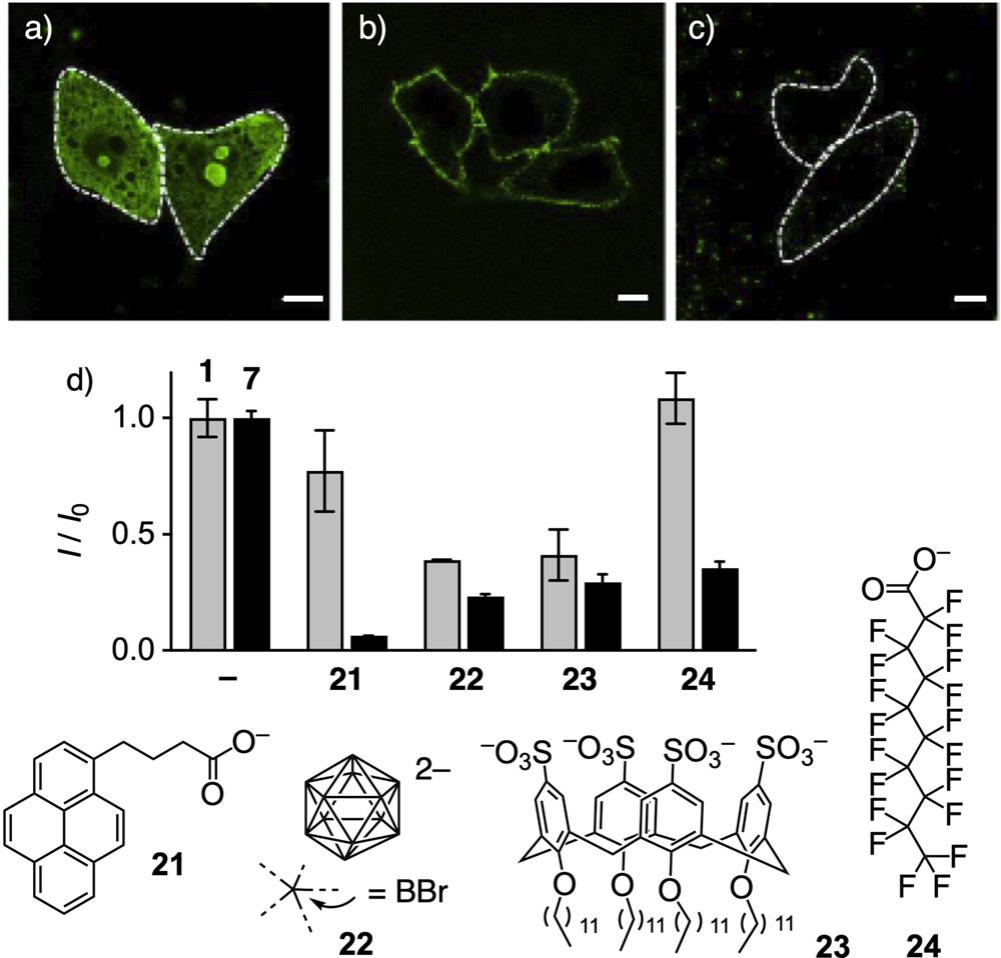
Response of CPDs to CPP activators. (a–c) CLSM images of HK cells incubated with GFP‐CPD **7** (75 nM, 20 min, L15) (a) without or with (b) **21** and (c) **22**. (d) Intracellular fluorescence intensity *I/I*_0_ of Fl‐CPD_34_ **1** (75 nM, gray) and GFP‐CPD **7** (75 nM, black) without (–; *I* = *I*_0_) and with **21** (50 µM), **22** (10 µM), **23** (10 µM), and **24** (10 µM); scale bars = 10 µm; mean ± SD (*n* = 3).

The failure of CPP activators to activate CPDs could originate from the longer polycations involved, which favor nonproductive self‐assembly into larger nanoparticles up to precipitation, possibly including PM binding but not translocation. The increase in binding to anionic membrane components with artificial anionic lipids is less important for CPDs because, first, they function primarily by disulfide exchange with proteins and, second, they can bring intrinsic anionic lipids to the cell surface by activating scramblases like ANO6 with dynamic covalent disulfide exchange (below). Interestingly, the involvement of scramblases has also been proposed in non‐endosomal CPP uptake, as increases in cytosolic Ca^2+^ concentration and externalization of phosphatidylserine precede uptake [79, 80]. Regardless of possible explanations, the inhibition of CPDs by CPP activators further supported that the modes of action of CPDs and arginine‐rich CPPs are not the same.

### 2.7 Microenvironment Proteomics of CPD Uptake

Photocatalytic microenvironment mapping (µMap) is a recently developed proximity‐labeling technology that enables selective labeling of proteins within close proximity (<4 nm) to the photocatalyst (PC) via Dexter energy transfer (DET) to diazirines (Figure 7) [81]. Applied to arginine‐rich CPPs [82, 83], µMap reliably identified proteins previously confirmed to participate in their uptake [20, 84, 85]. This method also proved useful in identifying numerous cysteine‐rich proteins, including several solute carriers (SLCs), flippases and TSPANs, with photocatalysts conjugated AspA and ETP [31]. The involvement of these proteins in TMU was confirmed by a strong reduction in the uptake of fluorescein‐labeled AspA **10** and ETP **11** following knockdown (KD) using the corresponding siRNAs [31].

**FIGURE 7:**
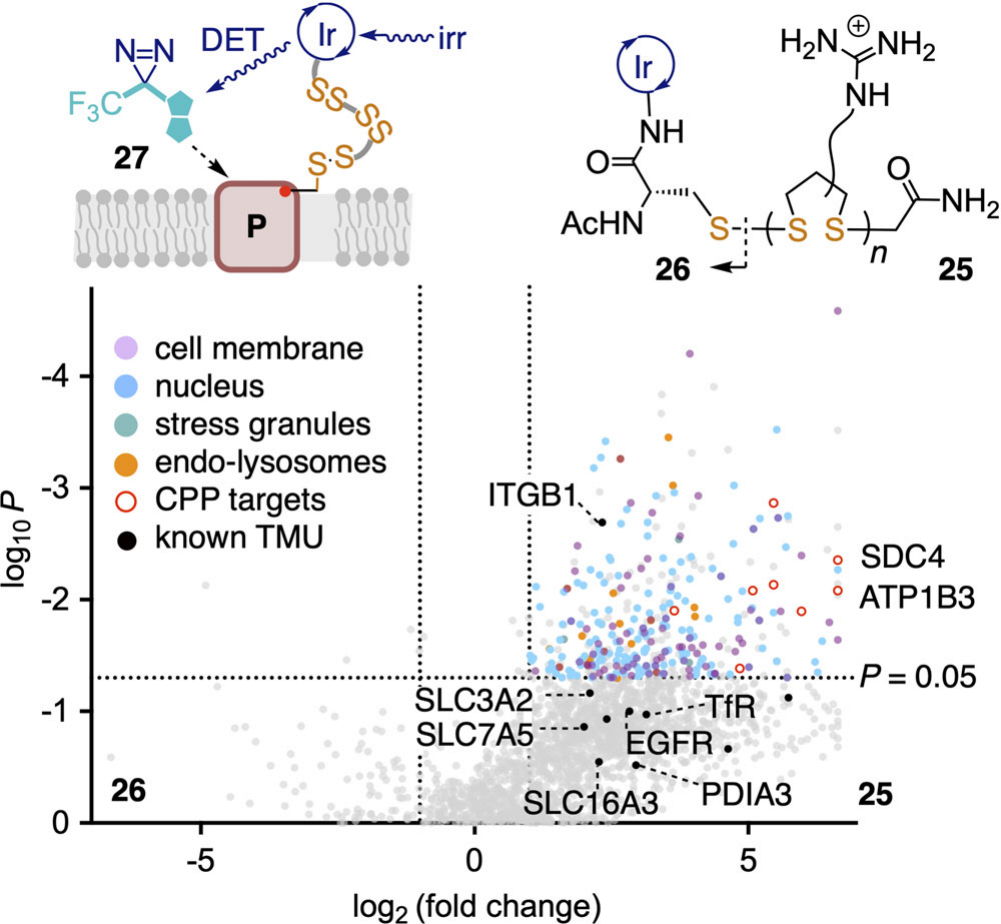
µMap photoproteomics for CPDs. Volcano plot comparing protein enrichments by PC‐CPD **25** (right) and PC‐Cys **26** (left) after incubation with HK cells (100 nM **25** or **26**, L15) under irradiation at 450 nm (125 µM **27**, 10 min), streptavidin pull‐down, digestion and MS analysis. Significantly enriched proteins, that is, log_2_ (fold change) ≥ 1, *p* ≤ 0.05 (366 proteins), located mostly in the nucleus (cyan, 168) and cell membranes (purple, 89), while those in endo/lysosomes (orange, 7) or stress granules and P‐body (green, 6) are rare. Also marked are known targets of arginine‐rich CPPs (red empty circles) and TMU (black, including *p* > 0.05).

To identify the proteins involved in CPD uptake, PC‐CPDs **25**, PC‐control **26**, and diazirine **27** were synthesized (Schemes S2, S4, and S5; Figures S3 and S4). HK cells were irradiated at 450 nm in the presence of diazirine **27** and either PC‐CPDs **25** or control PC‐Cys **26** (Figure 7). The resulting biotinylated proteins were pulled down, digested, and subjected to mass spectrometry‐based quantitative proteomics analysis. The volcano plot comparing protein enrichment by PC‐CPDs **25** (right) and PC‐Cys **26** (left) showed much more significantly enriched proteins on the PC‐CPD side (Figure 7). However, the enrichment was dominated by strongly interacting proteins, as indicated by their predominant nuclear localization (168/366 proteins, pale blue circles), and by the presence of the CPP targets (red circles), including proteoglycans, Syndecan‐4 (SDC4) and Glypican‐1 (GPC1), C‐X‐C chemokine receptor type 4 (CXCR4), Na^+^/K^+^‐transporting ATPase subunit b3 (ATP1B3), and metal cation symporter (SLC39A14) [20, 82, 83, 84, 85]. SDC4, in particular, has been considered as a primary protein for CPP uptake into endo/lysosomes, but was unlikely to be important for CPD uptake, which bypasses endo/lysosomes.

In contrast, none of the previously identified TMU proteins except for integrin β‐1 (ITGB1) was enriched significantly (*p* > 0.05). This may be because the control PC‐Cys **26** could also interact with TMU proteins. Compared to the less active small‐molecule CAXs AspA and ETP, of which µMap proteomics analysis was more informative [31], the higher activity of CPDs required the usable concentrations of PC to be much lower (Figure 2). It was thus likely that the concentrations of labeled partners during fast exchange were too low for detection. Overall, the µMap analysis did not provide much insight into the mechanism of CPD uptake. This elegant approach to track short‐lived intermediates seems more compatible with less active CAXs that operate at higher concentrations.

### 2.8 Knockdown of TMU Proteins

Although they were not prominent in µMap analysis with PC‐CPDs (Figure 7), previously identified proteins of TMU exchange networks of AspA and ETP were knocked down to assess their significance for the mode of action of CPDs (Figure 8). Added to these known proteins was a large neutral amino acid transporter heterodimer, SLC7A5/SLC3A2, which was found enriched in µMap analyses of PC‐BPS but showed no effect on BPS uptake upon KD (not shown). Also added was a calcium‐gated scramblase, ANO6 (aka TMEM16F), inspired by the inhibition of CPD uptake by extracellular or intracellular Ca^2+^ chelators, EDTA or BAPTA‐AM, respectively (Figures S28 and S29). Moreover, ANO6 was found to be essential for exovesiculation caused by thiol‐reactive agents during GPMV formation [67]. Similar exovesicles formed under certain conditions in the presence of CPDs and fluorescent flippers (Figure 4). All candidate proteins are cysteine‐rich cell‐surface proteins with overall anionic exofacial surfaces, thus capable of undergoing thiol/disulfide exchange reactions with CPDs, supported or even enabled by non‐covalent multiple ion pairing.

**FIGURE 8:**
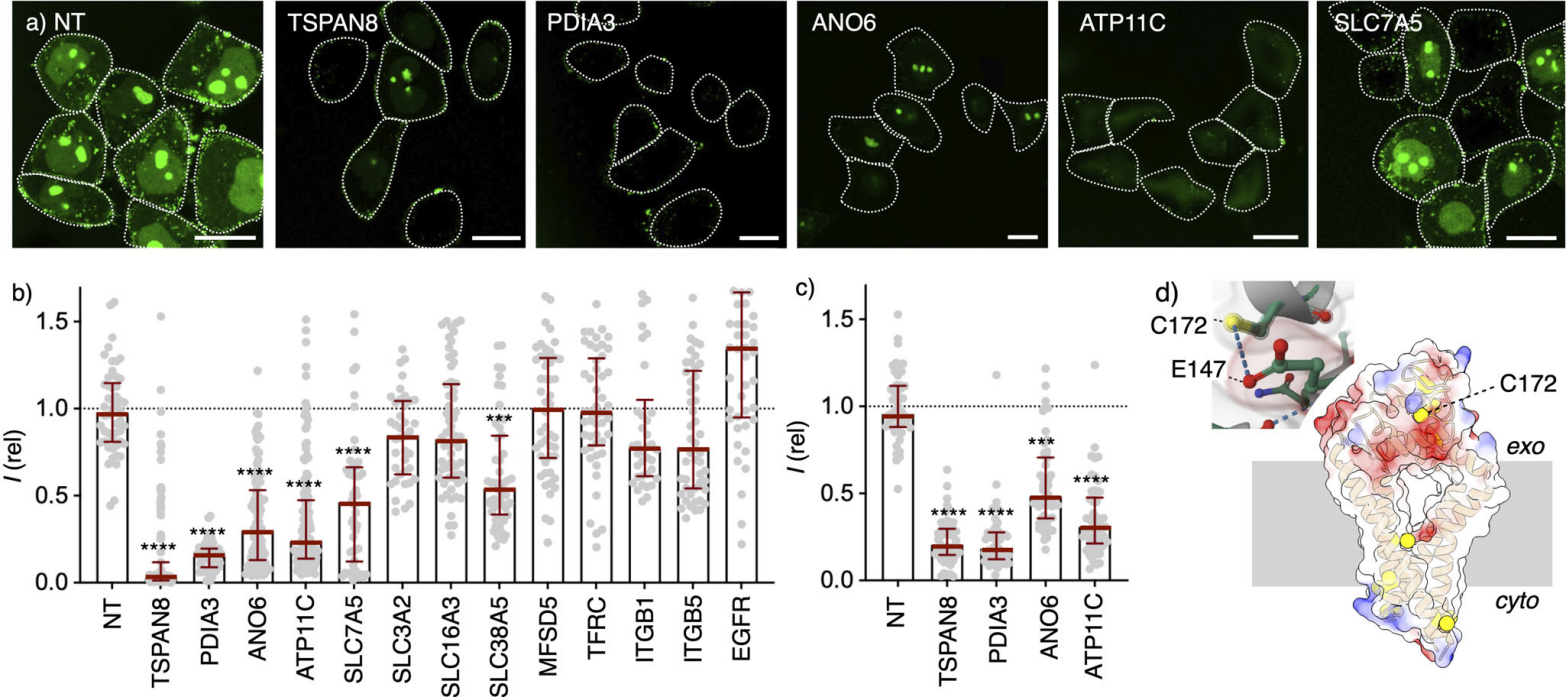
Response of CPDs to knockdown of TMU proteins. (a) CLSM images of Fl‐CPD **1** (75 nM, 20 min incubation, L15) in HK cells pretreated with siRNA against designated TMU proteins (NT: non‐targeted; scale bars = 20 µm). (b,c) Intracellular fluorescence intensity *I/I*_0_ (median ± interquartile range; *n* ∼ 50 cells) of non‐treated (*I* = *I*_0_) and siRNA‐treated HK cells incubated with Fl‐CPD **1** (b, 75 nM, 20 min) or GFP‐CPD **7** (c, 75 nM, 30 min, L15). Each gray point represents a value for one cell; *p* < 0.0001 (****), <0.001 (***) from nonparametric one‐way ANOVA, compared to the mean rank of the control data NT. (d) Electrostatic potential map of TSPAN8 (Swiss‐Model based on 8esv.1.B), red negative, blue positive, thiols in yellow, visualized using UCSF Chimera X [86]. Inset: zoomed structure around C172.

The uptake of Fl‐CPDs **1** was significantly affected by the KD of some previously identified TMU proteins (Figures 8, S26, and S27). Most notable uptake inhibition was seen in HK cells treated with siRNAs against TSPAN8 (Figure 8d, [86]), ATP11C, ANO6, PDIA3, and SLC7A5, while effects were negligible with most others, including integrin β‐1, which was the only significantly enriched TMU protein in µMap analyses (Figure 7). Similar effects of KD were also observed for the uptake of GFP‐CPDs **7** (Figure 8c). Nearly complete inhibition of CPD uptake was observed upon suppression of TSPAN8 or PDIA3, implying that both are required at different steps of the uptake.

Tetraspanin‐8 (TSPAN8) is a structural component of specialized membrane microdomains, which are believed to play various roles, including membrane damage repair [87], consistent with the proposed CPD uptake via toroidal elastics. It belongs to the TSPAN superfamily, which comprises 33 members characterized by conserved structural features and high overall abundance in the PM. Thus, the observed effect of KD on CPD uptake might be influenced by contributions from other TSPANs. The functional importance of flippase ATP11C as well as scramblase ANO6 supported the involvement of lipid binding and membrane reorganization in the CPD uptake mechanism. Reduced CPD uptake in siPDIA3‐treated HK cells was consistent with their sensitivity to PDI inhibitors (Figure 5). Whereas SLC7A5 was found to significantly affect CPD uptake, the adapter protein SLC3A2 was not, in line with its auxiliary function. Other SLCs and MFSD5 showed much weaker effects, despite sharing structural features, including numerous cysteines in transmembrane regions, suggesting a degree of selectivity.

### 2.9 The Mode of Action of CPDs

The combined results of this study added up to the following mode of action (Figure 9). CPDs bind to the cell surface by multiple ion pairing between guanidinium cations and cellular anions, such as peptide glycans (Figure 9a), and hop among cellular anion clusters. This dynamic accumulation on cell surfaces affords high effective CPD concentrations for dynamic covalent thiol/disulfide exchange cascades with membrane proteins (Figure 9b). The formation of dynamic covalent bonds immobilizes CPDs on the cell membrane, which contributes to membrane deformation and reorganization by, inter alia, assembling anionic lipids through repulsion‐driven ion pairing. Membrane reorganization and deformation ultimately lead to the emergence of transient elastic toroidal pores. The elastic nature of these micellar pores i) allows CPDs to pass through, also together with large substrates like proteins; ii) prevents content leakage; and iii) ensures fast repair after TMU (Figure 9c). Anionic lipids contributing to CPD binding, membrane deformation and CPD translocation are rare on the cell surface. However, they can be transferred from the inner PM leaflet by the scramblase ANO6 (Figure 9d) and maybe other membrane deforming proteins. ANO6, a member of the anoctamin family of 10 Cys‐rich membrane proteins in mammals, has been identified as TMU network component (Figure 8) and could explain Ca^2+^ sensitivity (Figures S28 and S29) and membrane reorganization, including exovesiculation (Figure 4).

**FIGURE 9:**
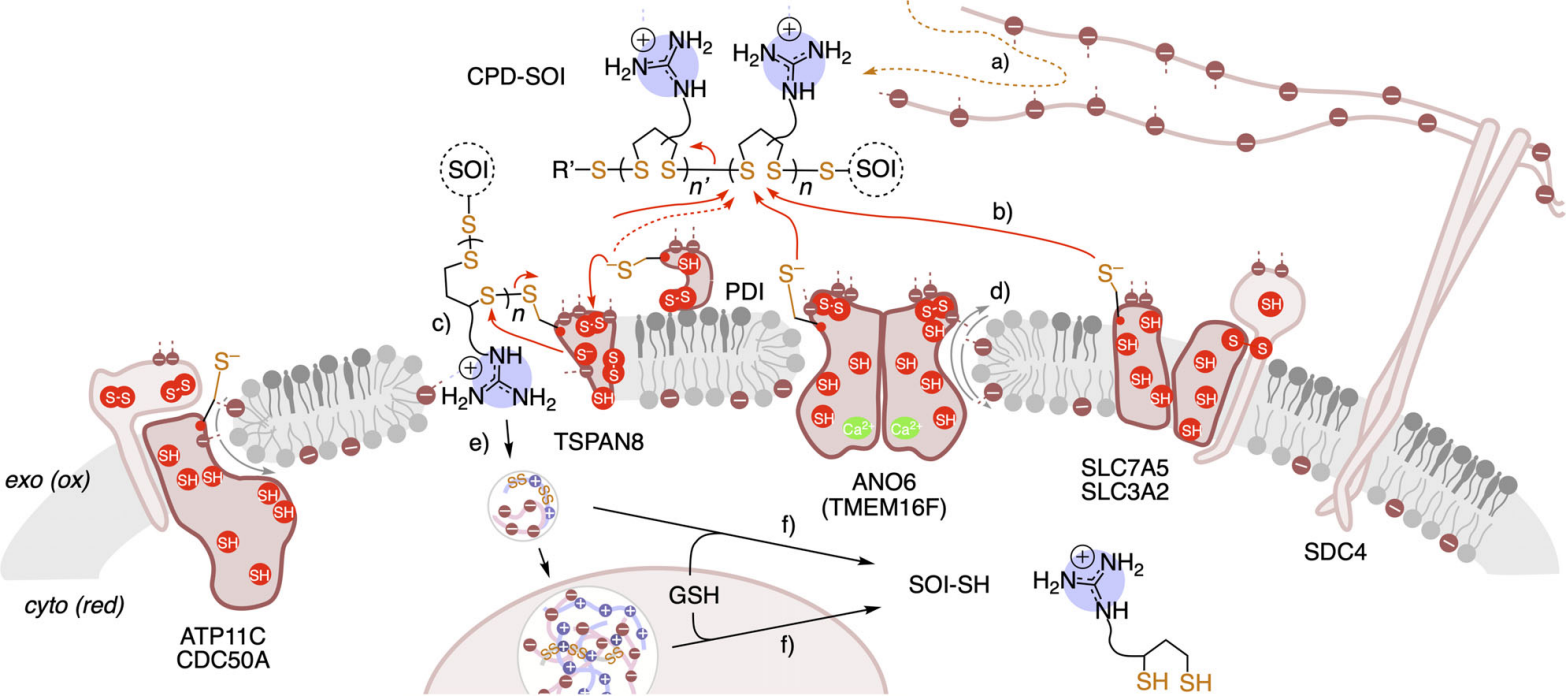
The mode of action of CPDs. (a) Attraction to cell surfaces by electrostatic interactions with polyanions, such as proteoglycans increases effective local CPDs concentration to promote (b) disulfide exchange with TMU proteins and (c) formation of toroidal elastic pores and related deformations to allow CPDs to cross the PM, assisted by disulfide exchange cascades and anionic lipids as counterions, (d) externalized by scramblases and related membrane reorganizers. In the cytosol, (e) CPDs form and bind to coacervates up to nucleoli by continuing counteranion exchange and (f) depolymerize with GSH to release native SOIs into the cytosol.

Initial electrostatic binding to cell surface anions (Figure 9a) was supported by the observation of CPP targets in µMap analyses (Figure 7), and the removability of CPDs by rinsing with heparin‐containing media (Figures 4d and S9). Heparin is a highly sulfated polysaccharide with structural similarities to heparan sulfate, which is a component of proteoglycans, including Syndecan‐4 (SDC4). Exploiting strong yet labile ionic interactions, multivalent counterion exchange with heparin has routinely been used to remove surface‐bound arginine‐rich CPPs [2, 29, 69]. The increased local concentration at the cell surface is probably the key to the high activity of CPDs, as it promotes the subsequent thiol/disulfide exchange reactions, which appear to require higher concentrations even with highly reactive strained disulfides (Figure 2). This interpretation was further consistent with the nonlinear uptake‐concentration dependence (Figures S7 and S8). Although dynamic covalent bonds formed by thiol/disulfide exchange (Figure 9b) should be kinetically more stable, µMap analyses failed to identify the proteins involved, probably because this process is transient (Figure 7). However, previously identified TMU proteins were confirmed to be significant for CPD uptake by KD (Figure 8), corroborating the occurrence of the TMU mechanism implied by the positive response to thiol‐reactive TMU inhibitors (Figure 5). It was satisfying to note that the most functionally significant proteins for CPD uptake are those involved in membrane remodeling, for example, TSPAN8, ANO6, and ATP11C, consistent with the toroidal elastic model (Figures 1bB and 9c). This model was further consistent with the reduced membrane order upon treatment with CPDs, reported by FLIM using fluorescent flippers (Figure 4). Moreover, this model explained the reported CPD inactivation at 4 °C by increased membrane ordering and reduced activity of membrane‐remodeling proteins [2].

All membrane‐active primary exchange partners feature polyanionic exocytic domains for multivalent ion pairing with the polycationic CPDs to enable, accelerate and direct disulfide exchange cascades. The strongest decrease in uptake upon KD of TSPAN8 made the membrane remodeling tetraspanin proteins top candidates for transporting CPDs across the PM by thiol/disulfide exchange with their multiple cysteines while stabilizing toroidal elastic pores (Figure 9c). In TSPAN8, the exocytic thiol of C172 is particularly well‐suited to initiate exchange cascades with CPDs since it is located near a negatively charged surface domain that should attract polycations and is activated as a dyad with hydrogen‐bonded E147 (Figure 8d). KD of TSPAN8 is likely to report also on contributions from other members of the TSPAN superfamily.

The flippase ATP11C and scramblase ANO6 were likely to operate similarly by transporting CPDs through hydrophilic, possibly elastic grooves intended for lipid flipping. Whereas PDIs were also functionally important for CPD uptake (Figures 5 and 8), they were less likely to transport CPDs across the PM, as judged by their structure, and are more likely to affect CPD uptake by regulating the redox state of proteins like TSPAN8.

After entering the cell, CPDs continue anion hopping, appear to form condensates with cytosolic polyanions like RNA (Figure 3), and merge with native condensates such as nucleoli (Figure 9e). While arginine‐rich CPPs also target and remain within nucleoli driven by similar multivalent ionic interactions [44, 88], the SOIs attached to CPDs can exit the nucleoli due to decreased multivalency following reductive depolymerization (Figures 3 and 9f). This process enables the unmodified, native SOIs to serve their intended function, making CPDs particularly useful.

## 3 Conclusions

CPDs are the most used TMU tool worldwide [7]. They deliver proteins at <50 nM concentrations [12], while small‐molecule CAXs and arginine‐rich CPPs usually require µM concentrations [22, 34, 36, 37, 38, 39, 40, 41]. The mode of action of CPDs was essentially unknown despite their power and popularity. The objective of this study was to understand how CPDs really work by applying lessons learned and a state‐of‐the‐art set of methods developed for other systems. Although the obtained results are rich and complex, the bottom line is quite straightforward: CPDs synergistically combine dynamic‐covalent disulfide exchange chemistry accounting for TMU with non‐covalent repulsion‐driven ion pairing accounting for the activity of arginine‐rich CPPs. With regard to TMU, CPDs are more powerful than small‐molecule CAXs because secondary interactions dominated by CPP‐like ion pairing strengthen non‐covalent binding to SOIs and cellular exchange partners to enable, accelerate and direct dynamic covalent exchange cascades for faster bioconjugation and higher TMU activity, respectively. Compared to arginine‐rich CPPs, CPDs are more active because the primary disulfide exchange partners enable, support and direct the membrane reorganization that affords the toroidal elastic pores and related membrane deformations for CPDs to pass together with SOIs as large as desired. Within cells, CPDs form coacervates also in the cytosol and fuse with nucleoli. Then, contrary to arginine‐rich CPPs, they depolymerize and release the native SOIs.

The elucidation of the nature of CPDs clarifies and completes the general understanding of TMU, particularly regarding the acceleration of exchange cascades with non‐covalent secondary interactions and the formation of condensates within cells. Most important is the dynamic covalent coupling to membrane‐reorganizing exchange partners to exploit their ability to mobilize anionic lipids and to form and repair transient toroidal elastic pores for cellular entry (TSPANs, ANO6, ATP11C/CDC50A, SLCs, Figure 9). This understanding will guide future efforts to enable TMU, particularly CPDs, to predictably control cellular entry in practice for the delivery of chemicals and for defense against pathogens, thus contributing new approaches to tackle these challenges of central importance in science and society.

## Author Contributions

S.S., G.R., Y.Z., M.C., and J.N. synthesized. S.S., G.R., M.C., and N.S. characterized the probes. A.H., N.S., and S.M. directed the study. All authors contributed to the design of experiments, data interpretation and manuscript writing.

## Conflicts of Interest

The authors declare the following competing financial interest: The University of Geneva has licensed four Flipper‐TR probes to Spirochrome for commercialization.

## Supporting information




**Supporting File**: anie74965‐sup‐0001‐SuppMat.pdf.

## Data Availability

The data that support the findings of this study are openly available at http://doi.org/10.5281/zenodo.22208502.
