# Supplementary material for: The Nature of Cell‐Penetrating Poly(disulfide)s
Source: Angew Chem Int Ed Engl. 2026 Sep 26;65(46):e7902673. doi: 10.1002/anie.7902673 (PMC13749373; doi:10.1002/anie.7902673)
Supplement: Supplementary file 1 [file ANIE-65-e7902673-s001.pdf]

# Supporting Information

**The Nature of Cell-Penetrating Poly(disulfide)s**

Saidbakhrom Saidjalolov[1,2] | Giacomo Renno[1,2] | Yuheng Zhang[1,2] | Michael Cognet[1,2] | Justin Neumann[3] | Andreas Hennig[3] | Naomi Sakai[1,2] | Stefan Matile[1,2]

[1]Department of Organic Chemistry, University of Geneva, Quai Ernest Ansermet 30, 1211 Geneva 4, Switzerland

[2]National Centre of Competence in Research (NCCR) Molecular Systems Engineering, BPR 1095, 4002 Basel, Switzerland

[3]Center for Cellular Nanoanalytics (CellNanOs) and School of Biology / Chemistry, Universität Osnabrück, Barbarastraße 7, 49069 Osnabrück, Germany

Correspondence: naomi.sakai@unige.ch, stefan.matile@unige.ch

## Table of Contents


| | | |
|---|---|---|
| 1. | Materials and Methods | S4 |
| 2. | Compounds | S7 |
| 3. | Synthesis | S9 |
| 3.1. | Synthesis of CAX Transporters and Inhibitors | S9 |
| 3.2. | Synthesis of Initiators and Monomers | S10 |
| 3.3. | Synthesis of Proteomics Components | S14 |
| 4. | General Procedures of CPD Synthesis | S15 |
| 5. | CPD Characterization | S16 |
| 6. | CPD Stability in Leibovitz L15 Medium | S19 |
| 7. | Cell Culture | S20 |
| 8. | Lysate Preparation | S20 |
| 9. | Western Blot Analysis | S20 |
| 10. | Cellular Uptake | S21 |
| 10.1. | Confocal Laser Scanning Microscopy | S21 |
| 10.2. | Data Analysis | S22 |
| 10.3. | Optimization of Cellular Uptake | S23 |
| 10.4. | CPD Extraction with Heparin | S24 |
| 11. | Inhibition of CPDs | S24 |
| 11.1. | TMU Inhibitors | S24 |
| 11.2. | PDI Inhibitors | S27 |
| 12. | CPP Activators | S28 |
| 13. | CPD Depolymerization | S29 |
| 13.1, | CPD Depolymerization in Condensates | S29 |
| 13.2. | CPD Depolymerization in Cells | S32 |

14. Membrane Reorganization by CPDs S32

14.1. Membrane Reorganization in GUVs S32

14.2. Membrane Reorganization in HK Cells S33

15. Uptake Validation of Proteomics Probes S34

16. μMap Proteomics S35

16.1. General Procedure S35

16.2. Data Acquisition S36

16.3. Data Analysis S37

17. Protein Knockdown S38

17.1. Level of Expression S38

17.2. STRING Analysis S39

17.3. Knockdown Procedure S39

17.4. Cellular Uptake in Knocked-Down Cells S40

18. Calcium Dependence S41

19. Supporting References S42

20. NMR Spectra S44

## 1. Materials and Methods

As described in reference [S1], reagents for synthesis were purchased from Merck, Sigma-Aldrich, TCI, Broadpharm, and Fluorochem. High-quality salts from Fluka or Sigma-Aldrich were used without further purification. The following media and solutions were obtained from Thermo Fisher Scientific: phosphate-buffered saline (PBS, pH 7.4), Dulbecco's PBS (calcium/magnesium-free, DPBS, 14190094), DMEM (11965092), FluoroBrite DMEM (GlutaMAX, 4.5 g/L D-glucose, pyruvate, without phenol red, A1896701), Opti-MEM (11058021), and Leibovitz's L15 (without phenol red, 21083027). Other reagents and consumables from the same supplier included penicillin-streptomycin, fetal calf serum, and TrypLE Express enzyme, Streptavidin Alexa Fluor® 680 conjugate (S21378), Dynabeads M-280 streptavidin (11206D), and Lipofectamine RNAiMAX (13778075). 35 mm glass-bottom culture dishes were purchased from MatTek Dishes, 8- and 18 well µ-slides with glass coverslip bottom were from ibidi. 1-Palmitoyl-2-oleyl-phosphatidylcholine (POPC), 1-Palmitoyl-2-oleyl-phosphatidylglycerol (POPG), and egg sphingomyelin (SM) were purchased from Avanti Research™. BAPTA-AM was from Cayman Chemical. Sodium dodecabromododecaborate (**22**) was from Katchem. siRNA solutions for protein knockdown were purchased from siTOOLs Biotech. Fluorescent conjugate secondary antibody (Alexa Fluor® 647 (711-605-152), AffiniPure Donkey Anti-Rabbit IgG (H+L)) was purchased from Jackson ImmunoResearch. Analytical thin-layer chromatography (TLC) was conducted on silica gel 60 F254 (Merck, 0.2 mm), visualized under UV light at 254 nm. Flash chromatography was done using a Biotage Isolera™ Spektra or Selekt system with pre-packed Scorpius cartridges (BGB). Melting points (Mp) were recorded using a Melting Point M-565 (BUCHI). IR spectra were obtained using a Perkin Elmer Spectrum Two™ FT-IR spectrometer (ATR, Golden Gate), reported as wavenumbers ($\nu$) in $cm^{-1}$ with intensities described as broad (br), strong (s), medium (m), or weak (w). $^{1}H$, $^{13}C$, and $^{19}F$ NMR spectra were recorded on Bruker 300, 400, or 500 MHz spectrometers at 25 °C, with chemical shifts ($\delta$) in parts per million (ppm) referenced to residual solvent peaks (DMSO-$d_6$:

2.50/39.5 ppm; $CD_3OD$: 3.31/49.0 ppm; $CDCl_3$: 7.26/77.2 ppm). Spin multiplicities were reported as singlet (s), doublet (d), triplet (t), or multiplet (m), with coupling constants (*J*) in Hz. $^1H$ and $^{13}C$ assignments were supported by 1D and 2D NMR spectra ($^1H$-$^1H$ COSY, DEPT 135, HSQC, HMBC). ESI-HRMS was performed on a Xevo G2-S TOF (Waters). LC-MS (low resolution) analysis was conducted on an Advion Avant® UHPLC system with a Thermo C18 Hypersil GOLD column (50 x 2.1 mm, 1.9 µm, 0.75 mL/min, gradient elution of $H_2O$ + 0.01% TFA / $CH_3CN$ + 0.01% TFA from 3:7 to 0:1 in 4.0 min) and an Advion Expression® CMS in ESI mode. All mass data are presented as mass-to-charge ratios (*m/z*). UV-Vis spectra were recorded on a JASCO V-650 spectrophotometer with a stirrer and temperature controller (20 °C), with maximal absorption wavelengths ($\lambda$) in nm and extinction coefficients ($\varepsilon$) in $M^{-1}$ $cm^{-1}$. Fluorescence spectra were collected on a FluoroMax-4 (Horiba Scientific), with correction factors applied. Proteome analysis was performed at the Proteomics Core Facility, Faculty of Medicine, University of Geneva, on an Easy Nano LC - Orbitrap Fusion System equipped with a nanospray flex™ ion source (Thermo Fisher Scientific, USA). Confocal laser scanning microscopy (CLSM) was performed on Leica SP8, equipped with 63x oil immersion objective or 20x water immersion objective. Fluorescence lifetime imaging microscopy (FLIM) images were acquired using Leica Stellaris 8 Falcon with a 63× oil immersion lens. Fluorescence was collected between 550 and 650 nm upon excitation with a white laser set at 488 nm and 20 MHz. The images were analyzed using Leica Application Suite Software LASX FLIM 4.8. Lifetime ($t$) was estimated by manually choosing the top of the phasor cloud.

**Abbreviations.** AspA: Asparagusic acid; BCA: Bicinchoninic acid; BSA: Bovine serum albumin; CAX: Covalent exchangers; CDI: Carbonyldiimidazole; CL: Cholesterol; CLSM: Confocal laser scanning microscopy; $c_M$: Monomer concentration; CPDs: Cell-penetrating polydisulfides; CPPs: Cell-penetrating peptides; DIPEA: *N*,*N*-Diisopropylethylamine; dMAC: Double Michael acceptor; DMEM: Dulbecco's modified eagle medium; DMF: *N*,*N*-dimethylformamide; DMSO: Dimethyl sulfoxide; DPBS: Dulbecco's phosphate-buffered saline; DTNB: (5,5′-Dithiobis-(2-

nitrobenzoic acid); DTT: Dithiothreitol; ETP: Epidithiodiketopiperazine; FA: Formic acid; FCS: Fetal calf serum; FDMEM: FluoroBrite DMEM; FITC: Fluorescein isothiocyanate; Fl-: Fluorescein; FLIM: Fluorescence lifetime imaging microscopy; GSH: Glutathione; GUV: Giant unilamellar vesicle; HATU: Hexafluorophosphate azabenzotriazole tetramethyl uronium; HeLa: Henrietta Lacks; HK: HeLa Kyoto; HPLC: High-Performance Liquid Chromatography; HRMS: High resolution mass spectra; $IC_{50}$: Half maximal inhibitory concentration; KD: Knockdown; LLPS: Liquid-liquid phase separation; LRMS: Low resolution mass spectra; MIC: Minimum inhibitory concentration; NHS: *N*-Hydroxysuccinimide; PBS: Phosphate-buffered saline; pc: Photocatalyst; PFA: Paraformaldehyde; POPC: 1-Palmitoyl-2-oleyl-phosphatidylcholine; POPG: 1-Palmitoyl-2-oleyl-phosphatidylglycerol; PS: Penicillin-streptomycin; RIPA buffer: Radioimmunoprecipitation assay buffer; RP: Reverse-phase; RT: Room temperature; RV: Relative viability; SAV680: Streptavidin-AlexaFluor680; SD: Standard deviation; SEC: Size exclusion chromatography; SEM: Standard error of mean; siRNA: Small interfering RNA; SM: egg-sphingomyelin; TCEP: Tris(2-carboxyethyl)phosphine; TEA: Triethylamine; TEOA: Triethanolamine; TFA: Trifluoroacetic acid; THF: Tetrahydrofuran; THPTA: Tris(hydroxypropyltriazolyl-methyl)amine; TIPS: Triisopropylsilane; TLC: Thin layer chromatography; TMU: Thiol-mediated uptake; TRIS buffer: Tris(hydroxymethyl)aminoethane buffer; WB: Western blot; WI: Water immersion; WT: Wild type.

## 2. Compounds

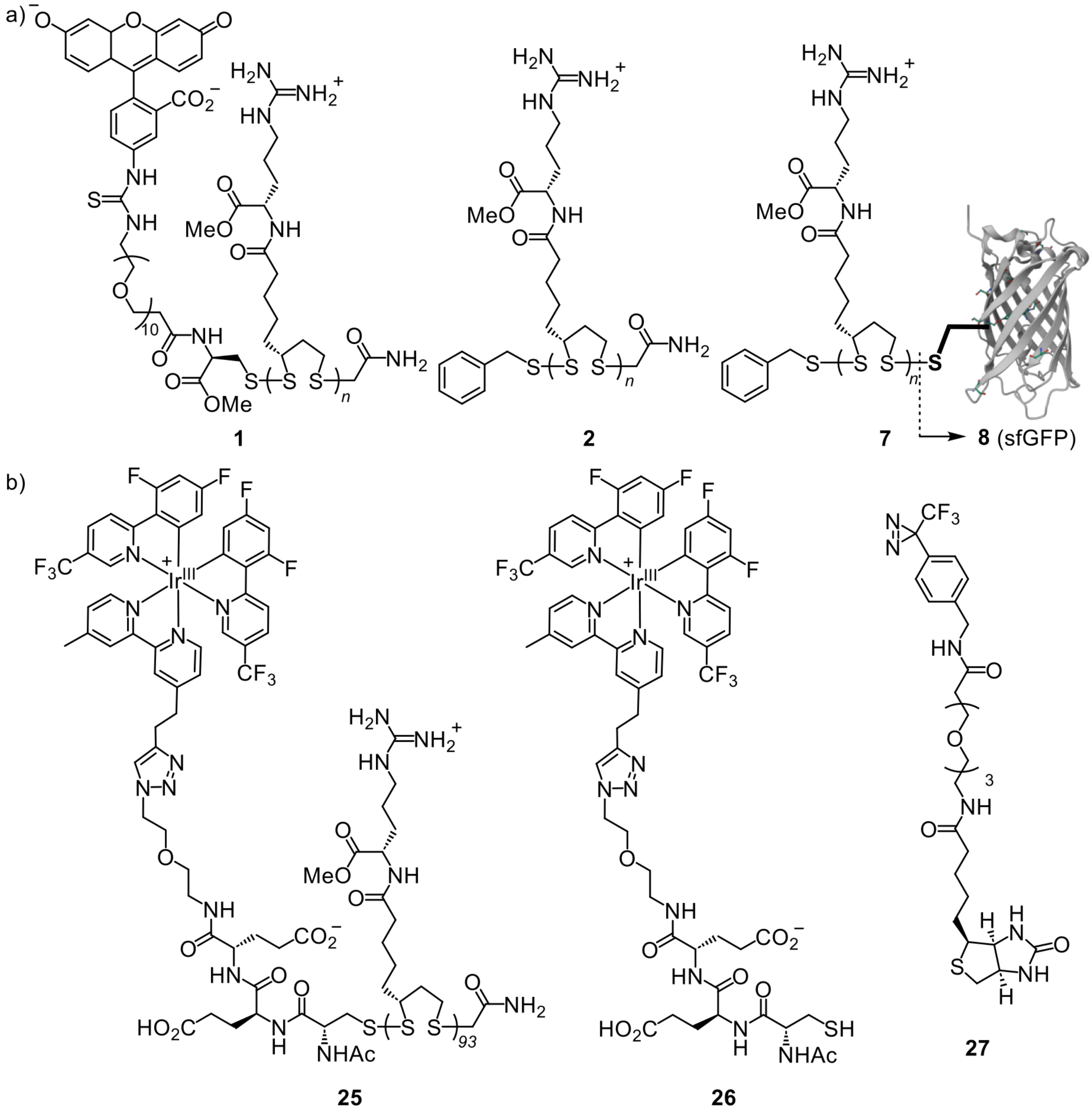


**Figure S1** | Structure of compounds used in this study (part 1): (a) CPDs and (b) probes for μMap proteomics.

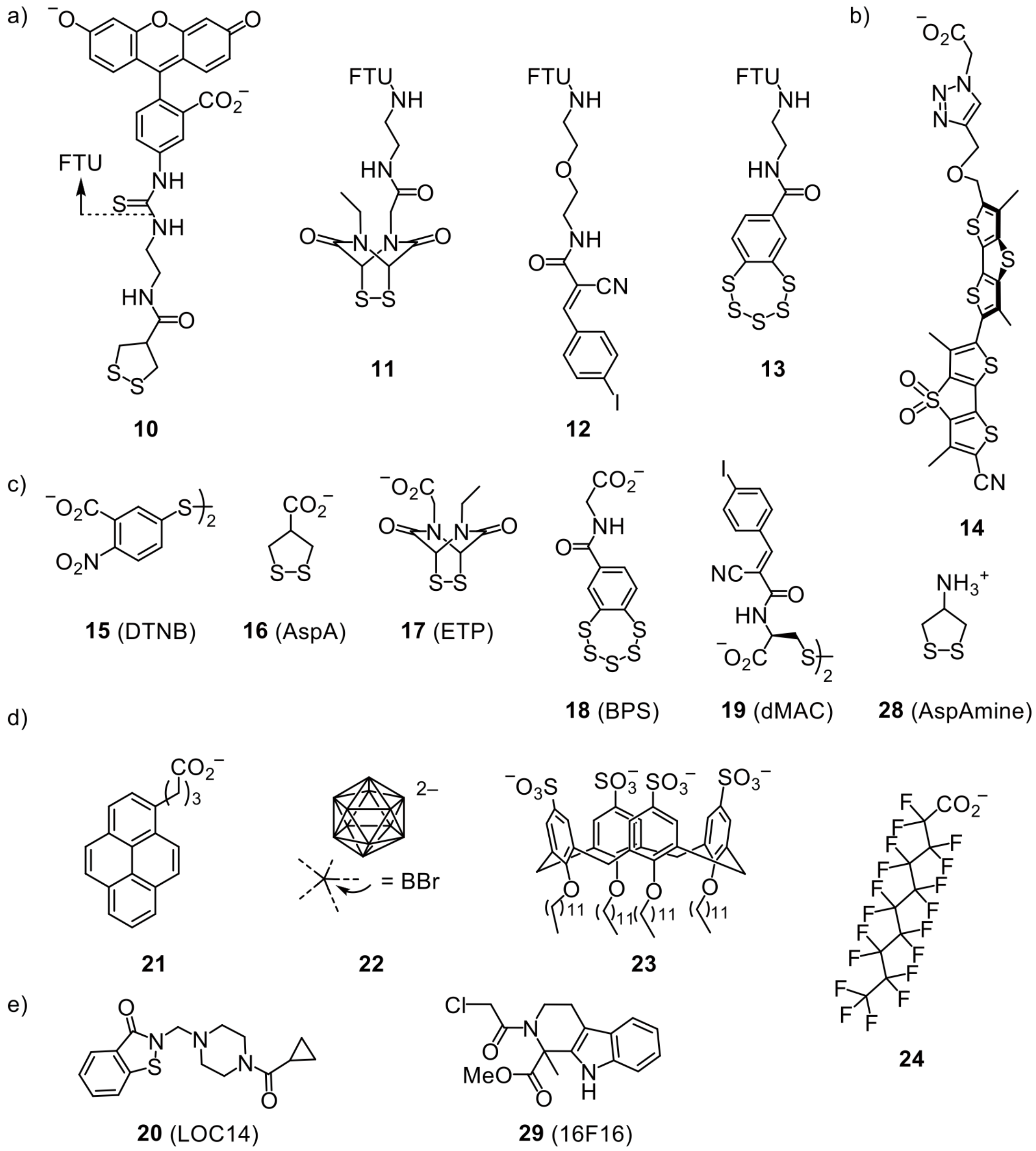


**Figure S2** | Structure of compounds used in this study (part 2): (a) TMU transporters, (b) Flipper-TR®, (c) TMU inhibitors, (d) CPP activators and (e) PDI inhibitors.

**sfGFP S147C Mutant 8** with the following amino acid sequences was produced at the Protein Biochemistry Platform, Faculty of Medicine, University of Geneva, Switzerland.

MSKGEELFTG VVPILVELDG DVNGHKFSVR GEGEGDATNG KLTLKFICTT GKLPVPWPTL VTTLTYGVQC FSRYPDHMKR HDFFKSAMPE GYVQERTISF KDDGTYKTRA EVKFEGDTLV NRIELKGIDF KEDGNILGHK LEYNFNCHNV YITADKQKNG IKANFKIRHN VEDGSVQLAD HYQQNTPIGD GPVLLPDNHY LSTQSVLSKD PNEKRDHMVL LEFVTAAGIT HGMDELYK.

**Calixarene 23** was prepared as described in reference [S2].

## 3. Synthesis

### 3.1. Synthesis of CAX Transporters and Inhibitors

**Compound 10** was synthesized according to procedures described in reference [S3].

**Compound 11** was synthesized according to procedures described in reference [S4].

**Compound 12** was synthesized according to procedures described in reference [S5].

**Compound 13** was synthesized according to procedures described in reference [S6].

**Compound 16** was synthesized according to procedures described in reference [S3].

**Compound 17** was synthesized according to procedures described in reference [S4].

**Compound 18** was synthesized according to procedures described in reference [S6].

**Compound 19** was synthesized according to procedures described in reference [S5].

## 3.2. Synthesis of Initiators and Monomers

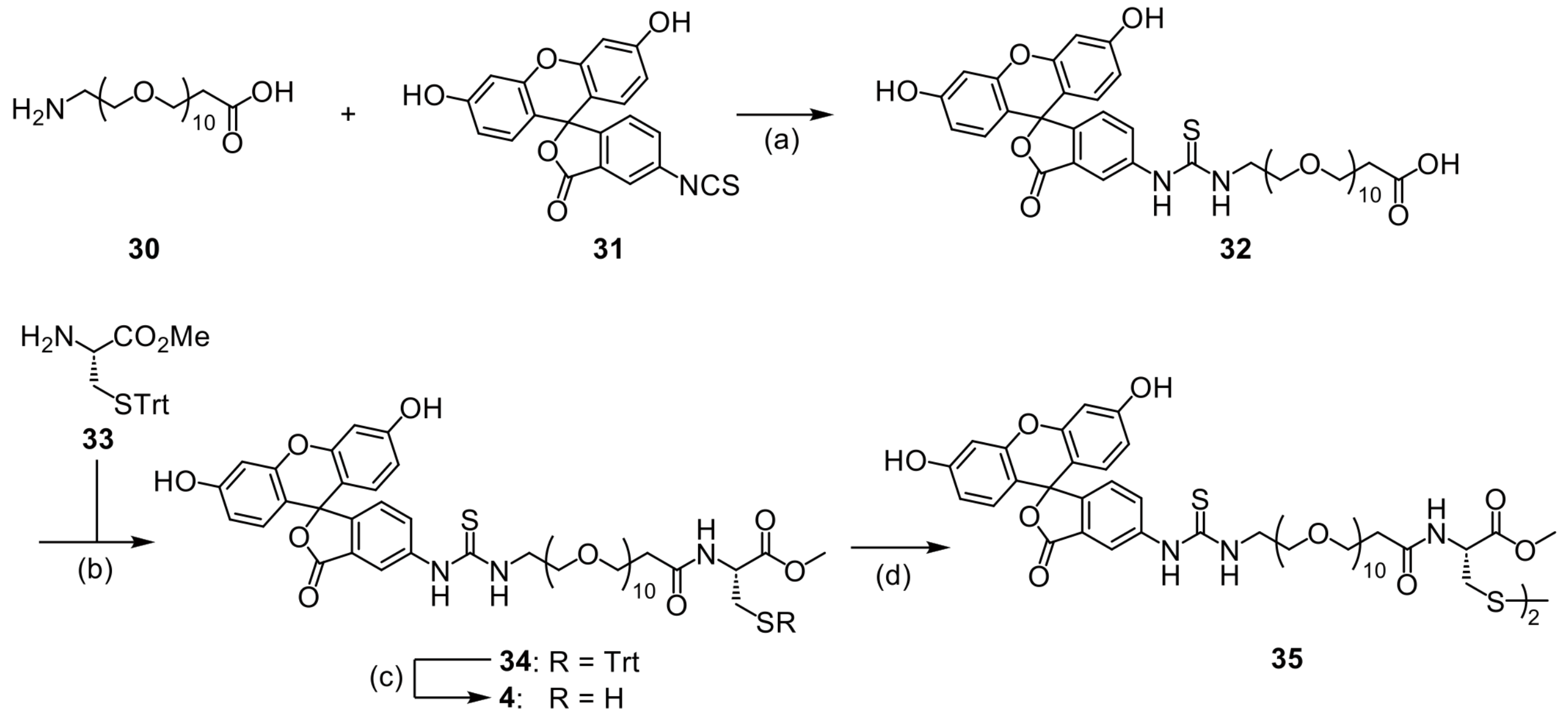


**Scheme S1** | (a) DIPEA, $CH_2Cl_2$/DMF 1:1, RT, 2 h, 90%; (b) DIPEA, HATU, $CH_2Cl_2$/DMF 1:1, RT, 2 h, 72%; (c) TFA, TIPS, $CH_2Cl_2$, 0 °C to RT, 30 min, 78%; (d) DMSO, RT, 24 h, quant.

**Compounds 32**, **34**, **4** and **35** were synthesized according to procedures described in reference [S7].

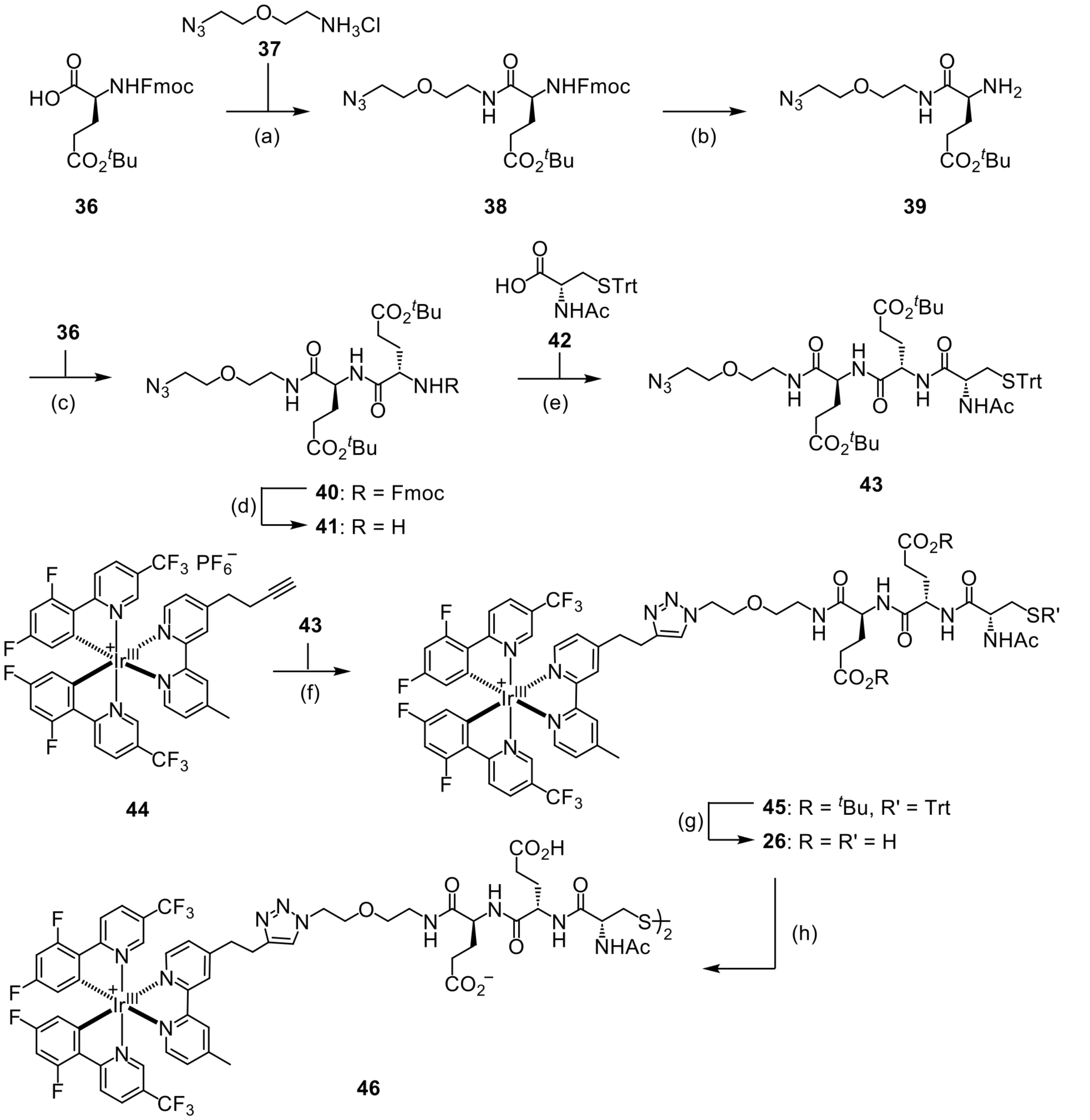

**Scheme S2** | (a) HATU, DIPEA, $CH_2Cl_2$, 45 min, 0 to RT, 72%; (b) $NHMe_2$ 2 M THF, 1 h, RT, 99%; (c) HATU, DIPEA, $CH_2Cl_2$, 1.25 h, 0 to RT, 85%; (d) $NHMe_2$ 2 M THF, 1 h, RT, quant; (e) HATU DIPEA, DMF, 4 h, RT, 94%; (f) CuI, THPTA, THF, 2 h, RT; (g) TFA, TIPS, $CH_2Cl_2$, 1 h, RT, 41% over 2 steps; (h) DMSO, RT, 24 h, quant.

**Compounds 38–41** and **44** were synthesized according to procedures described in reference [S8].

**Compound 43.** To a solution of **42** (39 mg, 96 µmol) in DMF (1 mL) was added in one portion HATU (30 mg, 80 µmol) and DIPEA (28 µL, 160 µmol). The reaction mixture was stirred for 5 min at RT. Following this, **41** (40 mg, 80 µmol) was added, and the reaction mixture was stirred for 4 h at RT (completion monitored by TLC). The crude mixture was diluted in EtOAc (50 mL) and washed with 1 M HCl (50 mL), $NaHCO_3$ sat. (50 mL), and brine (50 mL), dried over $Na_2SO_4$, and concentrated *in vacuo*. The crude solid was subjected to purification by flash chromatography (silica gel, 12 g, gradient $CH_2Cl_2$/MeOH 100:0 to 95:5), followed by trituration with ether (x2) to afford compound **43** (67 mg, 94%) as a colorless solid. $R_f$ ($CH_2Cl_2$/MeOH 98:2): 0.2; Mp: > 115 °C decomposed; IR (neat): 3280 (w, NH), 2977 (w, C-H), 2917 (w, C-H), 2850 (m, C-H), 2012 (w, $N_3$), 1726 (w, C=O), 1628 (m, C=O), 1520 (s, C-N-H), 1444 (m, $CH_2$/$CH_3$), 1392 (m, $^tCH_3$), 1366 (w), 1254 (w, C-O), 1185 (w, C-O-C), 1149 (s, C-N), 1034 (s, C-N), 848 (s), 740 (s, C-O-C), 699 (s, C-S), 622 (w), 597 (w); $^1$H NMR (400 MHz, $CDCl_3$): 7.74 (d, $^3J_{H-H}$ = 5.8 Hz, 1H), 7.49 – 7.38 (m, 6H), 7.36 – 7.29 (m, 6H), 7.28 – 7.18 (m, 5H), 6.83 (t, $^3J_{H-H}$ = 5.6 Hz, 1H), 5.70 (d, $^3J_{H-H}$ = 5.3 Hz, 1H), 4.39 – 4.33 (m, 1H), 4.29 – 4.23 (m, 1H), 4.06 – 4.00 (m, 1H), 3.63 (t, $^3J_{H-H}$ = 5.6 Hz, 2H), 3.57 (d, $^3J_{H-H}$ = 5.6 Hz, 2H), 3.44 (q, $^3J_{H-H}$ = 5.6 Hz, 2H), 3.41 – 3.33 (m, 2H), 2.87 (dd, $^2J_{H-H}$ = 13.1 Hz, $^3J_{H-H}$ = 4.8 Hz, 1H), 2.61 (dd, $^2J_{H-H}$ = 13.1 Hz, $^3J_{H-H}$ = 4.8 Hz, 1H), 2.44 – 2.32 (m, 2H), 2.31 – 2.12 (m, 3H), 2.07 – 1.86 (m, 6H), 1.43 (s, 9H), 1.42 (s, 9H); $^{13}$C NMR (126 MHz, $CDCl_3$): 174.4 (2C), 172.6 (C), 171.3 (C), 171.1 (C), 170.9 (C), 144.3 (3C), 129.6 (6CH), 128.4 (6CH), 127.2 (3CH), 81.6 (C), 80.7 (C), 69.8 ($CH_2$), 69.6 ($CH_2$), 67.7 (C), 54.5 (CH), 53.5 (CH), 53.1 (CH), 50.9 ($CH_2$), 39.4 ($CH_2$), 33.0 ($CH_2$), 32.3 ($CH_2$), 32.1 ($CH_2$), 28.2 ($6CH_3$), 27.0 ($CH_2$), 26.1 ($CH_2$), 23.2 ($CH_3$); LRMS (ESI): 888 ($[M+H]^+$)

**Compound 26.** To a solution of **44** (50 mg, 47 µmol) and **43** (54 mg, 60 µmol) in dry THF (5 mL) was added in one portion copper(I) iodide (18 mg, 93 µmol) and THPTA (10 mg, 23 µmol). The reaction mixture was stirred for 1 h at RT (completion evidenced by TLC). The copper iodide was removed by filtration over celite and THF was evaporated *in vacuo*. The obtained crude solid

was subjected to purification by flash chromatography (silica gel 25 g, gradient $CH_2Cl_2$/MeOH 10:0 to 9:1) to afford the triazole product, which was subsequently dissolved in $CH_2Cl_2$ (4 mL) and TFA (1 mL) was added dropwise to the solution. After the addition of TIPS (7 mg, 47 µmol), the reaction mixture was stirred for 1 h at RT (completion evidenced by LC-MS). $CH_2Cl_2$ was evaporated, and the crude oil was triturated in $Et_2O$. The crude mixture was dissolved in DMF (500 µL) and subjected to purification by RP chromatography ($C_{18}$ 33 g, linear gradient $CH_3CN$ + 0.1% TFA 10% to 100%). The fractions containing the product were collected and lyophilized to afford compound **26** TFA salt (28 mg, 41% over 2 steps) as a yellow powder. $R_f$ ($CH_2Cl_2$/MeOH 9:1): 0.3; Mp: > 122 °C decomposed; IR (neat): 3287 (w, N-H), 3073 (w, C-H), 2938 (w, C-H), 2161 (w), 1980 (w), 1660 (m), 1601 (s, C=O), 1567 (m, C=C), 1494 (w), 1424 (w), 1386 (w), 1329 (s, C-N), 1298 (s, C-N), 1167 (s), 1132 (s, C-O-C), 1108 (s, C-F), 1090 (s, C-F), 1051 (m), 991 (s), 920 (w), 831 (s), 797 (s), 720 (s), 705 (s), 615 (w), 528 (w); $^1$H NMR (400 MHz, DMSO-$d_6$): 8.89 (d, $^4J_{H\text{-}H}$ = 1.8 Hz, 1H), 8.84 (d, $^4J_{H\text{-}H}$ = 1.8 Hz, 1H), 8.50 – 8.38 (m, 4H), 8.17 (d, $^3J_{H\text{-}H}$ = 7.5 Hz, 1H), 8.13 (d, $^3J_{H\text{-}H}$ = 7.5 Hz, 1H), 7.88 – 7.80 (m, 5H), 7.67 – 7.64 (m, 2H), 7.58 (dd, $^3J_{H\text{-}H}$ = 5.8 Hz, $^4J_{H\text{-}H}$ = 1.7 Hz, 1H), 7.52 – 7.51 (m, 1H), 7.06 – 7.03 (m, 2H), 5.79 – 5.75 (m, 2H), 4.45 (t, $^3J_{H\text{-}H}$ = 5.3 Hz, 2H), 4.38 – 4.30 (m, 1H), 4.25 – 4.17 (m, 2H), 3.75 (t, $^3J_{H\text{-}H}$ = 5.3 Hz, 2H), 3.38 (t, $^3J_{H\text{-}H}$ = 5.3 Hz, 2H), 3.28 – 3.14 (m, 4H), 3.14 – 3.04 (m, 3H), 2.81 – 2.60 (m, 2H), 2.58 (s, 3H), 2.36 (t, $^3J_{H\text{-}H}$ = 8.5 Hz, 1H), 2.31 – 2.13 (m, 4H), 1.96 – 1.82 (m, 5H), 1.81 – 1.65 (m, 2H), 0.89 – 0.83 (m, 1H); $^{13}$C NMR (126 MHz, DMSO-$d_6$): 174.0 (C), 173.9 (C), 171.0 (C), 170.2 (C), 169.9 (C), 169.7 (C), 166.8 (2C), 164.9 (dd, $^1J_{C\text{-}F}$ = 257.4 Hz, $^3J_{C\text{-}F}$ = 12.7 Hz, 2C), 162.8 (dd, $^1J_{C\text{-}F}$ = 257.4 Hz, $^3J_{C\text{-}F}$ = 12.7 Hz, 2C), 160.71 (d, $^2J_{C\text{-}F}$ = 6.3 Hz, 2C), 158.1 (q, $^2J_{C\text{-}F}$ = 35.1 Hz, TFA), 155.7 (C), 155.3 (d, $^2J_{C\text{-}F}$ = 6.3 Hz, 2C), 155.0 (C), 150.3 (CH), 150.0 (CH), 145.5 (CH), 145.2 (C), 145.1 (CH), 137.6 (2CH), 129.7 (CH), 129.0 (CH), 126.5 (2C), 126.0 (CH), 125.2 (CH), 124.6 (q, $^2J_{C\text{-}F}$ = 35.1 Hz, 2C), 123.8 (CH), 123.6 (CH), 122.5 (CH), 121.9 (q, $^1J_{C\text{-}F}$ = 272.2 Hz, 2C), 114.1 (t, $^2J_{C\text{-}F}$ = 15.2 Hz, 2C), 99.6 (t, $^2J_{C\text{-}F}$ = 27.7 Hz, 2C), 68.6 ($2CH_2$), 55.2 (CH), 52.2 (CH), 52.1 (CH), 49.2 ($CH_2$), 38.3 ($CH_2$), 34.1 ($CH_2$), 30.6 ($2CH_2$),

27.0 (2CH$_2$), 26.0 (CH$_2$), 25.2 (CH$_2$), 22.5 (CH$_3$), 20.9 (CH$_3$);$^{19}$F NMR (282 MHz, DMSO-$d_6$): -61.5 (CF$_3$), -61.6 (CF$_3$), -74.5 (TFA), -103.3 – -103.4 (m, 2F), -106.8 (dd, $J$ = 12.0, 5.8 Hz, 2F).

**Compound 46.** Compound **26** was diluted in DMSO to make a stock solution for further experiments. Over time (~ 24 h, full conversion), the dimer **46** was formed by spontaneous oxidation. HRMS (ESI, +ve): calcd for $C_{116}H_{108}F_{20}Ir_2N_{22}O_{18}S_2$ [M+2H]$^{2+}$: 1461.3268, found: 1461.3259.

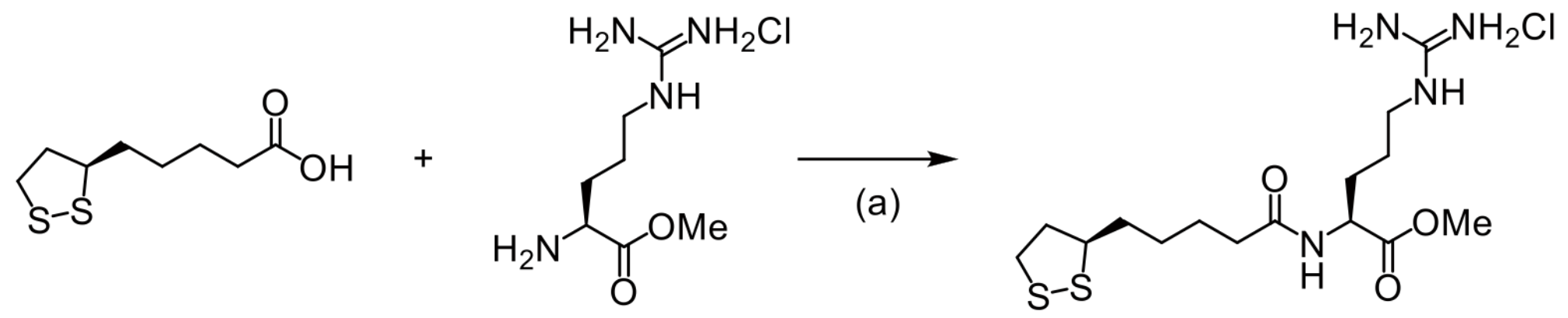


**Scheme S3** | (a) CDI, DMF, RT, 3.5 h, 84%.

**Compound 3** was synthesized according to procedures described in reference [S9].

## 3.3. Synthesis of Proteomics Components

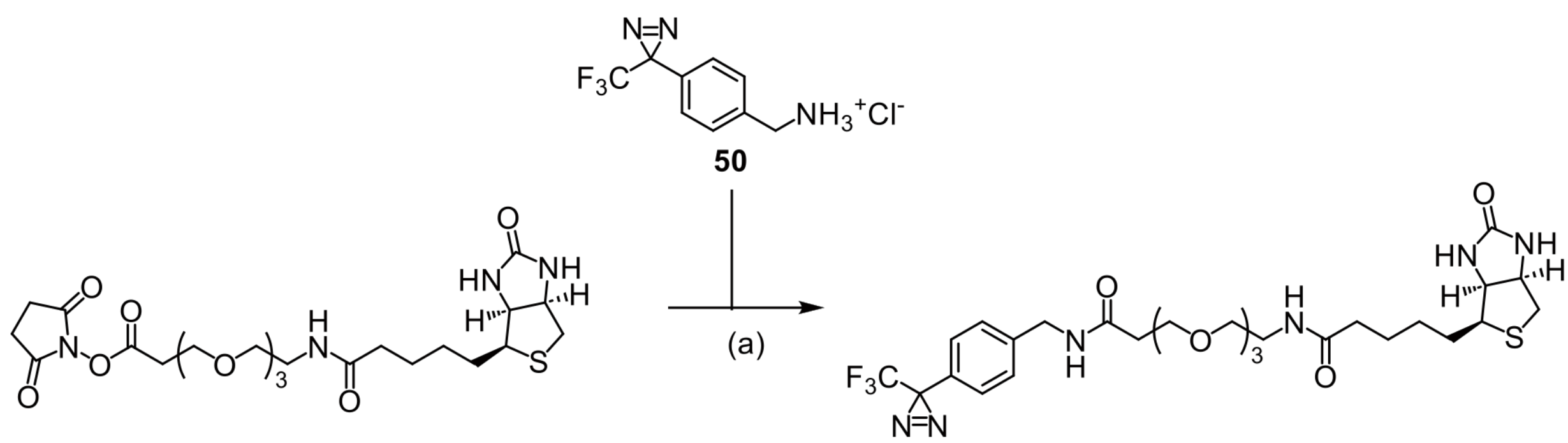


**Scheme S4** | (a) DIPEA, DMF, RT, 18 h, 92%.

**Compound 27** was synthesized according to procedures described in reference [S8].

## 4. General Procedures of CPD Synthesis

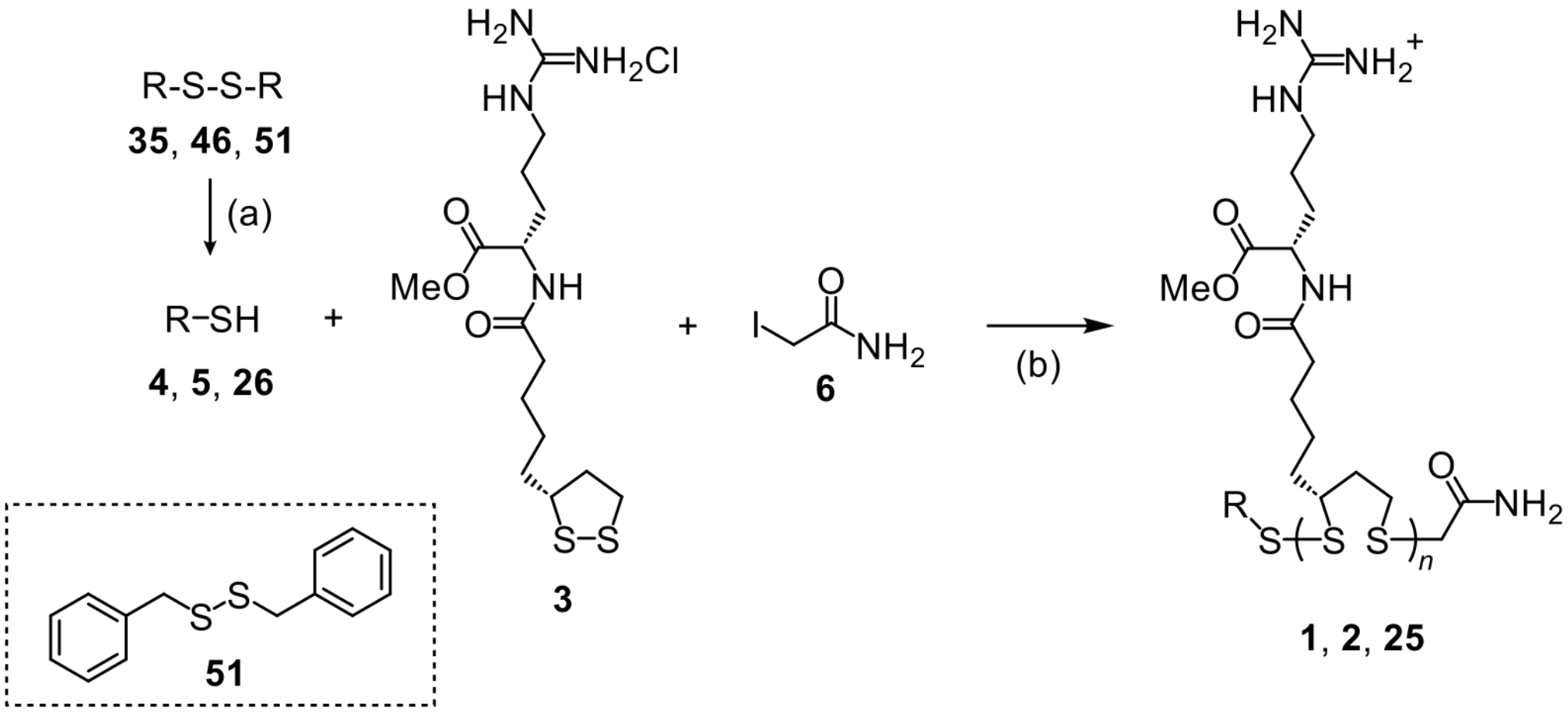


**Scheme S5** | (a) TCEP, TEOA buffer/DMSO 1:1, 25 °C, 15 min; (b) 1. TEOA buffer/DMF 8:2, 25 °C, 30 min; 2. **6**, 25 °C, 15 min. Regioregularity of CPDs not determined, expected to be almost randomly mixed 3:2 regio-irregular polymers.

*Polymerization through "Grafting-from".* The CPDs were synthesized following described procedures [S10]. Briefly, a stock solution of initiator **4**, **5** or **26** was prepared by pre-treating a solution of dimers **35**, **46** or **51** (4 µL of 50 mM in DMF) with TCEP (4 µL of a 25 mM stock solution in 1 M TEOA buffer at pH = 7.0) for 15 min at 25 °C. In the meantime, the monomer **3** (8.3 mg, 20 µmol) was dissolved in DMF (16 µL), followed by 1 M TEOA buffer (pH = 7.0, 76 µL). The pH of the resulting mixture was adjusted to 7.0, if necessary. To the solution of **3** was added the freshly prepared solution of initiator **4**, **5** or **26** (8 µL of 25 mM). The polymerization mixture was agitated for 30 min at 25 °C and subsequently quenched with **6** (25 µL of a 0.8 M stock solution in $H_2O$). The quenched mixture was stirred for another 15 min, filtered through a filter pipette tip, and then purified by SEC (under the same conditions as for the analysis) according to the absorbance at 255 nm. The collected polymers were lyophilized. The resulting powders were dissolved in 500 µL $H_2O$ and desalted using a Sephadex (PD-MiniTrap G25) column. The obtained solution was lyophilized and dissolved in the desired amount of $H_2O$ to prepare the stock solution. The obtained polymer **1**, **2** or **25** was characterized using SEC. The concentration of the monomer unit ($c_M$) was determined by the

absorbance at 220 nm ($\varepsilon_{220}$ = 3.6 $10^3$ $M^{-1}\cdot cm^{-1}$). The concentration of polymer ($c_P = c_M / n_M$) was estimated from $c_M$ and the number of monomer units/polymer ($n_M$). In the experimental procedures, CPD concentration refers to the polymer concentration ($c_P$), unless otherwise stated.

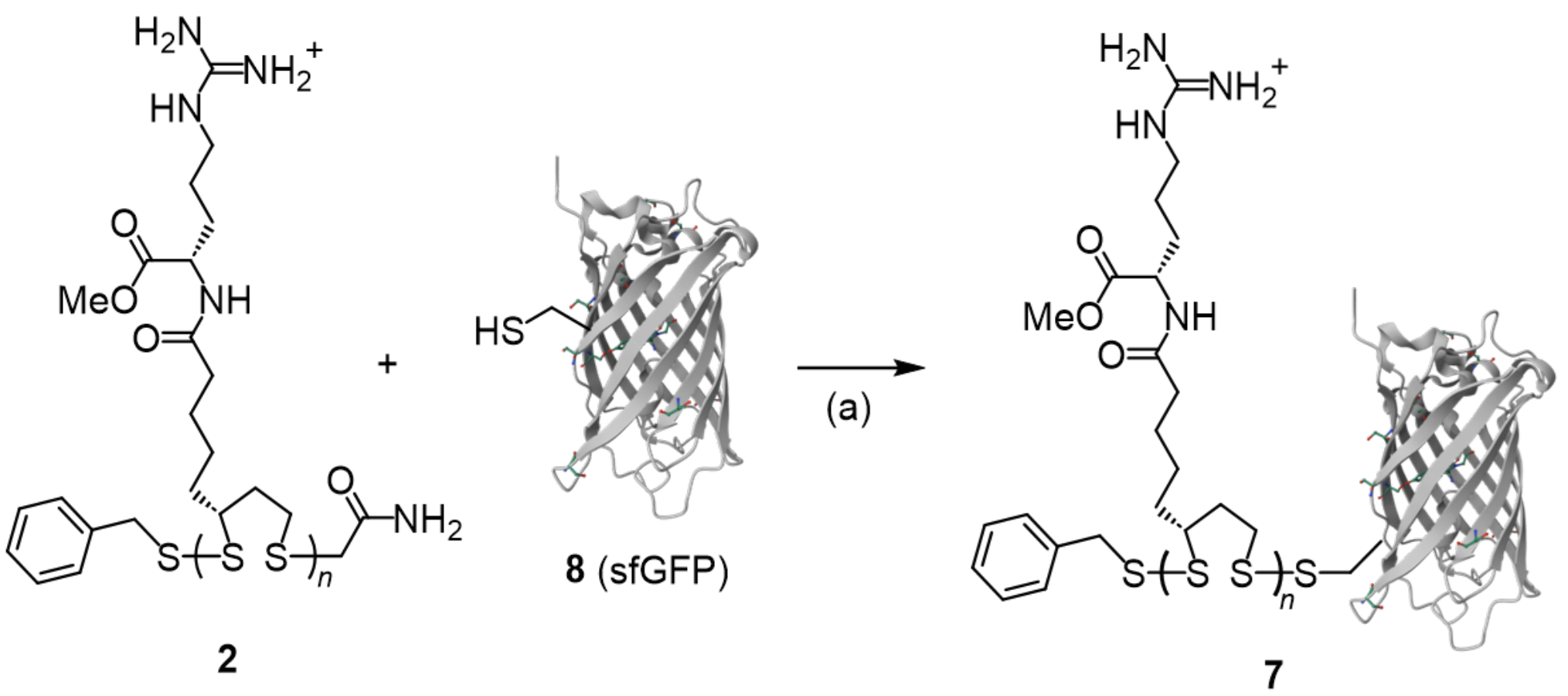


**Scheme S6** | (a) DPBS, 25 °C, 1 h.

*Polymer conjugation through "Grafting-to".* The stock solution of sfGFP **8** was diluted with DPBS to the desired concentration (310 µM), and Bn-CPD$_{55}$ **2** ($c_P$ = 564 µM, $c_M$ = 31 mM) were added. The reaction mixture was shaken at 25 °C for 1 h and then diluted in L15 to give a final concentration of **7** at 75 nM. The bioconjugate **7** was used right after preparation and each time freshly made.

## 5. CPD Characterization

*UV-vis absorption spectroscopy.* The concentrations of the purified CPDs were determined using Beer-Lambert law and the extinction coefficient of either the initiator (CPDs **1** and **25** with $\varepsilon_{FITC}$ = 73 000 $M^{-1}cm^{-1}$ at 498 nm, $\varepsilon_{PC}$ = 5300 $M^{-1}cm^{-1}$ at 450 nm, respectively) [S8] or the disulfide backbone (CPDs **2**, $\varepsilon_{S\text{-}S}$ = 3600 $M^{-1}cm^{-1}$ at 220 nm) [S7]. Briefly, purified CPDs stock solutions were diluted 1:100 and 1:200 in DPBS and transferred to a microcuvette (path length, $l$ = 0.3 cm). Absorbance spectra were then recorded from 200 – 600 nm. All measurements were performed in duplicates. Representative UV/vis spectra are shown in Figure S3.

*Quenching Factor.* Following the procedure described in reference [S1]. Briefly, a stock solution of Fl-CPD **1** (200 µM, $H_2O$) was diluted to give 1 µM solution in PBS buffer. The fluorescence emission spectrum was then recorded from 498 nm to 600 nm upon excitation at 488 nm (slits 5 nm). The emission spectrum of **1** under reducing conditions was also recorded after treating the solution of **1** (10 µL, 200 µM in PBS buffer) with DTT (10 µL, 2 mM in PBS buffer) for 30 min before diluting to 1 µM in PBS buffer. Comparison of the obtained data to those of Fl-AspA **10** demonstrated that their fluorescence intensities are comparable in both oxidized (Figure S3d) and reduced forms (Figure S3e).

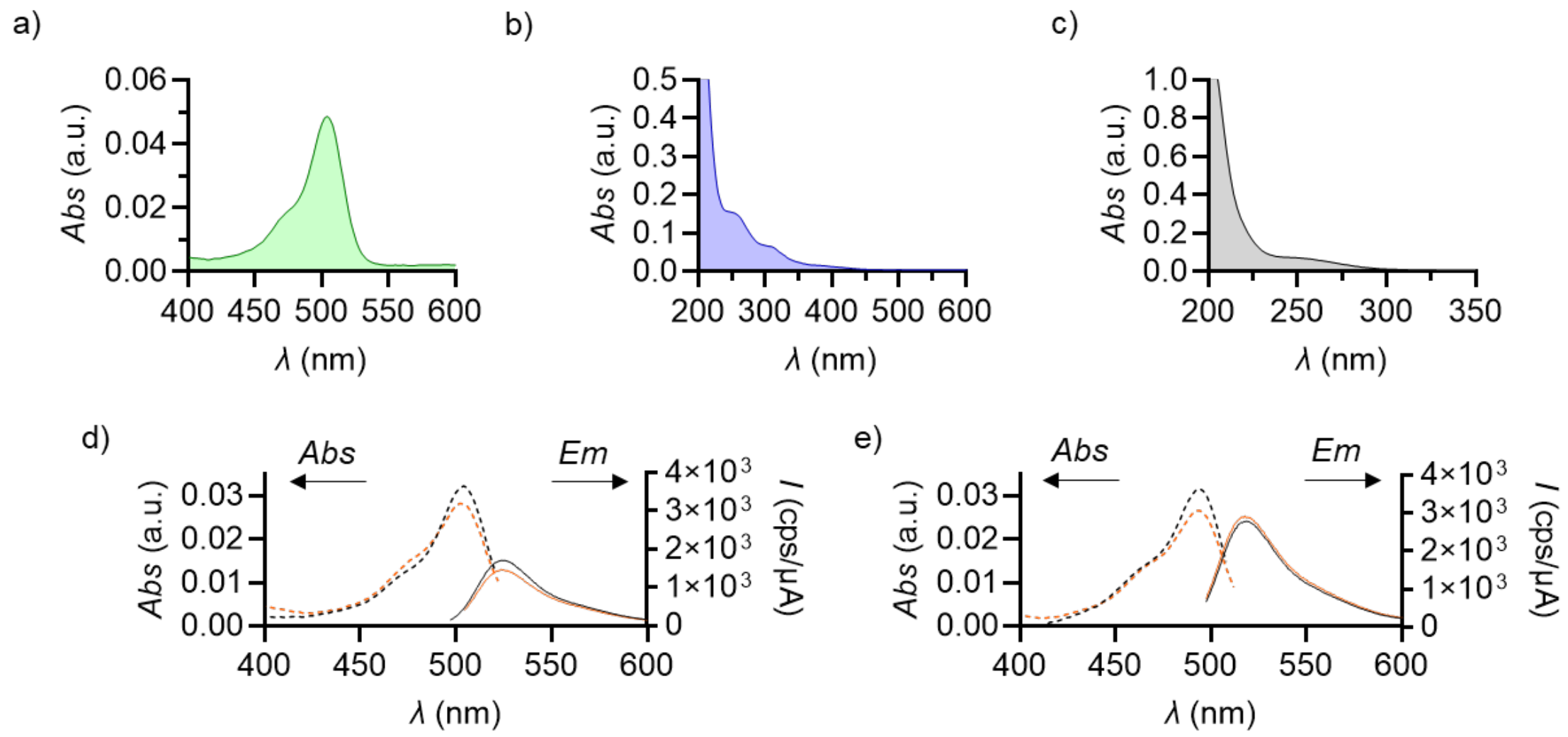


**Figure S3** | UV/vis traces of CPDs: a) Fl-CPD **1**, b) PC-CPD **25** and c) Bn-CPD **2**. d) Absorption (dashed) and emission (solid) spectra of **10** (black) and **1** (orange) (1 µM, PBS) and (e) after reacting with DTT for 30 min at 25 °C. $l_{ex}$ at 488 nm. Slits 5 nm; $l$ = 1 cm.

*SEC.* The purified CPDs stock solutions were diluted with $H_2O$ (30 µM, 100 µL) and analyzed by SEC, using Superdex 75 10/300 GL with 0.1 M sodium acetate buffer in 3:7 $CH_3CN/H_2O$ (pH = 6.5) as an eluent at 0.4 mL/min (from 0 to 40 min), then 0.6 mL/min (from 41 to 120 min). The chromatograms obtained at 255 nm were used for characterization in comparison with those of molecular weight standards.

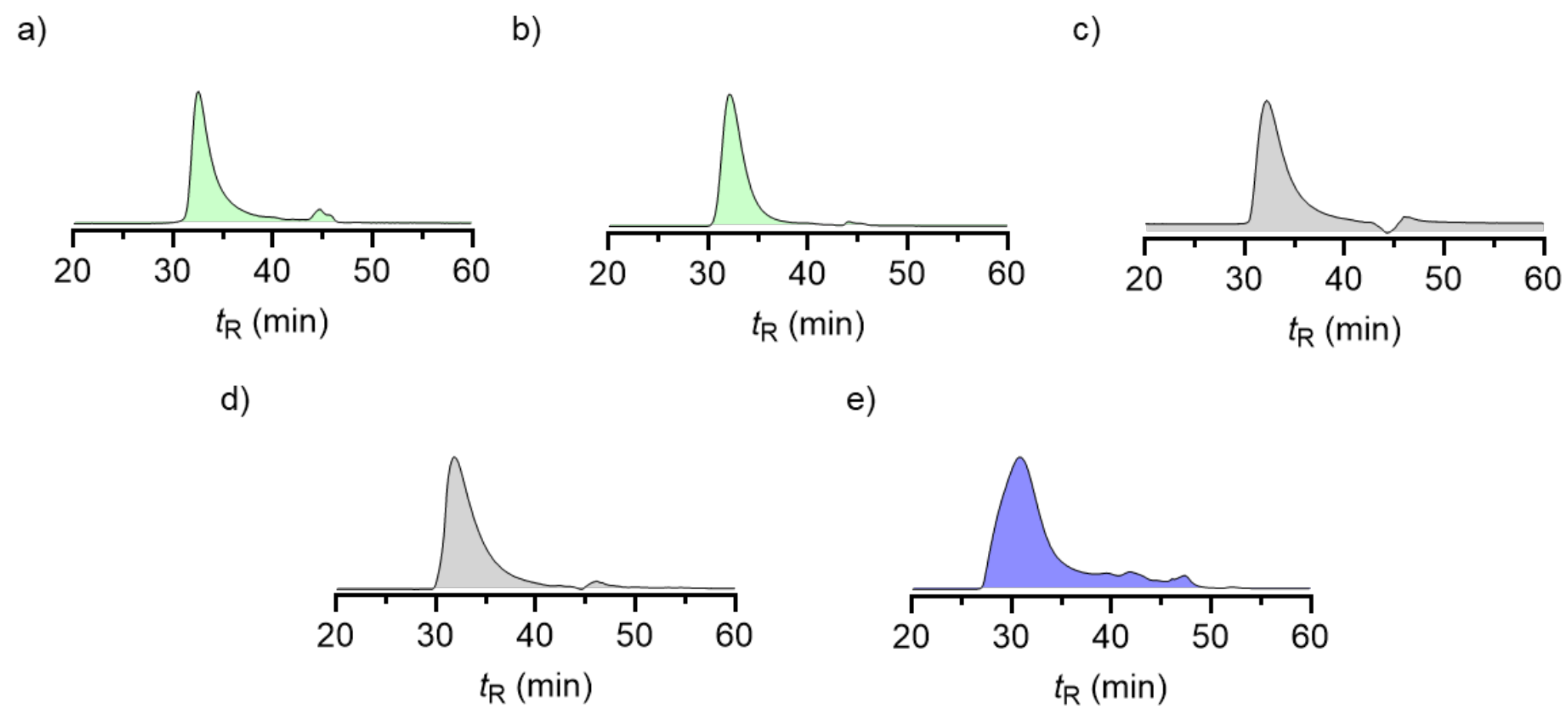


**Figure S4** | SEC traces after purification (255 nm) of CPDs: a) Fl-$CPD_{34}$ **1**, b) Fl-$CPD_{55}$ **1**, c) Bn-$CPD_{38}$ **2**, d) Bn-$CPD_{55}$ **2**, and e) PC-$CPD_{93}$ **25**.

**Table S1** | Characteristics of CPDs obtained through "grafting-from" determined by SEC.

| Entry | Polymer | *Đ*[a] | $M_n$ [kDa][b] | $n_M$[c] |
|---|---|---|---|---|
| 1 | Fl-$CPD_{34}$ **1** | 1.1 | 16 | 34 |
| 2 | Fl-$CPD_{55}$ **1** | 1.1 | 25 | 55 |
| 3 | Bn-$CPD_{38}$ **2** | 1.3 | 16 | 38 |
| 4 | Bn-$CPD_{55}$ **2** | 1.3 | 24 | 55 |
| 5 | PC-$CPD_{93}$ **25** | 1.5 | 41 | 93 |

[a]Dispersity. [b]Number average molecular weight. [c]Theoretical average number of monomers in polymer, calculated as $n_M = (M_n - MW[I] - MW[acetamide]) / MW[3]$, in which I corresponds to **4**, **5**, or **26**.

## 6. CPD Stability in Leibovitz L15 Medium

The stability of Fl-$CPD_{34}$ **1** (4 µM) in Leibovitz's L15 Medium was evaluated by SEC at different time points. The polymer was detected through the fluorescence of fluorescein ($\lambda_{ex}$ = 490 nm, $\lambda_{em}$ = 520 nm), which showed no obvious change during 12 h.

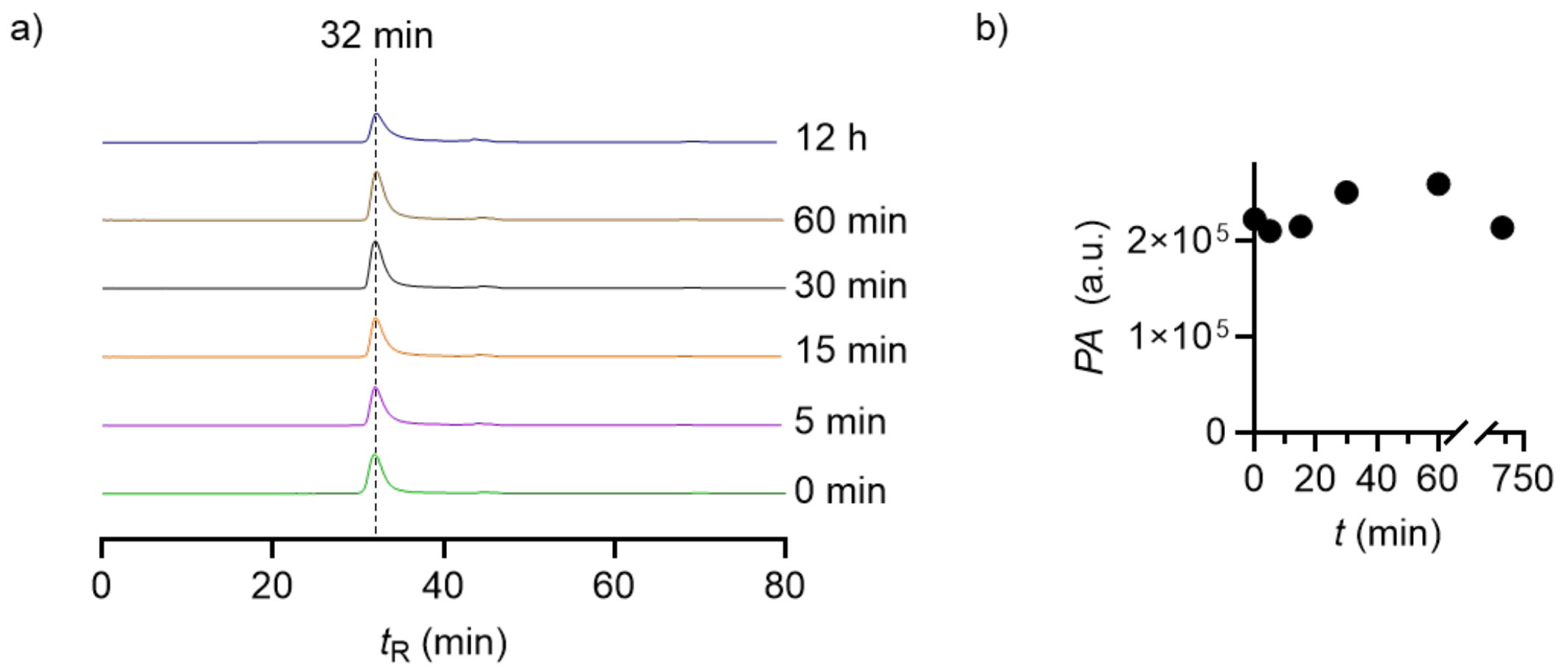


**Figure S5** | Stability of Fl-CPD **1** (4 µM) in L15 medium monitored by SEC. (a) SEC traces of polymer **1** over time, and b) the corresponding peak areas (PA) of the peaks at 32 min.

## 7. Cell Culture

Human cervical cancer-derived HeLa Kyoto (HK) cells were cultured in complete DMEM (GlutaMAX, 4.5 g/L D-glucose), which contains 10% fetal calf serum (FCS) and 1% Penicillin/Streptomycin (PS). The cells were grown under 5% $CO_2$ humidified atmosphere at 37 °C on a 75 $cm^3$ tissue culture flask (TPD Corporation). Cells were harvested by treatment with 3 mL of phenol-red free TrypLE Express, followed by the addition of 10 mL of complete FDMEM (GlutaMAX, 4.5 g/L D-glucose) at 37 °C. The cells were spun down at 1500 g for 3 min, re-suspended in complete FDMEM (GlutaMAX, 4.5 g/L D-glucose), and plated according to the concentration needed.

## 8. Lysate Preparation

Cell lysates were prepared by treating the cells ($10^6$/well) in a 6 well-plate for 15 min at 4 °C with a RIPA lysis buffer (1X, Millipore, 20-188) supplemented with cOmplete™ EDTA-free protease inhibitor cocktail (Sigma-Aldrich 4693159001) and PMSF (1 mM, Sigma-Aldrich 52332). The lysate was collected by scraping the surface of the well. After centrifugation at 14.5 x 1000 g for 15 min at 4 °C, the supernatant was collected. The protein concentration was determined using the BCA assay (Thermo Scientifics 23225) and adjusted with a solution of RIPA 1X to a final concentration of either 500 µg/mL or 1 mg/mL. The protein lysate was either used immediately, frozen at -20 °C for experiments within the next few days, or stored at -80 °C for experiments in the following weeks.

## 9. Western Blot Analysis

The protein lysate (50 µL, 1 mg/mL) was pre-mixed with a reducing loading buffer (Laemmli buffer 4x, Bio-Rad 1610747) for 10 min at 95 °C. The lysate samples (20–30 µg per well) were loaded onto a precast PAGE gel (4-20% gradient, Bio-Rad 4561096), and the proteins were separated by electrophoresis at 200 V for 1 h. The proteins were transferred to a PVDF membrane using the iBlot 3 Western Blot Transfer System (Thermo Fisher Scientific). The proteins were first revealed using a

ponceau S solution for 15 min at RT. Then, the membrane was blocked by incubating with a solution of 1% BSA in TBST for 20 min at RT. Streptavidin Alexa Fluor 670 conjugate (10 mL, 4 µg/mL) in TBST was incubated with the membrane at 4 °C overnight. The membrane was washed 3 times with TBST buffer (5 min each time) and then fluorescence was visualized with a Fusion Solo (Vilber Lourmat) using the Cy-5 channel.

## 10. Cellular Uptake

### 10.1. Confocal Laser Scanning Microscopy

HK cells were seeded at 8.0 × $10^4$ cells/mL on a 35 mm glass-bottom dish and cultured in complete FDMEM overnight at 37 °C under 5% $CO_2$. After removing the medium, cells were rinsed with L15 (3 × 1 mL) and treated with the desired fluorescent compound in L15. The cells were incubated at 37 °C under 5% $CO_2$ atmosphere for indicated incubation time. Then, the medium was removed by aspiration, and cells were washed with L15 supplemented with 0.1 mg/mL heparin sulfate (3 × 1 mL), L15 (3 × 1 mL) and kept in L15 medium supplemented with plasma membrane staining dye WGA CF640R (5 µg/mL, Biotinium 29026-1) for imaging. The distribution of fluorescent compounds was analyzed without fixing by CLSM (Leica SP8), using either a 20× WI or 63× OI objective lens. The cellular distribution of fluorescein (Fl) and GFP was imaged using a 488 nm laser at fixed powers of 2% and 10%, respectively (unless otherwise specified). Emission was collected between 500 and 550 nm. For photocatalysts (PCs) **25-26**, the images were recorded using a 405 nm laser at 30% power (unless otherwise specified), and the resulting emission signal was collected between 500 and 550 nm. The cellular distribution of WGA-CF640R and SAV680 was imaged using a 647 nm laser at a fixed power of 0.5% and 1%, respectively, with emission collected between 655 and 750 nm.

## 10.2. Data Analysis

The resulting images were analyzed using Fiji software. Fluorescence intensity was quantified from the selected green channel by manually selecting the cytoplasm excluding the membrane, and calculating the mean fluorescence intensity per pixel.

For experiments involving WGA CF640R (CPP activators and cellular uptake in knocked-down cells, sections 12 and 17, respectively), an additional mask was generated from the red channel to delineate the cell membrane (Figure S6c). This membrane mask was then applied to the green channel (Figure S6d) to facilitate the quantification only of the intracellular fluorescence signal.

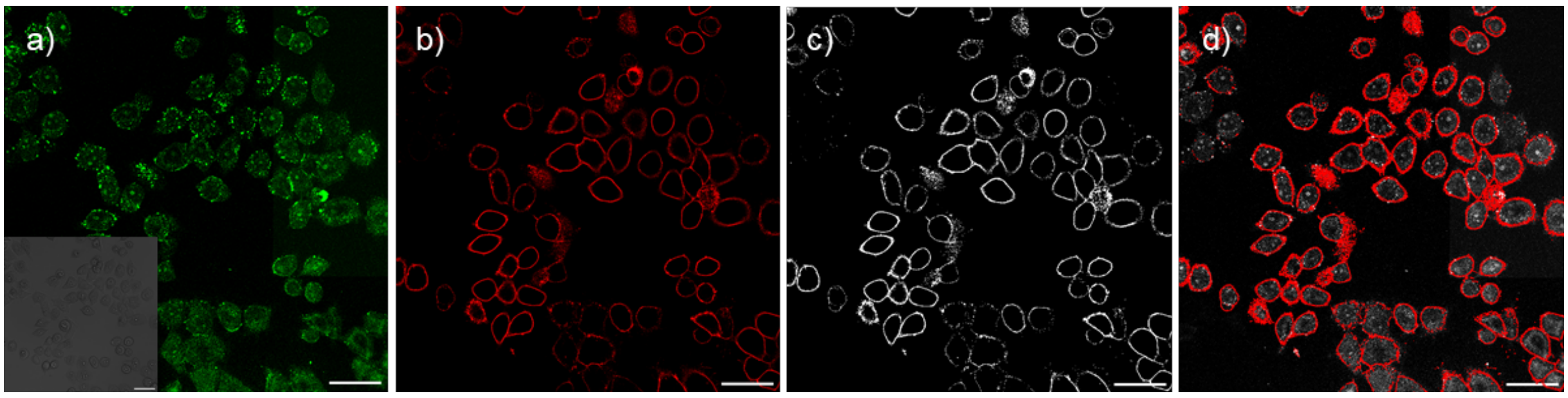


**Figure S6** | a) Representative CLSM images of the green channel (CPDs), b) the red channel (WGA CF640R), c) the corresponding membrane mask obtained from the red channel (ROI), and d) applied to the green channel to subtract fluorescent signal from membrane, scale bars = 50 µm, inset: brightfield.

### 10.3. Optimization of Cellular Uptake

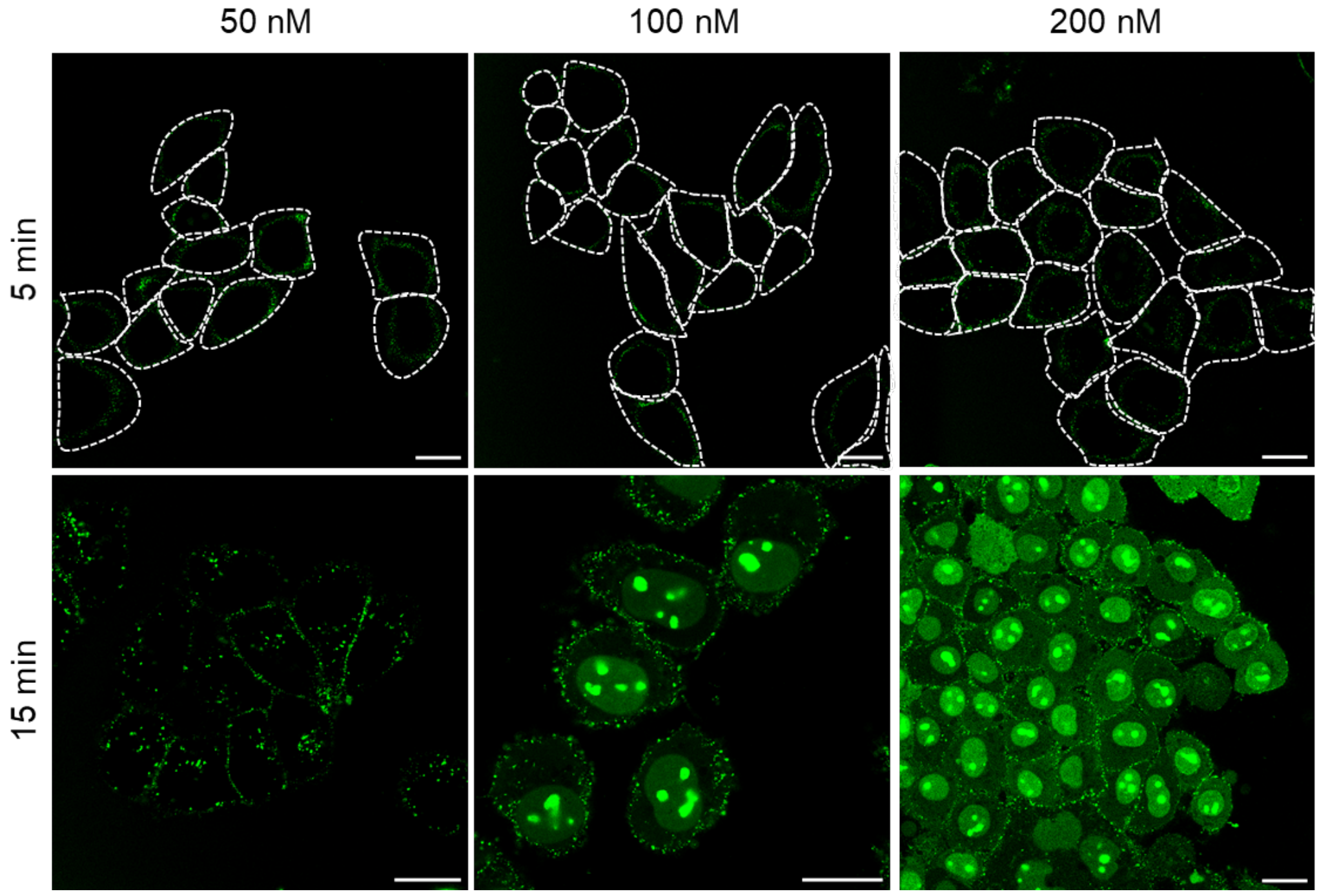


**Figure S7** | CLSM images of HK cells treated with Fl-CPD$_{34}$ **1** (green) after 5 or 15 min incubation with either 50, 100 or 200 nM. For 5 min, the cells were delimited using the brightfield images, laser power = 3%, scale bars = 20 µm. *Note:* cellular uptake is not linearly proportional to [**1**], rather, uptake occurs only after a threshold concentration is reached.

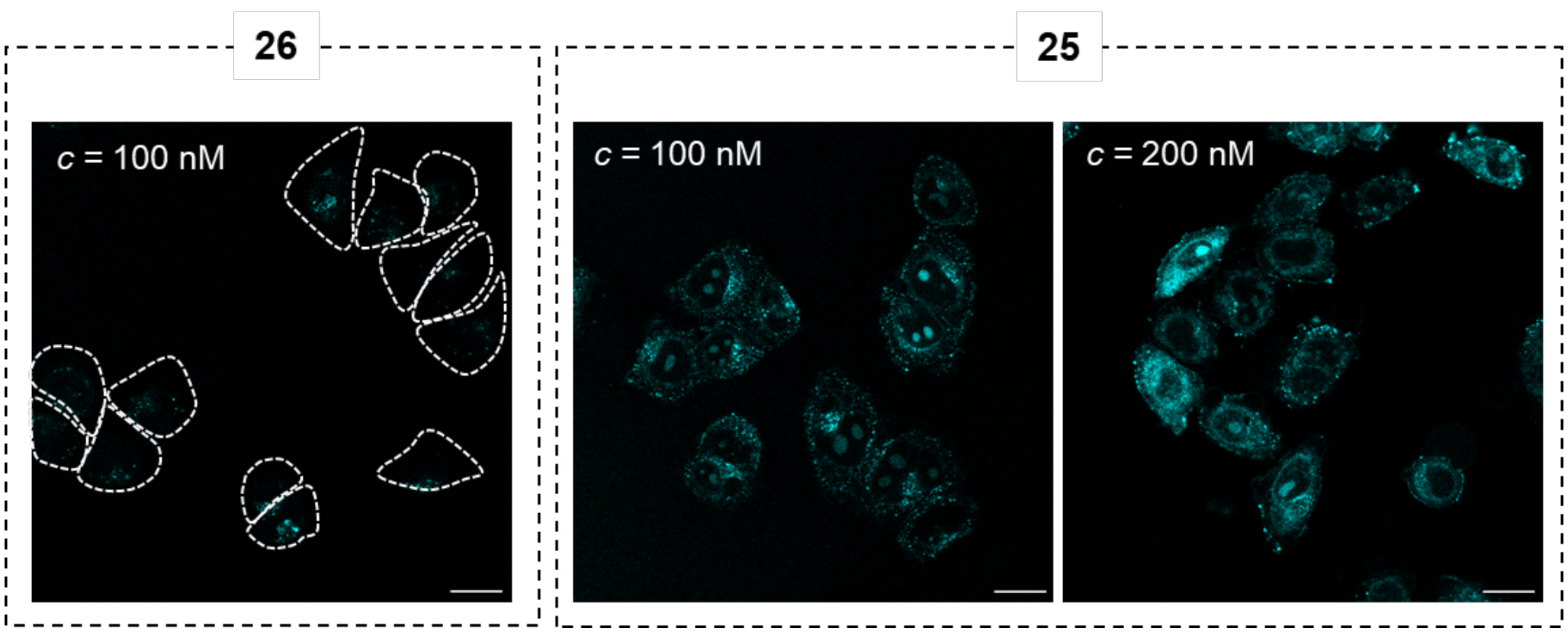


**Figure S8** | CLSM images of HK cells treated with either PC-Cys **26** or PC-CPD$_{93}$ **25** (cyan) after 30 min incubation with either 100 or 200 nM. For **26**, the cells were delimited using the brightfield images, laser power = 30%, scale bars = 20 µm.

### 10.4. CPD Extraction with Heparin

To a solution of **10** in L15 (3 µM, 100 µL), an equimolar amount of TCEP was added and the reaction was shaken for 5 min at RT. Afterward, Bn-$CPD_{55}$ **2** was added (0.12 µL of a $c_P$ = 60 µM stock solution in $H_2O$) and the bioconjugation reaction mixture was shaken at 25 °C for 5 min. Then, the mixture was diluted with 200 µL of L15 (final [**10**] = 1 µM) and added to cells. After incubation at 37 °C under 5% $CO_2$ atmosphere for 30 min, the cells were rinsed either with i) fresh L15 (3x 0.8 mL, Figure S9a), or ii) a heparin solution 0.1 mg/mL in L15 (3x 0.8 mL, Figure S9b), and then kept in fresh L15 for imaging. Images show effective removal of a fraction of the polymers associated with the outer cell membrane by washing with a heparin solution (Figure S9a *vs* S9b).

A control experiment was performed using the reduced **10** without CPDs, which gave much weaker fluorescence in cells (Figure S9c).[S7]

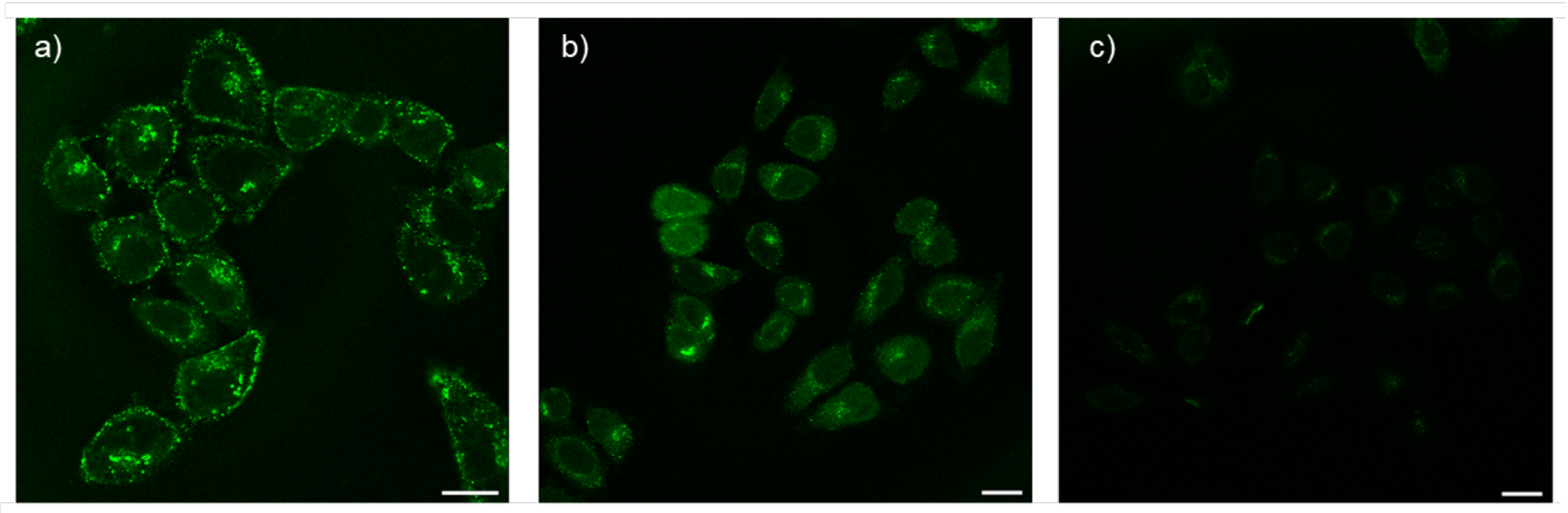


**Figure S9** | CLSM images of HK cells incubated with **10** ($c$ = 1 µM in L15, 30 min, 37 °C) without (c) and with (a, b) preceding bioconjugation with CPDs, (a) without and (b) with rinsing with heparin before imaging. Intensities are comparable, scale bars = 20 µm.

## 11. Inhibition of CPDs

### 11.1. TMU Inhibitors

Before performing the general cellular uptake assay 10.1., cells were incubated for 1 h at 37 °C with the corresponding TMU inhibitor at 5 or 20 µM, or at concentrations above the $IC_{50}$ values

determined for other CAXs in reference [S1]. The fluorescence distribution was quantified using the 20× WI objective lens, covering 50–100 cells per image, with at least 2 replicates per inhibitor. The results and a representative image of each condition are reported in Figure S10-Figure S11.

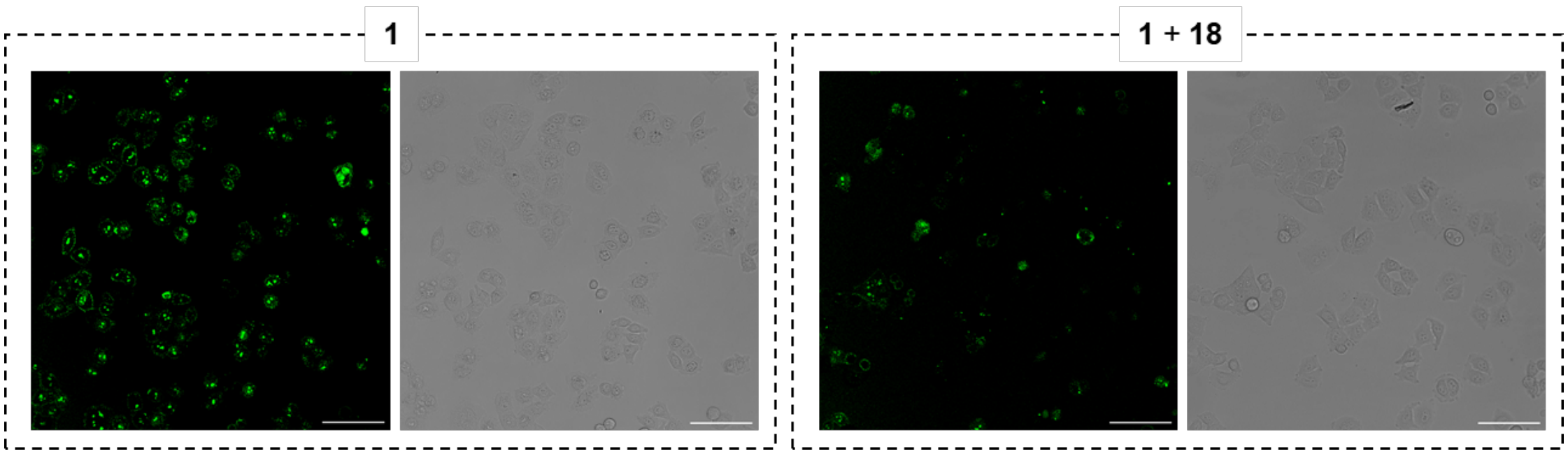


**Figure S10** | CLSM images of HK cells treated with **1** (75 nM, 20 min) in absence (left) or presence (right) of **18** (20 µM, 1 h). The images are representative of the inhibitor screening using a 20× objective lens covering a larger number of cells, laser power = 2%, scale bars = 100 µm. Replicates were performed per condition.

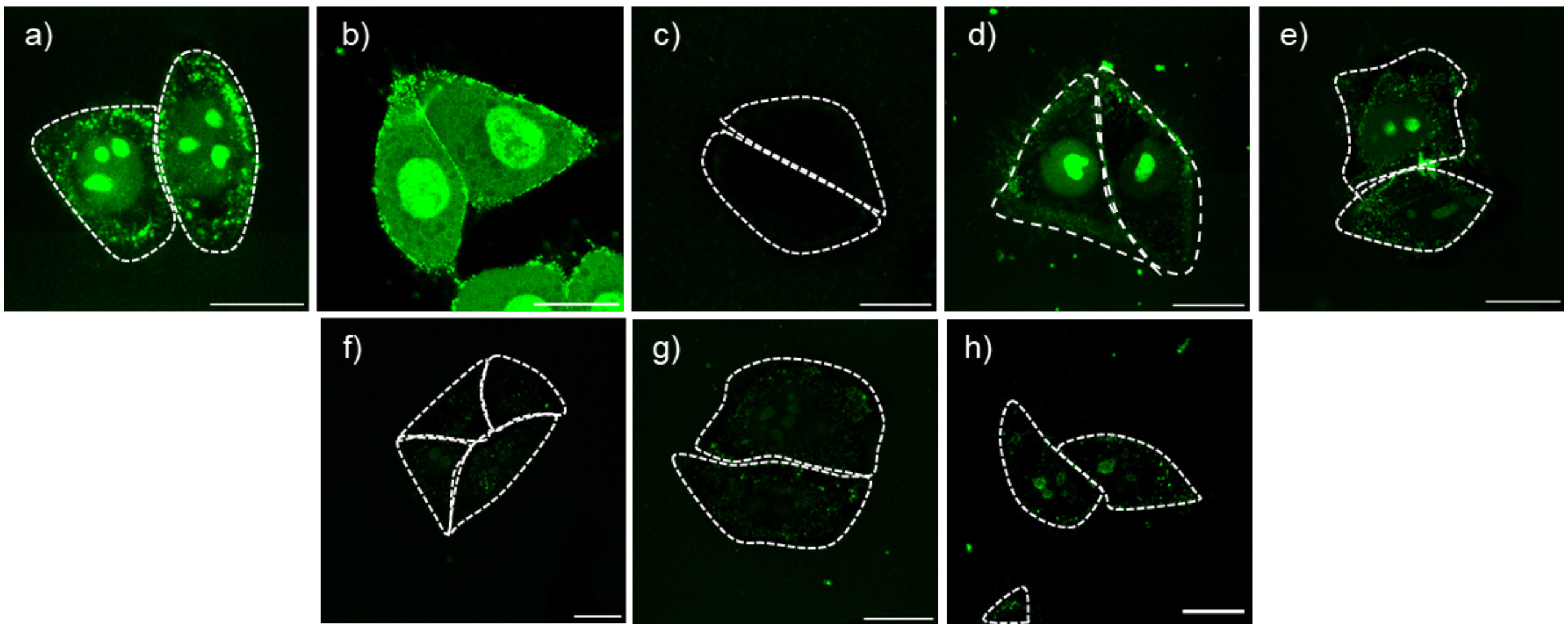


**Figure S11** | Representative CLSM images of HK cells treated with **1** (75 nM, 20 min) in a) absence or presence of either b) $CPD_{38}$ **2** (75 nM, 15 min), c) **15** (500 µM, 1 h), d) **16** (5 µM, 1 h), e) **17** (5 µM, 1 h), f) **18** (5 µM, 1 h), g) **19** (50 µM, 1 h) and h) **28** (10 µM, 1 h), laser power = 2%, scale bars = 20 µm.

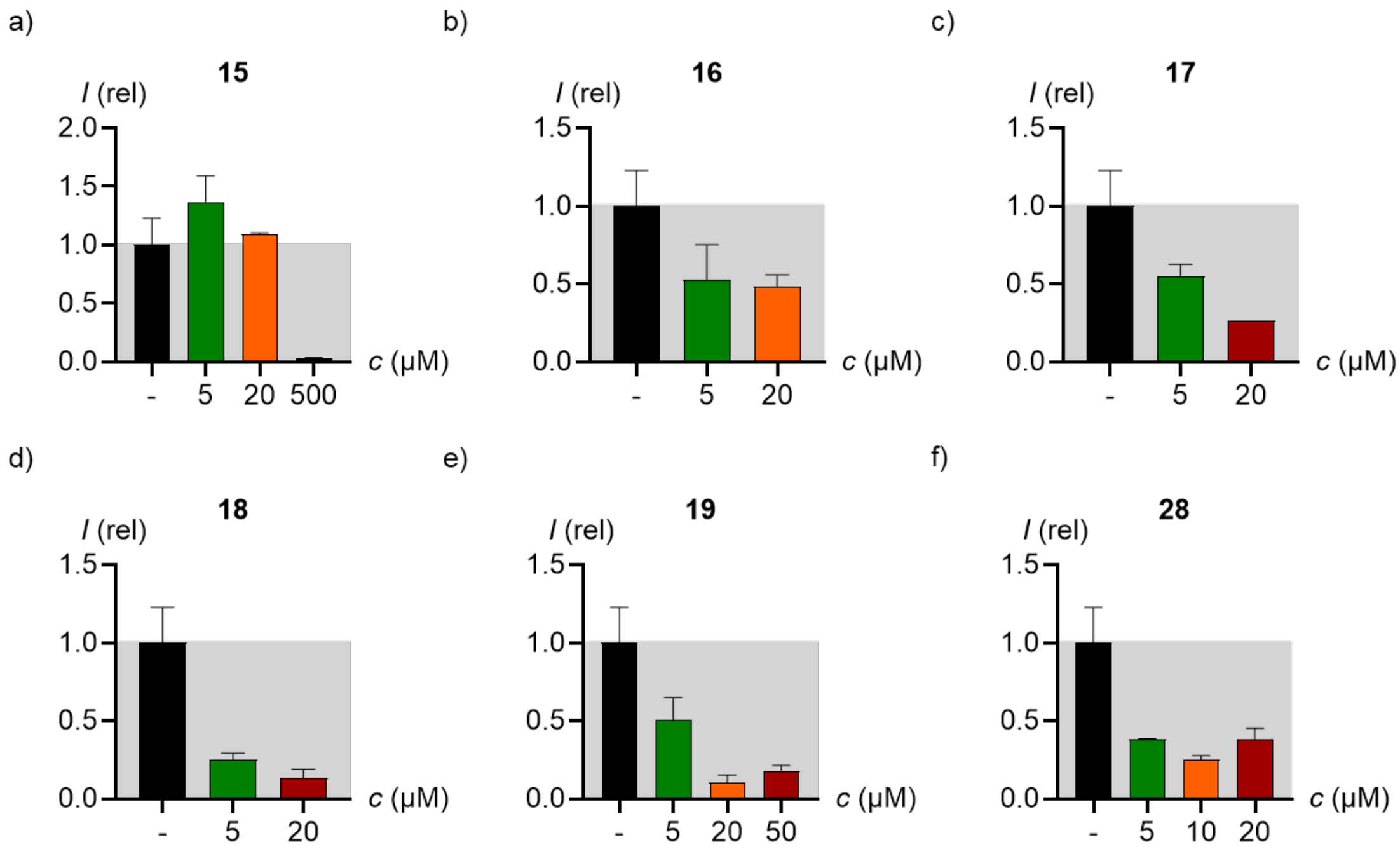


**Figure S12** | Relative fluorescence intensity *I* (rel) ± SD (n = 3) of **1** (75 nM) in HK cells as a function of the concentration of a) **15** (DTNB), b) **16** (AspA), c) **17** (ETP), d) **18** (BPS), e) **19** (dMAC), and f) **28** (AspAmine).

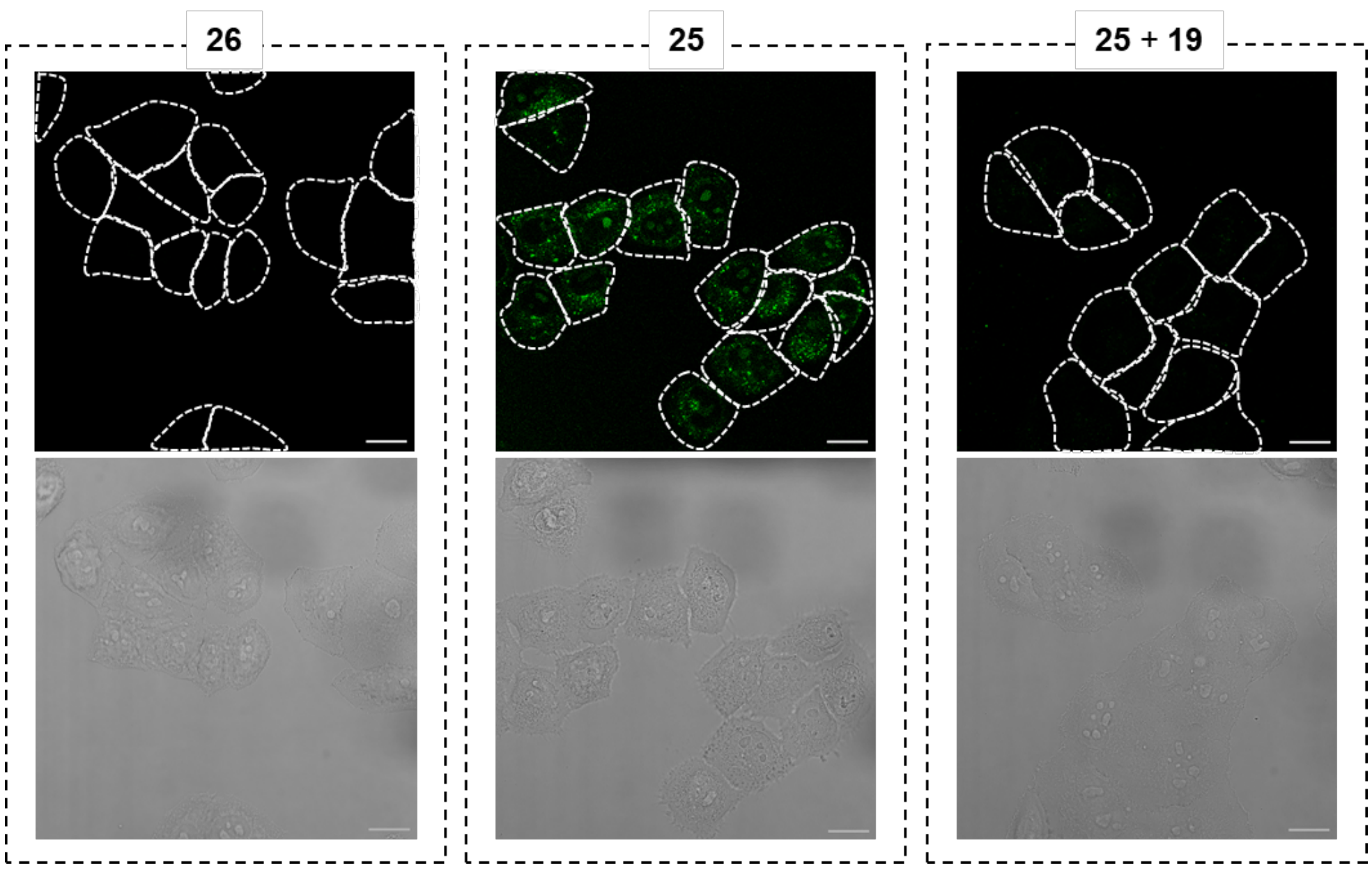


**Figure S13** | Representative CLSM images of HK cells treated with PC-Cys **26** (100 nM, 30 min) (left), PC-CPD$_{93}$ **25** (100 nM, 30 min) (middle) and PC-CPD$_{93}$ **25** (100 nM, 30 min) co-incubated with **19** (dMAC, 50 µM, 1 h), laser power = 30%, scale bars = 20 µm.

### 11.2. PDI Inhibitors

Before performing the general cellular uptake assay 10.1., cells were incubated for 1 h at 37 °C with the corresponding PDI inhibitor, *i.e.* **20** (LOC14) and **29** (16F16), at previously determined $IC_{50}$ values [S1]. The fluorescence distribution was quantified using the 20× WI objective lens covering 50–100 cells per image, with at least 2 replicates per inhibitor. The results are reported in Figure S14.

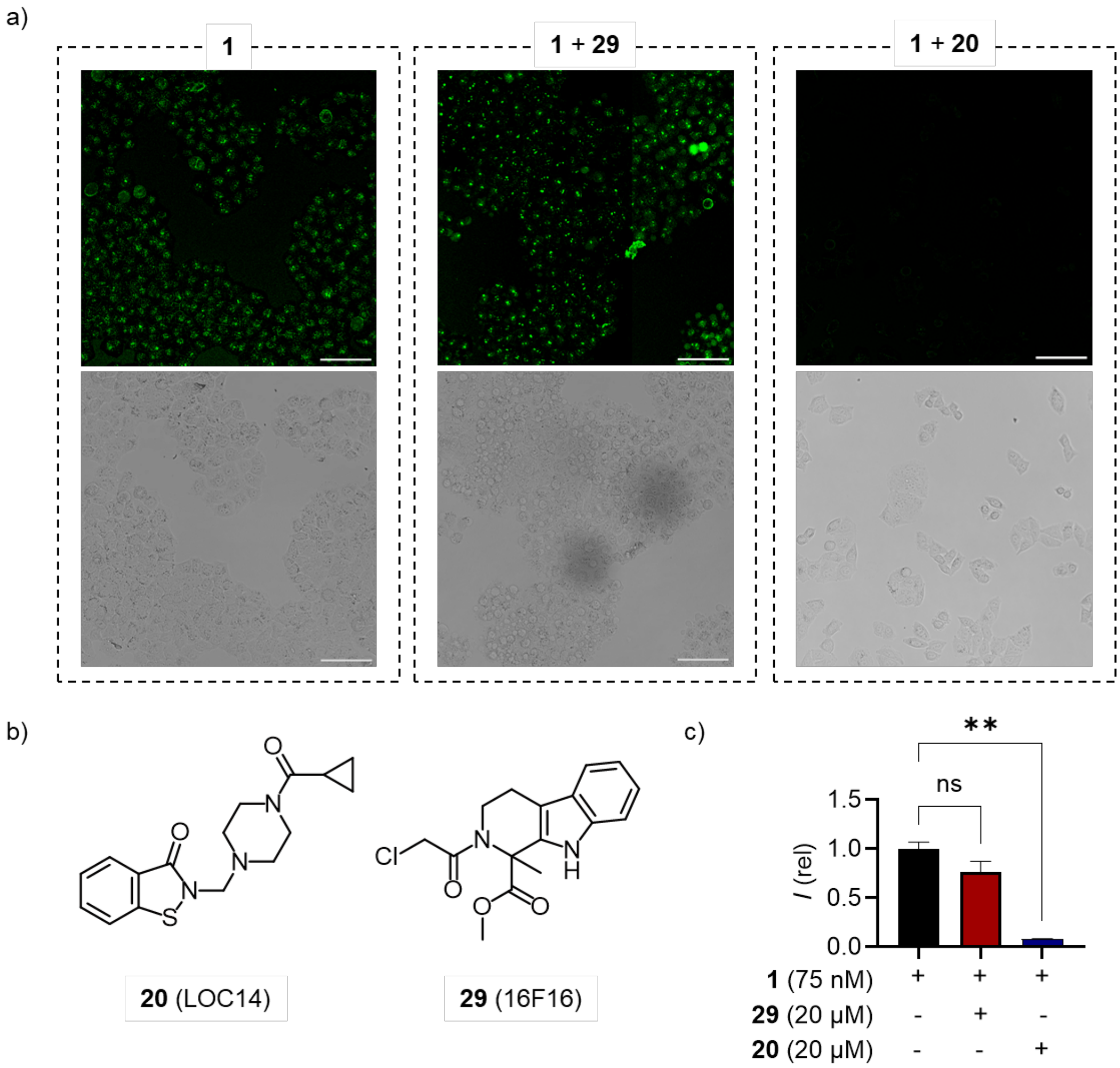


**Figure S14** | a) Representative CLSM images of HK cells treated with **1** (75 nM, 20 min) in absence (left) or presence of either **29** (16F16, 20 µM, 1 h) (middle) or **20** (LOC14, 20 µM, 1 h) (right), b) their respective structure and c) the relative fluorescence intensity of **1** at $IC_{50}$ values of those PDI inhibitors, laser power = 3%, scale bars = 100 µm ($I$ ± SD, n = 3, $P$ = 0.0048 obtained with a non-parametric one-way ANOVA test).

Preliminary data related to this chapter, obtained under different conditions, were reported in the following PhD thesis: M. Cognet, Université de Genève, 2025.

## 12. CPP Activators

Before performing the general cellular uptake assay 10.1., cells were incubated for 5 min at 37 °C with the corresponding CPP activators, pyrenebutyrate **21** (50 µM), boron clusters **22** (10 µM), amphiphilic calixarene **23** (10 µM) and fluorophile **24** (10 µM). The corresponding transporter **1** or **7** (75 nM) was added and incubated at 37 °C for additional 20 min for **1** and 30 min for **7**. The fluorescence distribution was quantified using the 20× WI objective lens, covering ~50–100 cells per image, with at least 2 replicates per inhibitor. The representative images are in Figure S15-S16.

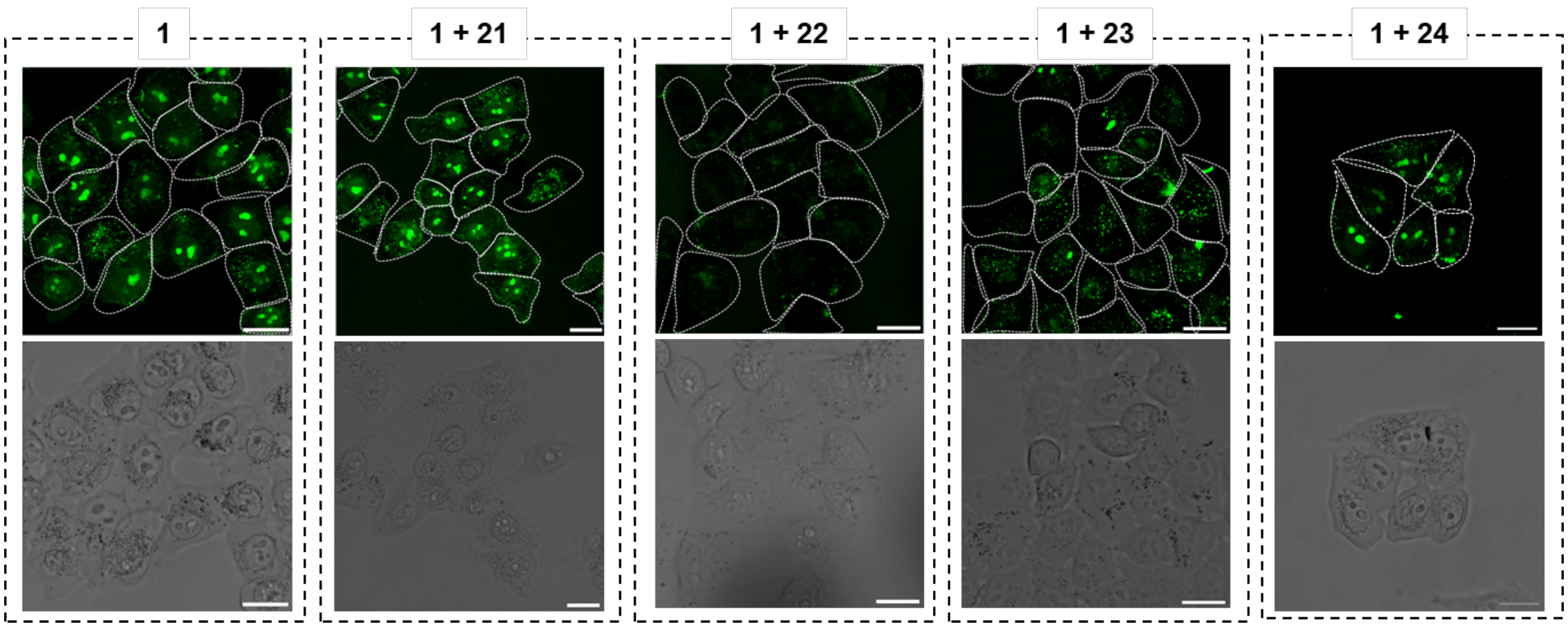


**Figure S15** | Representative CLSM images of HK cells treated with **1** (75 nM, 20 min) in absence or presence of either **21** (50 µM, 5 min), **22** (10 µM, 5 min), **23** (10 µM, 5 min) or **24** (10 µM, 5 min), laser power = 3%, scale bars = 20 µm.

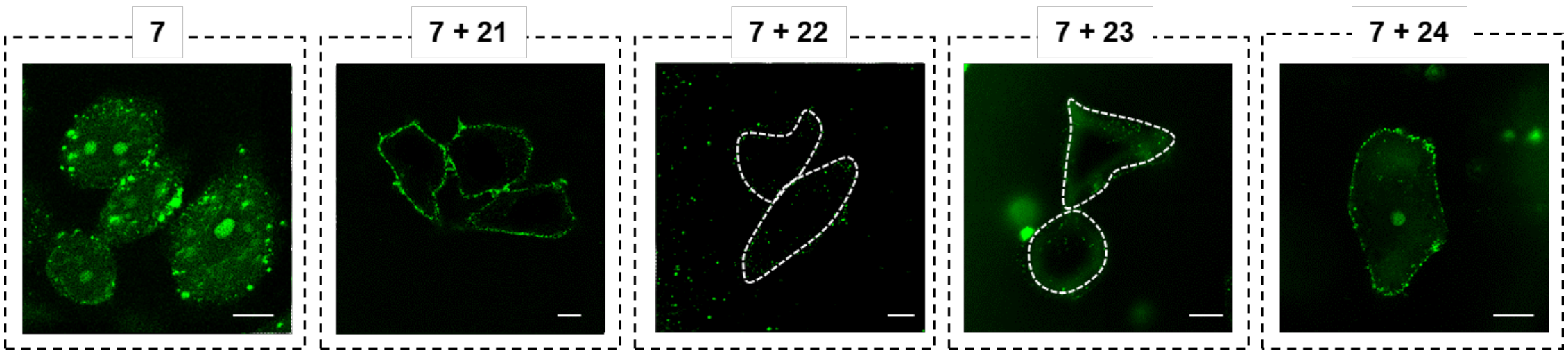


**Figure S16** | Representative CLSM images of HK cells treated with **7** (75 nM, 30 min) in absence or presence of either **21** (50 µM, 5 min), **22** (10 µM, 5 min), **23** (10 µM, 5 min) or **24** (10 µM, 5 min), laser power = 2%, scale bars = 10 µm.

## 13. CPD Depolymerization

### 13.1, CPD Depolymerization in Condensates

*Fluorescence spectroscopy.* Fluorescence excitation ($l_{em}$ = 520 nm) and emission ($l_{ex}$ = 480 nm) spectra were recorded as a function of time (5 min intervals after GSH addition) at RT, for solutions of a) Fl-CPD$_{34}$ **1** (0.2 µM, $c_M$ = 6.6 µM) in DPBS (100 µL), followed by the addition of polyU (2 × 0.5 µL of $c_M$ = 0.66 mM in DPBS), and then GSH (1 µL of 1 M in $H_2O$), b) Fl-CPD$_{34}$ **1** (0.2 µM, $c_M$ = 6.6 µM) in DPBS (100 µL), followed by the addition of GSH (1 µL of 1 M in $H_2O$), then polyU (2 × 0.5 µL of $c_M$ = 0.66 mM in DPBS), and c) **35** (0.1 µM) in DPBS (100 µL), followed by the addition of GSH (1 µL of 1 M in $H_2O$), then polyU (0.5 µL of $c_M$ = 0.66 mM in DPBS). GSH stock solution (1 M) was prepared by dissolving commercial neutral GSH in reduced form (1.4 mg, 4.5 µmol) in aqueous NaOH (4.5 µL of 1 M).

The excitation and emission maxima of fluorescein in Fl-CPDs **1** are red-shifted compared to those of the monomer, likely due to differences in the local hydrogen-bonding environment of the dye [S12]. Thus, the reductive depolymerization of Fl-CPDs **1** can be conveniently monitored as a shift in excitation maxima (Figure S17, 3i). The addition of polyU to Fl-CPDs **1** increased scattering (Figure S17a, arrow at 485 nm) and reduced fluorescence intensity (green to red) without affecting the excitation/emission maxima. After subsequent addition of GSH, the maxima quickly blue-shifted (Figures 3i, j, S17a, $t_{1/2} \approx$ 5 min) to those of the monomer (Figure S17c), while intensity recovery was

slower. In comparison, the rate of blue shift was only slightly higher when GSH was added before polyU (Figures 3j, S17b, $t_{1/2} \approx 3$ min), and polyU addition did not affect intensity (Figure S17b, blue vs red). These results indicate that GSH reduces Fl-CPDs in condensates unhindered, not surprising given the liquid nature of condensates.

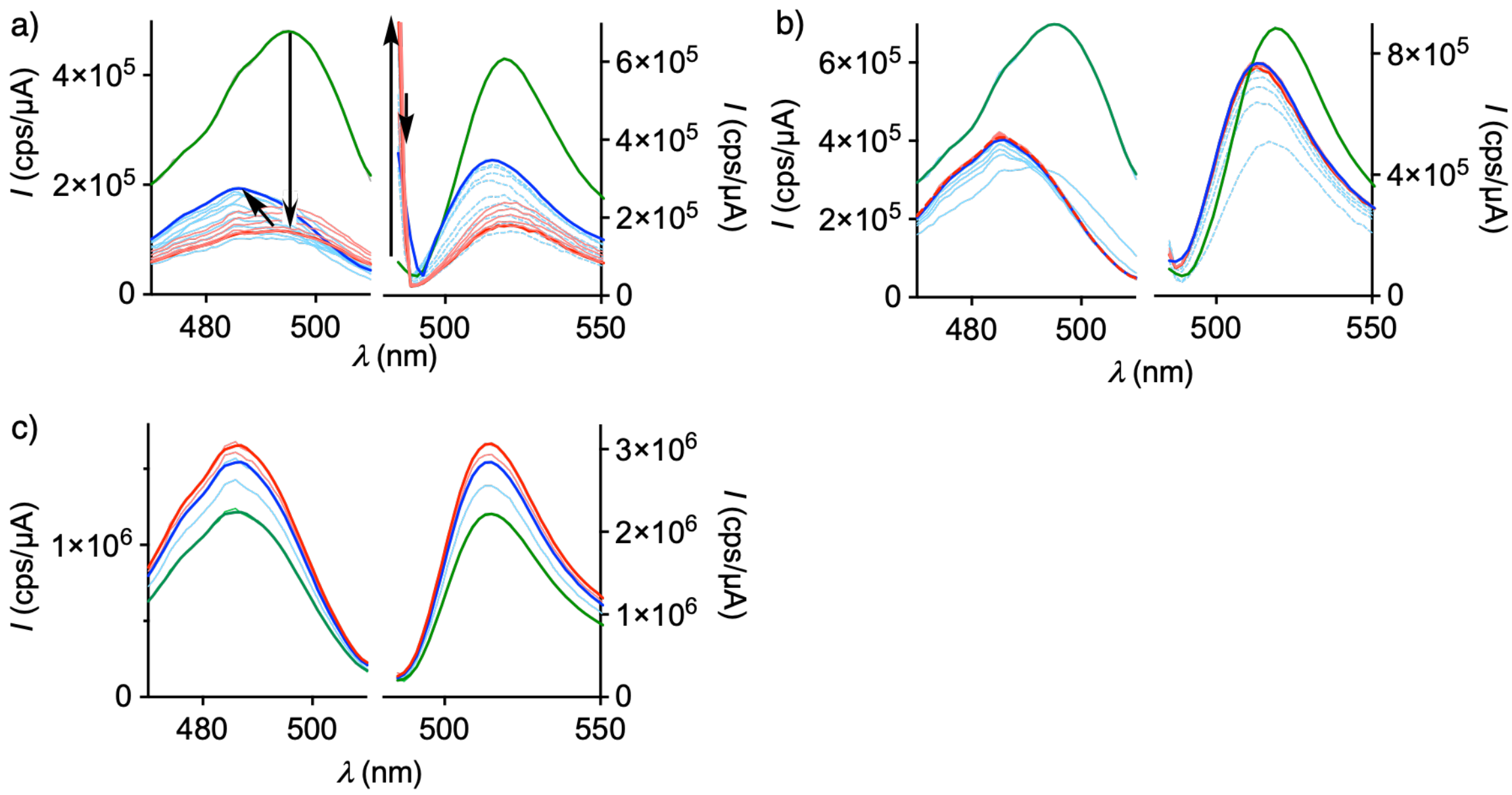


**Figure S17** | Excitation (left, $l_{em}$ = 520 nm) and emission (right, $l_{ex}$ = 480 nm) spectra of (a) Fl-CPD$_{34}$ **1** in DPBS (0.2 µM, $c_M$ = 6.6 µM; green), after the addition of polyU ($c_M$ = 6.6 µM; pale red to red), followed by GSH (10 mM; pale blue to blue), (b) Fl-CPD$_{34}$ **1** in DPBS (0.2 µM; green), after the addition of GSH (10 mM; pale blue to blue), followed by polyU ($c_M$ = 6.6 µM; pale red to red dashed) and (c) $(Fl)_2$ **35** in DPBS (0.1 µM, green), after the addition of GSH (10 mM; pale blue to blue), followed by polyU ($c_M$ = 6.6 µM; pale red to red).

*FLIM.* Condensates were formed within wells of 18-well slide (ibidi) by mixing a) polyU ($c_M$ = 1.2 mM) and Fl-CPD$_{34}$ **1** (0.1 µM, $c_M$ = 3.4 µM) in DPBS (100 µL), or b) polyU ($c_M$ = 2.4 mM), polyR ($c_M$ = 1.0 mM), and Fl-CPD$_{34}$ **1** (0.1 µM, $c_M$ = 3.4 µM) in DPBS (100 µL). After ≈0.5 h of maturation, GSH (1 µL of 1 M) was added. FLIM images were acquired as a function of time at RT

using Leica Stellaris 8 Falcon (63x) with a white laser at 488 nm (20 MHz) and detection at 550-650 nm (Figures S18, S19, and 3d-g).

For comparison, FLIM images were also acquired of Fl-CPD$_{34}$ **1** in HK cells. The HK cells were plated as described in section 7 at 8.0 × 10$^{4}$ cells/mL on a 35 mm glass-bottom dish and cultured in complete FDMEM overnight at 37 °C under 5% $CO_2$. Cells were then rinsed with L15 (3 × 1 mL) and incubated with L15 containing Fl-CPD$_{34}$ **1** (60 nM, 0.3 mL). The FLIM images were acquired without exchanging the medium at RT using Leica Stellaris 8 Falcon (63x) with a white laser at 488 nm (20 MHz) and detection at 550-650 nm (Figures 3c and S19).

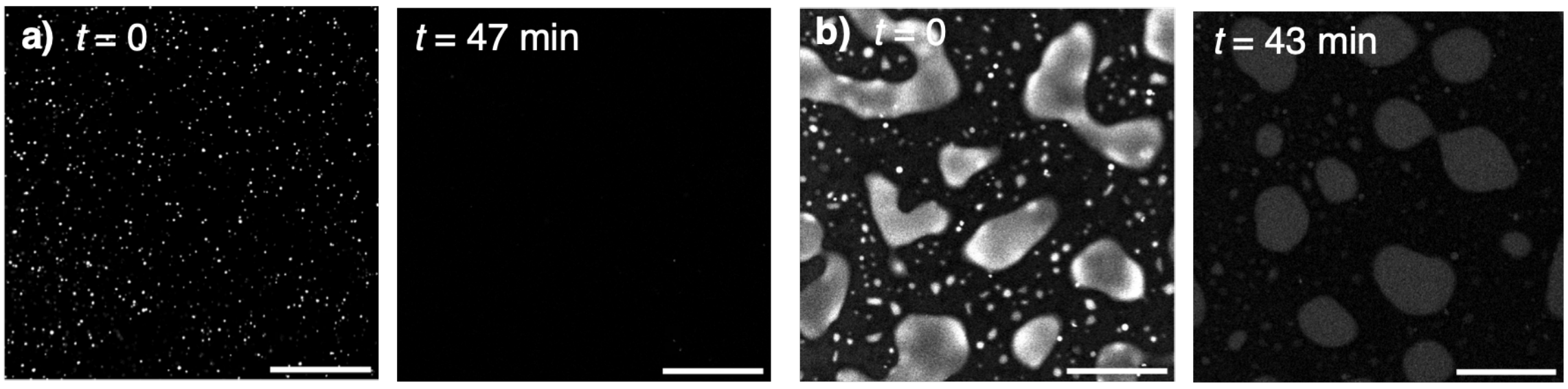


**Figure S18** | FLIM intensity images of Fl-CPD$_{34}$ **1** with (a) polyU or (b) polyR/polyU condensates, before (left) and after addition of GSH, scale bars = 50 μm. Corresponding fast FLIM images are in Figure 3.

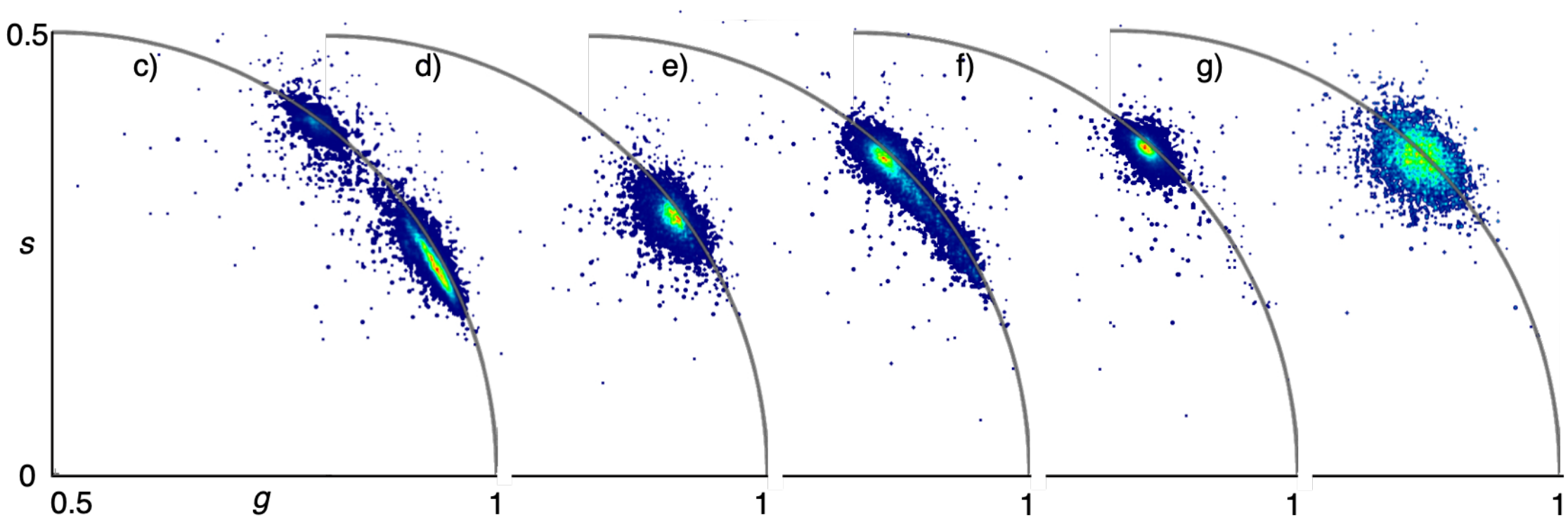


**Figure S19** | Phasor plots corresponding to images in Figure 3.

### 13.2. CPD Depolymerization in Cells

HK cells were seeded at 8.0 × $10^4$ cells/mL on a 35 mm glass-bottom dish and cultured in complete FDMEM overnight at 37 °C under 5% $CO_2$. After removing the medium, cells were rinsed with L15 (3 × 1 mL) and treated with Fl-$CPD_{34}$ **1** (75 nM) in L15. The cells were incubated at 37 °C under 5% $CO_2$ atmosphere for 20 min. Then, the medium was removed by aspiration, and cells were washed with L15 supplemented with 0.1 mg/mL heparin sulfate (3 × 1 mL), L15 (3 × 1 mL) and kept in L15 medium for imaging. The distribution of **1** was analyzed without fixing by CLSM (Leica SP8), using a 63× OI objective lens. Depolymerization of **1** in nucleoli was measured every 10 min on the same cells for at least 1 h. Laser power was fixed at 0.5% to minimize bleaching.

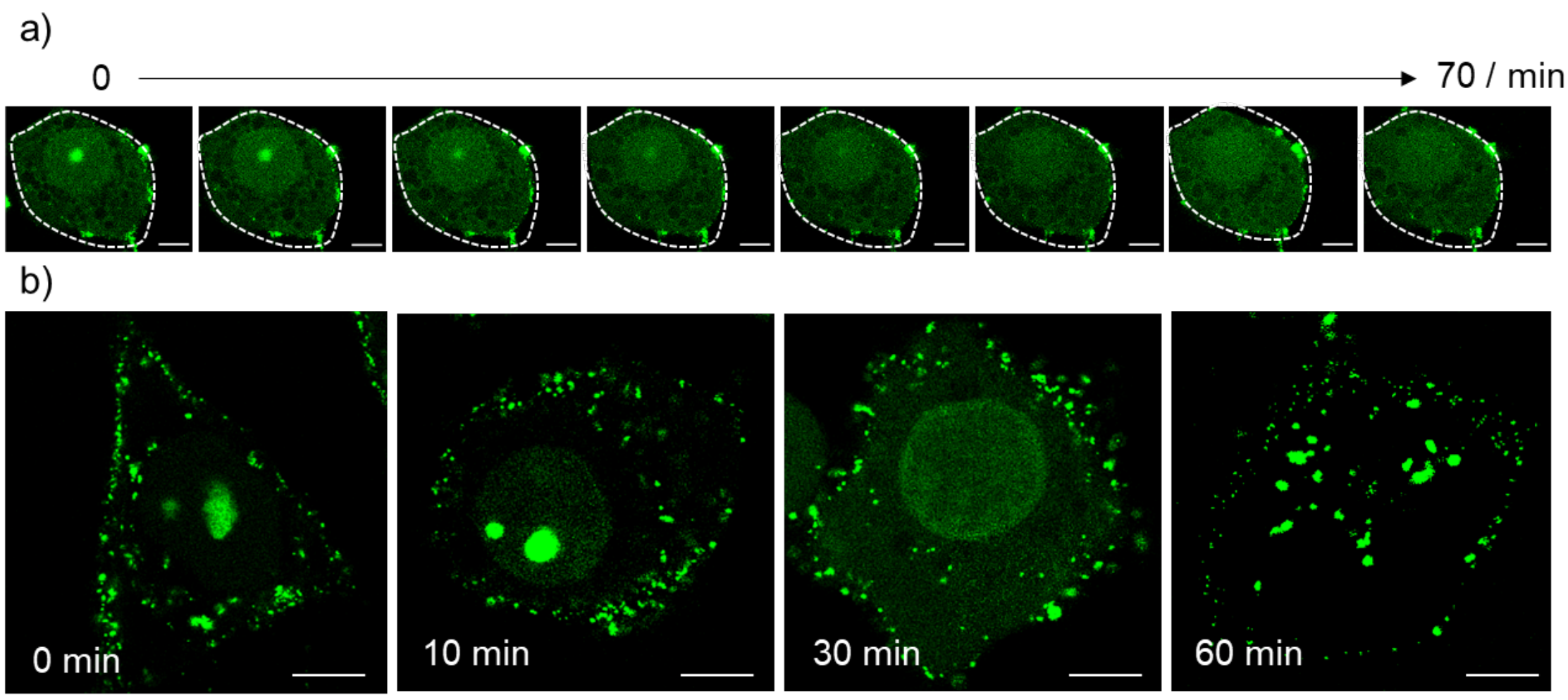


**Figure S20** | Depolymerization of Fl-$CPD_{34}$ **1** (75 nM) in HK cells over time: a) depolymerization in the same cell; b) depolymerization imaged in different cells at fixed incubation time after washing, laser power = 0.5%, scale bars = 5 µm.

## 14. Membrane Reorganization by CPDs

### 14.1. Membrane Reorganization in GUVs

A thin lipid film was prepared by evaporating a preheated (55 °C) solution of POPC/POPG/SM/CL 25:5:40:30 (10 mM) in a mixture of $CHCl_3$ and MeOH (10/1, 20 µL) on the conductive side of a preheated ITO electrode (55 °C) and further drying under vacuum at 55 °C for

2 h. An o-ring covered in silicon grease was placed around the film, and 250 µL of an aqueous sucrose solution (250 mM) was added onto the film. The second ITO electrode was placed on top of the first one, conductive side facing the joint and the sucrose solution. The electrodes were placed in Nanion Vesicle Prep Pro and exposed to an electric field of 1.2 V and 10 Hz for 2 h at 55 °C. The resulting stock solution of GUVs was used without further purification.

Samples were prepared by mixing 30 µL of GUVs, 270 µL of Tris buffer (10 mM Tris, 100 mM NaCl, pH 7.4) and a stock solution of Flipper-TR® **14** (0.15 µL of 0.4 mM in DMSO) in wells of a 8-well slide. The FLIM images were acquired before and after the addition of Bn-CPD$_{38}$ **2** (0.22 µL of 0.14 mM) at RT using Leica Stellaris 8 Falcon (63x) with a white laser at 488 nm (20 MHz) and detection at 550-650 nm.

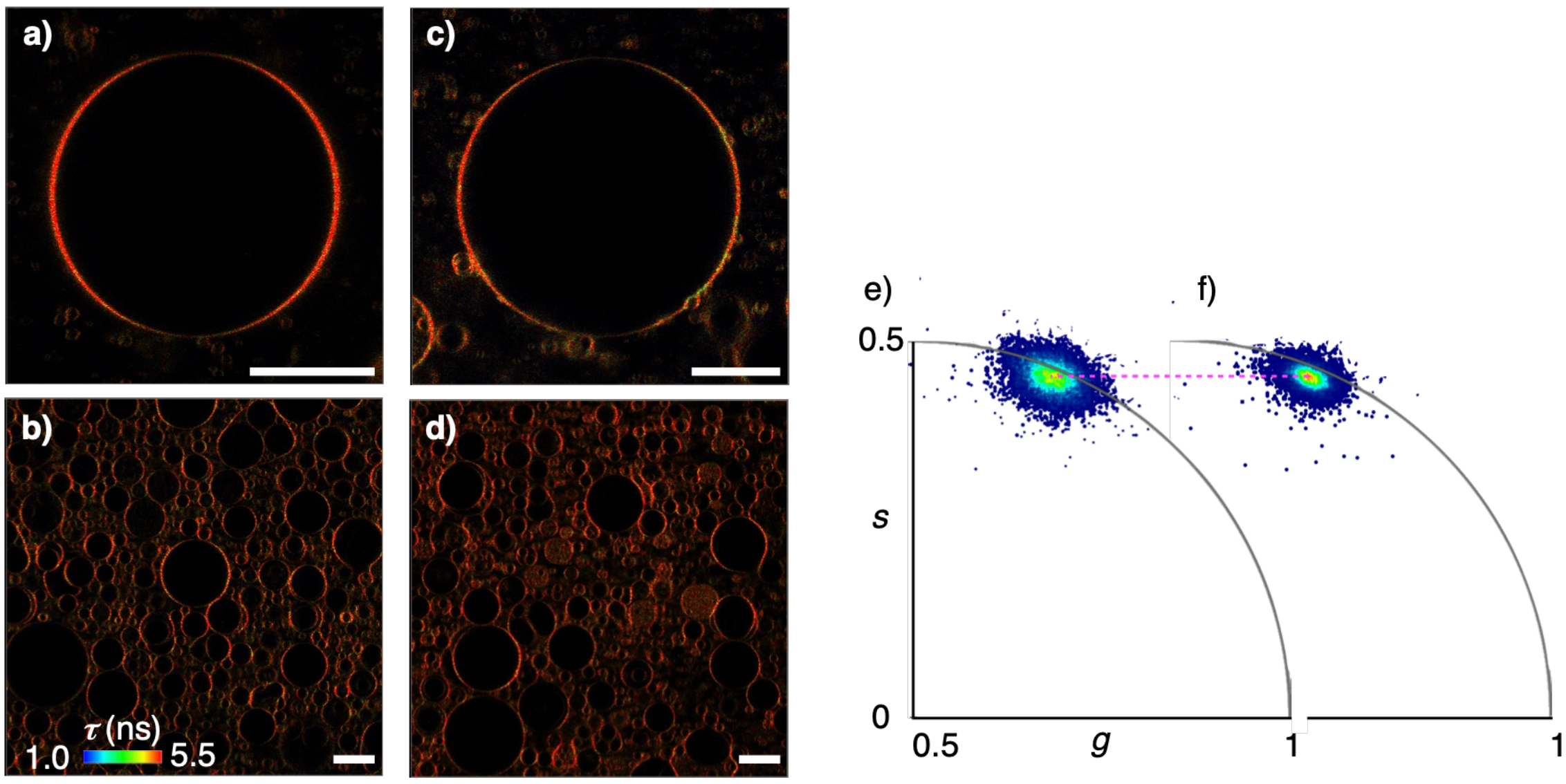


**Figure S21** | FLIM images (a-d) and phasor plots (e, f) of Flipper **14** (0.2 µM) in POPC/POPG/eSM/CL 25:5:40:30 GUVs before (a, b, e; $\tau$ = 5.4 ns) and after the addition of Bn-CPD$_{38}$ **2** (0.1 µM, $c_M$ = 4 µM; c, d, f; $\tau$ = 5.4 ns), scale bars = 20 µm.

### 14.2. Membrane Reorganization in HK Cells

HK cells were plated as described in section 7 at 8.0 × 10$^4$ cells/mL on a 35 mm glass-bottom dish and cultured in complete FDMEM overnight at 37 °C under 5% $CO_2$. Cells were then rinsed with

DPBS (3 × 1 mL) and L15 (2 × 1 mL), and incubated in L15 containing Flipper-TR® **14** (0.2 µM, 1.0 mL). The FLIM images were acquired using Leica Stellaris 8 Falcon (63x) with a white laser at 488 nm (20 MHz) and detection at 550-650 nm during ≈20 min without exchanging the medium at 37 °C under 5% $CO_2$ (Fig. 4a). Then cells were treated with Bn-$CPD_{38}$ **2** (0.1 µM, $c_M$ = 4 µM) or polyR ($c_M$ = 4 µM), and imaged (Figures 4b, c). Alternatively, HK cells after rinsing were incubated with Bn-$CPD_{38}$ **2** (0.1 µM, $c_M$ = 4 µM) for 20 min at 37 °C under 5% $CO_2$, rinsed with 0.1% heparin/L15 (3 × 1 mL) followed by L15 without heparin (2 × 1 mL), and incubated with Flipper-TR® **14** in L15 (0.2 µM, 1.0 mL) for imaging (Figure 4d). For TMU inhibitor treatment, HK cells after rinsing were incubated with dMAC (20 µM) for 1 h at 37 °C under 5% $CO_2$, treated with Flipper-TR® **14** in L15 (0.2 µM, 1.0 mL), imaged during ≈20 min (Figure 4e), treated with Bn-$CPD_{38}$ **2** (0.1 µM, $c_M$ = 4 µM), and imaged (Figure 4f).

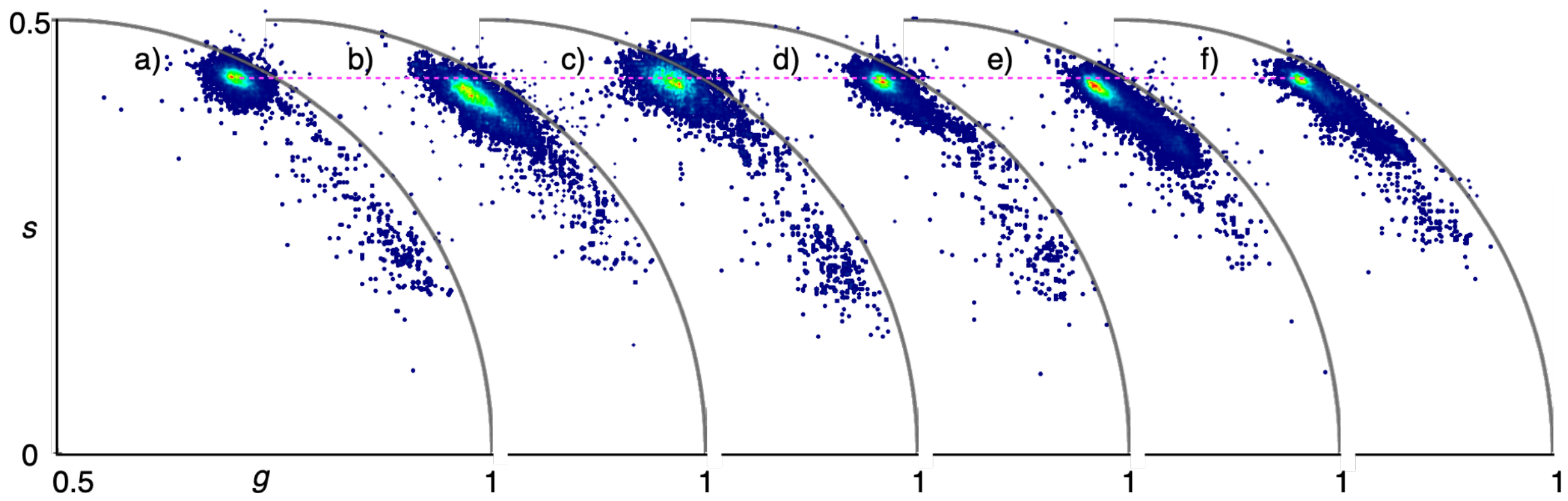


**Figure S22** | Phasor plots corresponding to images in Figure 4.

## 15. Uptake Validation of Proteomics Probes

The HK cells were plated as described in section 7, then medium was removed, and cells were washed with DPBS (3 × 1 mL) followed by fresh L15 (3 × 1 mL). The cells were treated with a solution of **27** (100 mM, DMSO) pre-diluted in L15 to give a solution at 2x final concentration (250 µM), of which 500 µL was added to the cells. After incubating for 15 min under 5% $CO_2$ humidified atmosphere at 37 °C in the dark, a solution of PC-$CPD_{93}$ **25** (115 µM, $H_2O$) was diluted in L15 to give a solution at 2x final concentration (100 nM), of which 500 µL was added on top of

the cells. The cells were incubated under 5% $CO_2$ humidified atmosphere at 37 °C for 20 min in the dark then for additional 10 min at RT while continuously irradiated in the photoreactor at 450 nm. Afterward, the cells were washed with DPBS (3 × 1 mL) and fixed with a solution of 3% PFA (1 mL) for 15 min at RT. The cells were washed with DPBS (3 × 1 mL) and treated with a solution of SAV680 (5 µg/mL, 1mL) in DPBS complemented with 0.05% saponin and 1% BSA for 1 h at RT in the dark. The cells were washed with DPBS (3 × 1 mL) and kept in the same buffer for cell imaging.

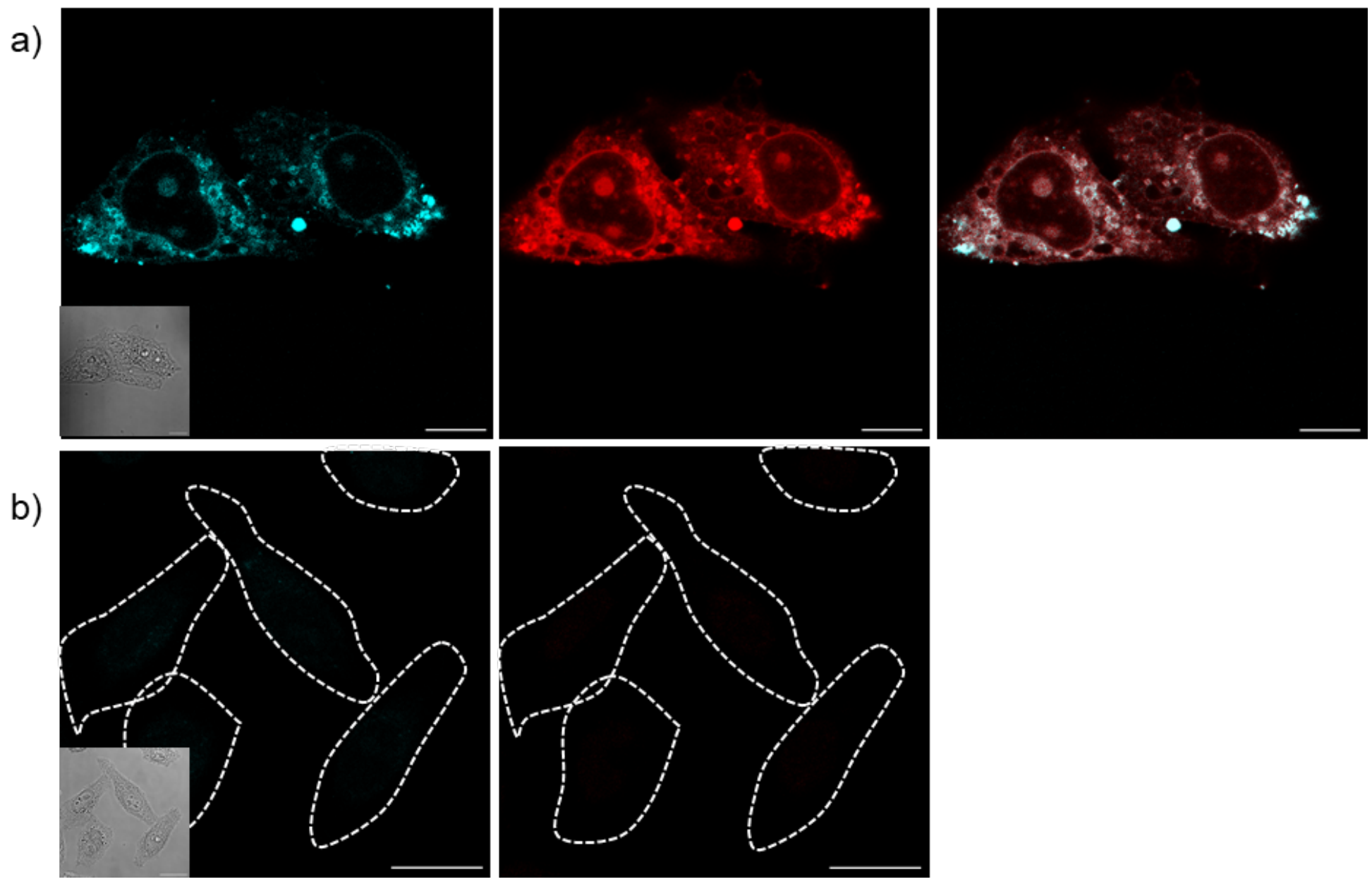


**Figure S23** | CLSM images showing: a) PC-CPD$_{93}$ **25** (100 nM, cyan), SAV680 (5 µg/mL, red), and the corresponding merged image; b) PC-Cys **26** (100 nM, cyan), SAV680 (5 µg/mL, red), and the corresponding merged image. Laser power for PC 40%, laser power for SAV680 = 1%, scale bars = 10 µm.

## 16. µMap Proteomics

### 16.1. General Procedure

The photoreactor used for µMapping is reported in reference [S8]. Cells were seeded ($10^6$ cells/mL, 2 mL) in a 6-well plate (technical triplicate per row) in complete FDMEM and left incubating overnight at 37 °C with 5% $CO_2$. The next day, the cells were washed with DPBS and

covered with 500 µL/well of FDMEM containing 250 µM diazirine **27**. The cells were incubated in the dark for 15 min at 37 °C with 5% $CO_2$. The solution of PC-$CPD_{93}$ **25** (7.8 µL of 115 µM stock in $H_2O$) in FDMEM (500 µL) was added to the cells. Afterward, the cells were exposed to continuous irradiation in a photoreactor (450 nm) for an additional 10 min at RT, with the fan turned on to prevent excessive heating. The cells were washed with DPBS (3 x 1 mL) and subjected to lysis as described in section 8. The protein lysate was adjusted to a final concentration of 1000 µg/mL. Meanwhile, magnetic streptavidin beads (Dynabeads M280, 200 µL of beads for 250 µg of total protein) were transferred to 1.5 mL Lo-Bind tubes and washed once with RIPA 1X (0.5 mL, for 5 min at 800 rpm in a plateshaker). The beads were pelleted using a magnetic rack, then resuspended in 250 µL of the protein samples per tube. The mixture was incubated in a thermoshaker at 4 °C overnight (at least 12 h) with shaking at 800 rpm. The beads were pelleted on a magnetic rack, and the supernatant was discarded. The beads were subsequently washed for 5 min at 4 °C each time with 1% SDS in DPBS (3 x 1 mL/tube), 1 M NaCl in DPBS (3 x 1 mL/tube), 10% EtOH in DPBS (3 x 1 mL/tube), DPBS (6 x 1 mL/tube) and finally 100 mM $NH_4HCO_3$ solution (1 mL/tube). 20 µL were taken for an additional western blot analysis as described in section 9 to confirm the labeling efficiency and the presence of proteins on the beads.

Pull-down samples were treated using iST kits (Preomics) according to manufacturer's instruction. Briefly, samples were re-suspended in 50 µL of provided lysis buffer and proteins were denatured, reduced and alkylated during 10 min at 60 °C. The resulting slurries (beads and lysis buffer) were transferred to dedicated cartridges and proteins were digested with a Trypsin/LysC mix for 2 h at 37 °C. After two cartridge washes, peptides were eluted with 2 x 100 µL of provided elution buffer. Samples were finally completely dried under speed vacuum and stored at -20°C.

### 16.2. Data Acquisition

Samples were dissolved in 20 µL of loading buffer (5% $CH_3CN$, 0.1% FA) and 4 µL were injected on column. LC-ESI-MS/MS was performed on an Orbitrap Fusion Lumos Tribrid mass

spectrometer (Thermo Fisher Scientific) equipped with a Vanquish NEO liquid chromatography system (Thermo Fisher Scientific). Peptides were trapped on an Acclaim pepmap100, $C_{18}$, 3 µm, 75 µm x 20 mm nano trap-column (Thermo Fisher Scientific) and separated on a 75 µm x 500 mm, $C_{18}$ ReproSil-Pur (Dr. Maisch GmBH), 1.9 µm, 100 Å, home-made column. The analytical separation was run for 85 min using a gradient of $H_2O$/FA 99.9%/0.1% (solvent A) and $CH_3CN$/FA 80%/0.1% (solvent B). The gradient was run from 95% A and 5% B to 65% A and 35% B in 60 min, and then to 1% A and 99% B in 10 min with a stay for 15 min at this composition. Flow rate was of 250 nL/min. Data-dependant analysis (DDA) was performed with MS1 full scan at a resolution of 120'000 FWHM followed by as many subsequent MS2 scans on selected precursors as possible within 3 second maximum cycle time. MS1 was performed in the Orbitrap with an AGC target of 4 x $10^5$, a maximum injection time of 50 ms and a scan range from 400 to 2000 m/z. MS2 was performed in the ion-trap with an AGC target at 1 x $10^4$ and a maximum injection time of 35 ms. Isolation windows was set at 1.2 m/z and 30% normalized collision energy was used for higher-energy collisional dissociation (HCD). Dynamic exclusion was set to 20s.

### 16.3. Data Analysis

Raw data were processed using Proteome Discoverer 2.4 software (Thermo Fisher Scientific). Briefly, spectra were extracted and searched against the human reference proteome database (uniprot, 20'419 entries), and an in-house database of common contaminants using Mascot (Matrix Science, London, UK; version 3.1.0). Trypsin was selected as the enzyme, with one potential missed cleavage. Precursor ion tolerance was set to 10 ppm and fragment ion tolerance to 0.6 Da. Carbamidomethylation of cysteine was specified as fixed modification. Variable amino acid modifications were oxidized methionine. Peptide-spectrum matches were validated using Percolator a target FDR of 0.01 and a Delta Cn of 0.5. For label-free quantification, features and chromatographic peaks were detected using the "Minora Feature Detector" Node with the default parameters. PSM and peptides were filtered with a false discovery rate (FDR) of 1%, and then

grouped to proteins with again an FDR of 1% (strict) or 5% (relaxed) and using peptides with high confidence level. Low abundance resampling was used for missing value imputation. Both unique and razor peptides were used for quantitation, and protein abundances are calculated as the average of the three most abundant distinct peptide group. The abundances were normalized on the "Total Peptide Amount" and then "Protein abundance based" option was selected for protein ratio calculation and associated p-values were calculated with an ANOVA test (individual proteins).

UniProt ID mapping was used to determine subcellular localization of significantly enriched proteins.

## 17. Protein Knockdown

### 17.1. Level of Expression

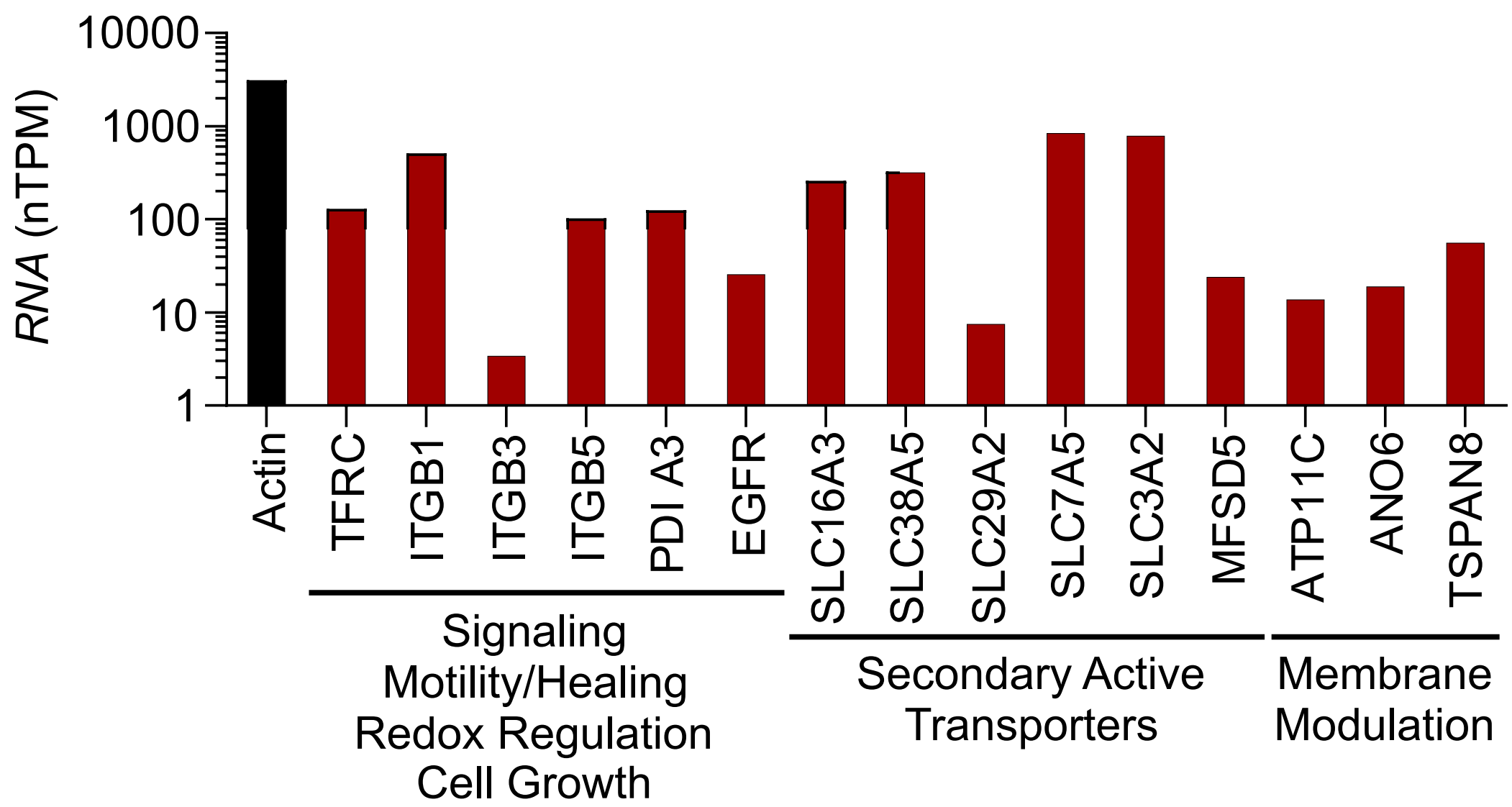


**Figure S24** | The level of expression of the hit proteins in selected cell lines. These data were obtained from https://www.proteinatlas.org.

### 17.2. STRING Analysis

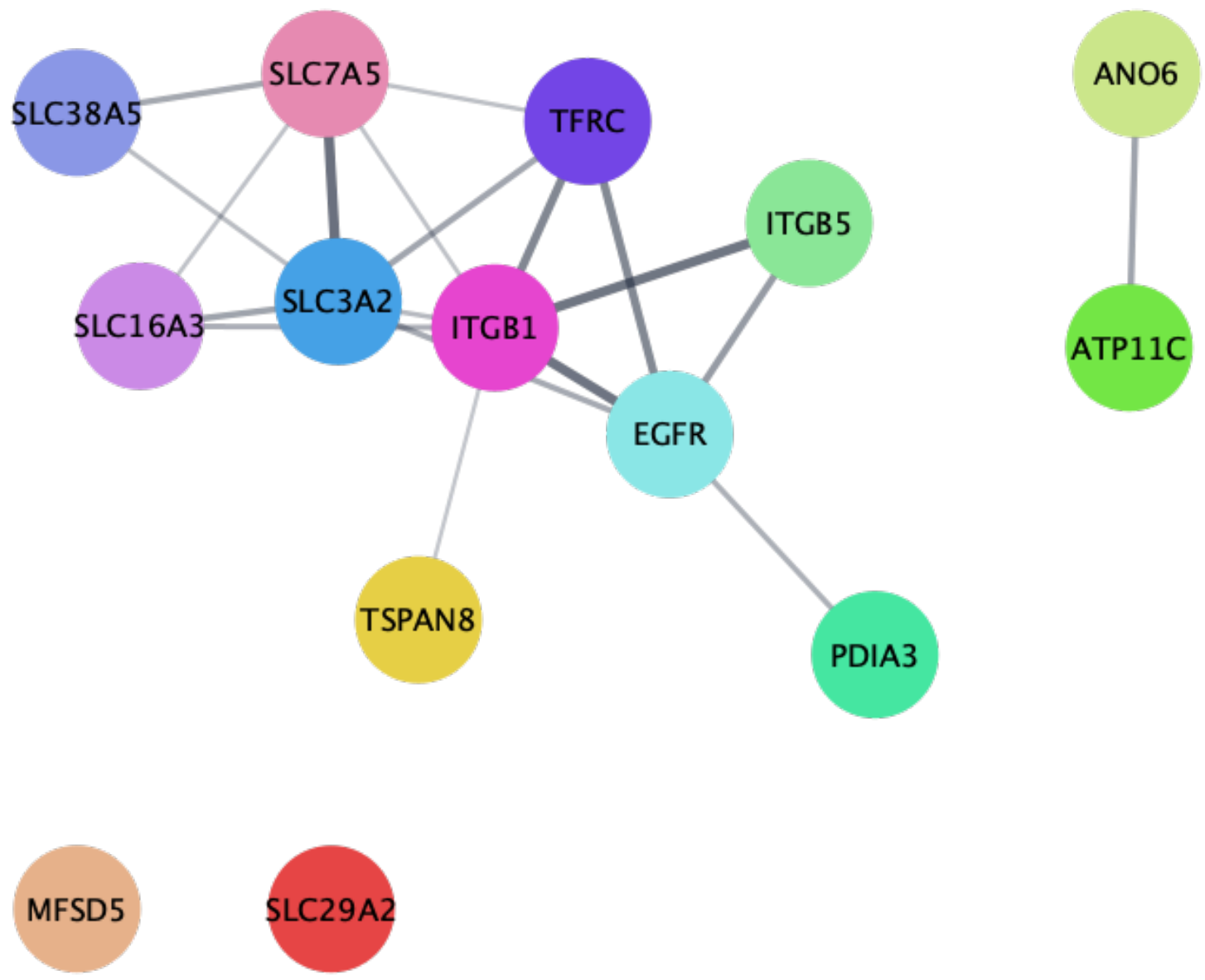


**Figure S25** | The STRING network of proteins of interest from https://string-db.org/, visualized using Cytoscape (ver. 3.10.4, clustered). Thicker and darker lines between protein nodes indicate higher "scores", i.e., confidence.

### 17.3. Knockdown Procedure

To knockdown selected proteins, reverse transfection was performed with a final siRNA concentration of 5 nM, following the transfection procedure provided by siTOOLs Biotech. Briefly, siRNA was diluted in reduced serum Opti-MEM medium and pre-mixed with Lipofectamine RNAiMAX in Opti-MEM for 15 min at RT. The siRNA mixture was then added to a 6-well sterile plate (500 µL per well). HK cells were seeded at $2 \times 10^5$ cells per well and incubated at 37 °C with 5% $CO_2$ for 48 h. After incubation, cells were detached using 500 µL of TrypLE Express at 37 °C for 5 min, followed by the addition of 1 mL of complete FDMEM (supplemented with FCS and PS). The cells were spun down at 1000 rpm for 5 min, resuspended in complete FDMEM, and seeded at $12 \times 10^3$ cells on a 35 mm glass-bottom dish. The dish was then incubated overnight at 37 °C with 5% $CO_2$. Knockdown efficiency was validated either by immunofluorescence, western blot or provided by the supplier.

### 17.4. Cellular Uptake in Knocked-Down Cells

Following the general procedure for cellular uptake described in section 10.1. with Fl-CPD **1** or GFP-CPD **7**. The fluorescence distribution was quantified using either the 63× OI or 20× WI objective lens covering 50 – 100 cells per image, with at least independent duplicates per protein knockdown.

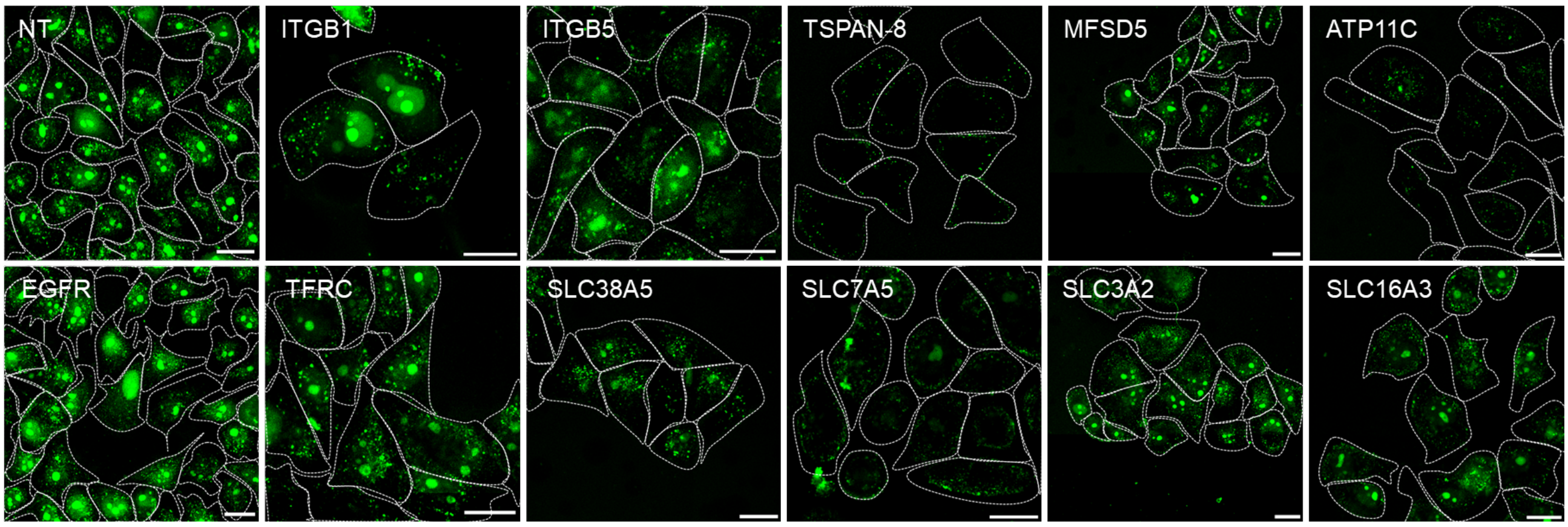


**Figure S26** | CLSM images showing fluorescence intensity of **1** (75 nM, green) in live KD cells after incubation for 20 min in L15, laser power = 2%, scale bars = 20 µm, NT = non-target.

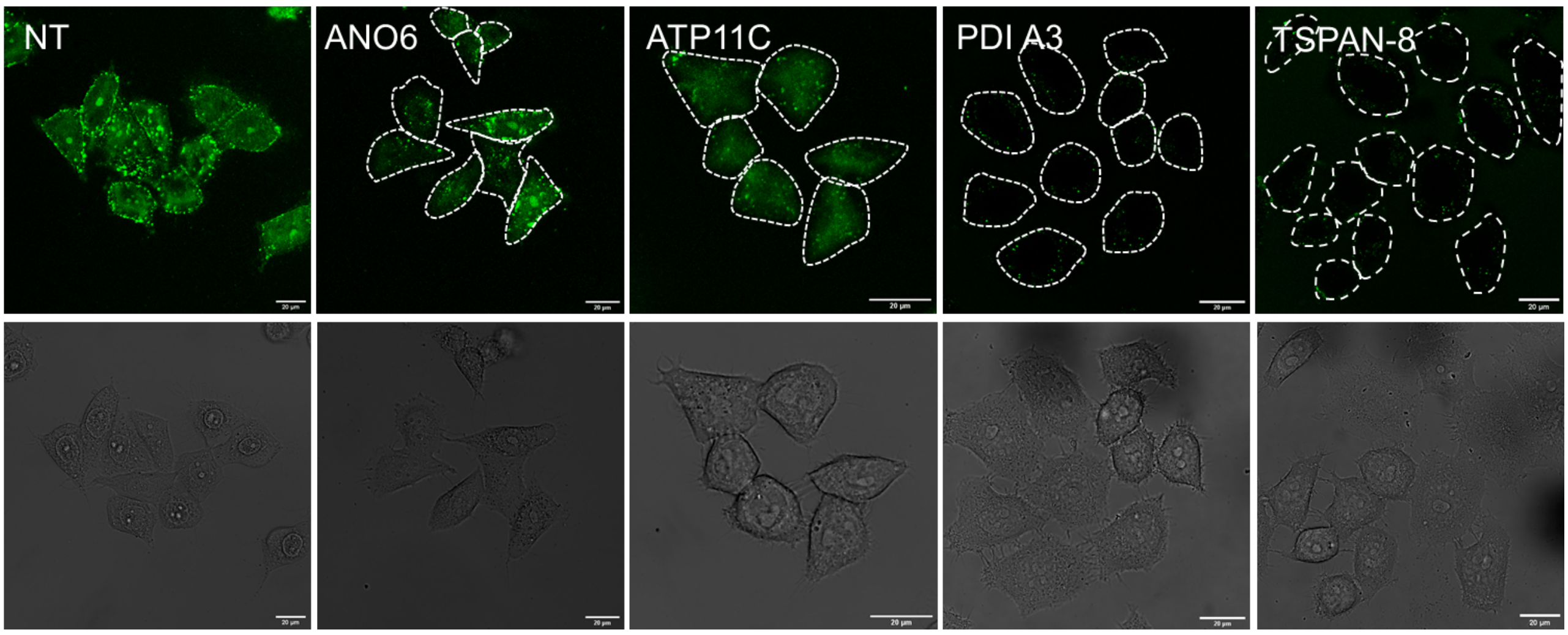


**Figure S27** | CLSM images showing fluorescence intensity of **7** (75 nM, green) in live KD cells after incubation for 30 min in L15, laser power = 10%, scale bars = 20 µm, NT = non-target.

## 18. Calcium Dependence

*BAPTA-AM assay.* The HK cells were plated as described in section 7 at 8.0 × $10^4$ cells/mL on a 35 mm glass-bottom dish and cultured in complete FDMEM overnight at 37 °C under 5% $CO_2$. Cells were then rinsed with DPBS (3 × 1 mL) and L15 (3 × 1 mL), and incubated with L15 containing BAPTA-AM (40 µM, 1.0 mL) for 0.5 h at 37 °C under 5% $CO_2$. Then cells were rinsed with L15 (3 × 1 mL), incubated with L15 containing Fl-$CPD_{55}$ **1** (63 nM, 1.0 mL) for 15 min at 37 °C under 5% $CO_2$, heparin washed (3 × 1 mL 0.1% heparin/L15, 2 × 1 mL L15), and imaged by CLSM ($l_{ex}$ = 488 nm, detection 500-550 nm) using 63× oil immersion lens at RT.

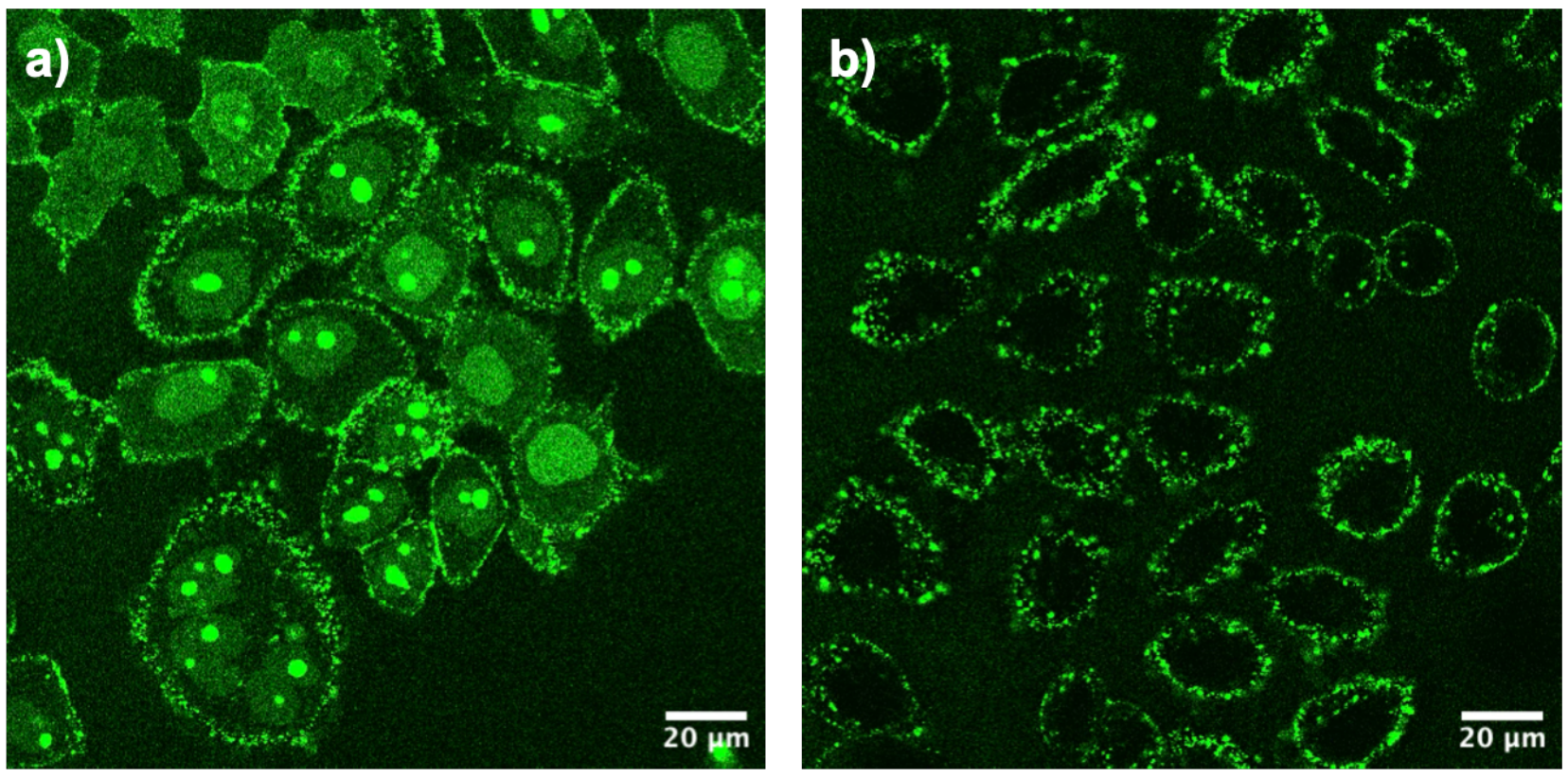


**Figure S28** | Effect of intracellular $Ca^{2+}$ on the uptake of Fl-CPDs **1** (63 nM) in HK cells (a) without or with (b) BAPTA-AM (40 µM) pretreatment imaged by CLSM. Brightness and contrast were set equal, scale bars = 20 µm.

*EDTA assay.* The HK cells were plated as described in section 7 at 8.0 × $10^4$ cells/mL on a 35 mm glass-bottom dish and cultured in complete FDMEM overnight at 37 °C under 5% $CO_2$. Cells were then rinsed with DPBS (3 × 1 mL) and L15 (3 × 1 mL), and incubated with Fl-$CPD_{34}$ **1** (75 nM, 1 mL) in either L15 or L15 supplemented with EDTA (5 mM) for 15 min at 37 °C under 5% $CO_2$. Then cells were rinsed with L15 (3 × 1 mL), heparin washed (3 × 1 mL 0.1% heparin/L15, 2 × 1 mL L15), and kept for imaging in L15.

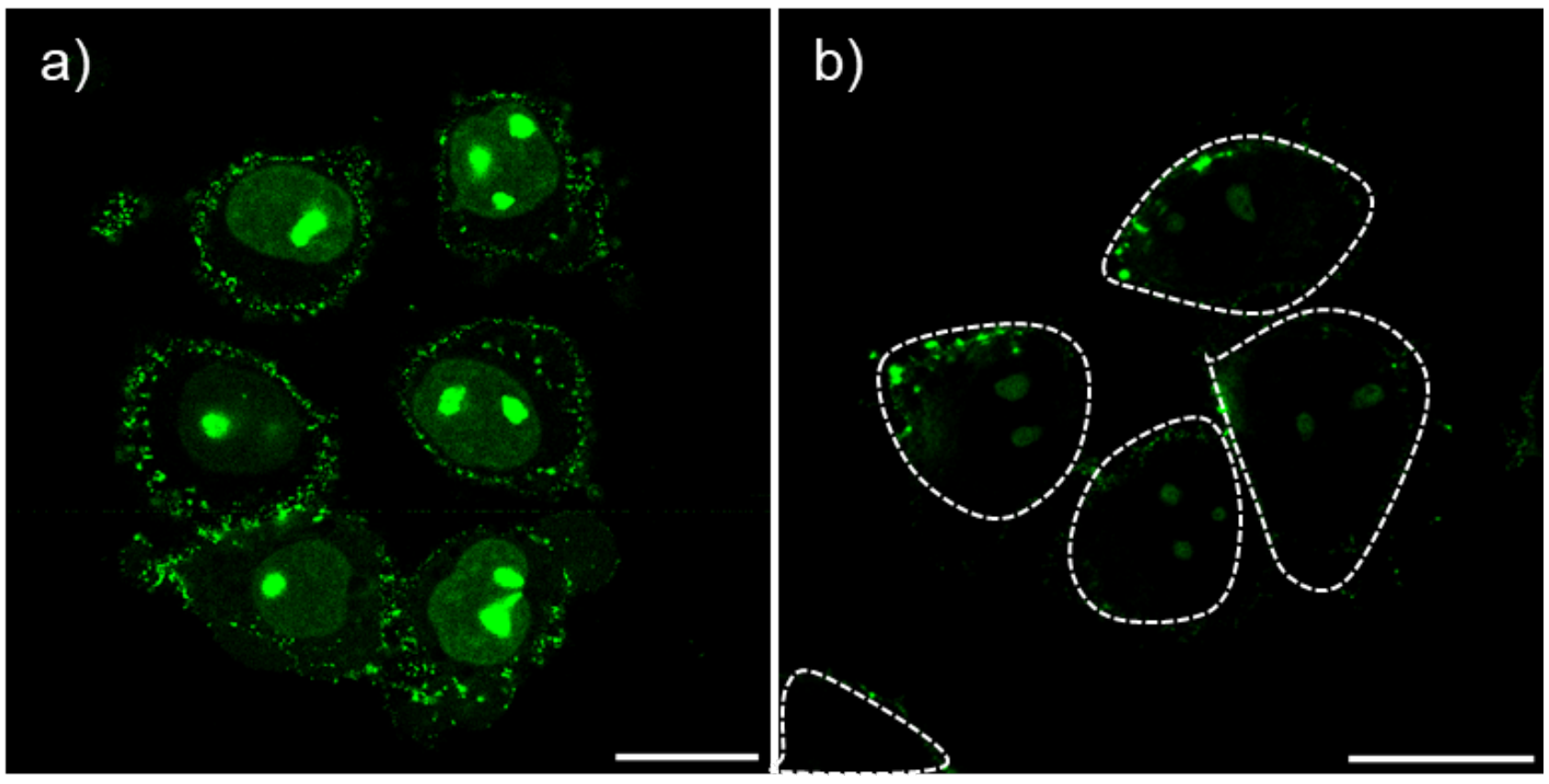


**Figure S29** | Effect of intracellular $Ca^{2+}$ on the uptake of Fl-CPDs **1** (75 nM) in HK cells (a) without or with (b) EDTA (5 mM) pretreatment imaged by CLSM. Brightness and contrast were set equal, scale bars = 20 µm.

## 19. Supporting References

S1. S. Saidjalolov, F. Coelho, V. Mercier, D. Moreau, and S. Matile, "Inclusive Pattern Generation Protocols to Decode Thiol-Mediated Uptake," *ACS Central Science 10* (2024): 1033–1043, https://doi.org/10.1021/acscentsci.3c01601.

S2. C. Huang, Y.-C. Liu, H. Oh, D.-S. Guo, W. M. Nau, A. Hennig, 'Cellular Uptake of Cell-Penetrating Peptides Activated by Amphiphilic p-Sulfonatocalix[4]arenes', *Chemistry – A European Journal 30* (2024): e202400174, https://doi.org/10.1002/chem.202400174.

S3. G. Gasparini, G. Sargsyan, E.-K. Bang, N. Sakai, and S. Matile, "Ring Tension Applied to Thiol-Mediated Cellular Uptake," *Angewandte Chemie International Edition 54* (2015): 7328–7331, https://doi.org/10.1002/anie.201502358.

S4. L. Zong, E. Bartolami, D. Abegg, A. Adibekian, N. Sakai, and S. Matile, "Epidithiodiketopiperazines: Strain-Promoted Thiol-Mediated Cellular Uptake at the Highest Tension," *ACS Central Science 3* (2017): 449–453, https://doi.org/10.1021/acscentsci.7b00080.S5.

S5. I. Shybeka, J. R. J. Maynard, S. Saidjalolov, D. Moreau, N. Sakai, and S. Matile, "Dynamic Covalent Michael Acceptors to Penetrate Cells: Thiol-Mediated Uptake with Tetrel-Centered Exchange Cascades, Assisted by Halogen-Bonding Switches," *Angewandte Chemie International*

*Edition 61* (2022): e202213433, https://doi.org/10.1002/anie.202213433.

S6. Y. Cheng, L. Zong, J. López-Andarias, E. Bartolami, Y. Okamoto, T. R. Ward, N. Sakai, S. Matile, 'Cell-Penetrating Dynamic-Covalent Benzopolysulfane Networks', *Angewandte Chemie International Edition 58* (2019): 9522–9526, https://doi.org/10.1002/anie.201905003.

S7. M. Cognet, G. Renno, F. Coelho, N. Sakai, S. Matile, 'Grafting Cell-Penetrating Poly(disulfide)s to Substrates of Interest: Dynamic Covalent Bioconjugation for Traceless Delivery', *Angewandte Chemie International Edition 65* (2026): e17229, https://doi.org/10.1002/anie.202517229.

S8. S. Saidjalolov, Y. Wu, G. Renno, N. Rose, J. Gajić, B. Pologne, N. Winssinger, V. Mercier, D. Moreau, N. Sakai, S. Matile, 'Photocatalytic Microenvironment Proteomics of Thiol-Mediated Uptake', *JACS Au 5* (2025): 3288–3298, https://doi.org/10.1021/jacsau.5c00432.

S9. G. Gasparini, E.-K. Bang, G. Molinard, D. V. Tulumello, S. Ward, S. O. Kelley, A. Roux, N. Sakai, S. Matile, 'Cellular uptake of substrate-initiated cell-penetrating poly(disulfide)s', *Journal of the American Chemical Society. 136* (2014):6069–6074, http://doi.org/10.1021/ja501581b.

S10 E.-K. Bang, G. Gasparini, G. Molinard, A. Roux, N. Sakai, S. Matile, 'Substrate-Initiated Synthesis of Cell-Penetrating Poly(disulfide)s', *Journal of the American Chemical Society. 135* (2013): 2088–2091, https://doi.org/10.1021/ja311961k.

S11. S. Saidjalolov, X.-X. Chen, J. Moreno, M. Cognet, L. Wong-Dilworth, F. Bottanelli, N. Sakai, and S. Matile, "Asparagusic Golgi Trackers," *JACS Au 4* (2024): 3759–3765, https://doi.org/10.1021/jacsau.4c00487.

S12. N. Klonis, A. H. A. Clayton, E. W. Voss Jr., W. H. Sawyer, 'Spectral Properties of Fluorescein in Solvent-Water Mixtures: Applications as a Probe of Hydrogen Bonding Environments in Biological Systems', *Photochemistry & Photobiology 67* (1998): 500–510, https://doi.org/10.1111/j.1751-1097.1998.tb09085.

## 20. NMR Spectra

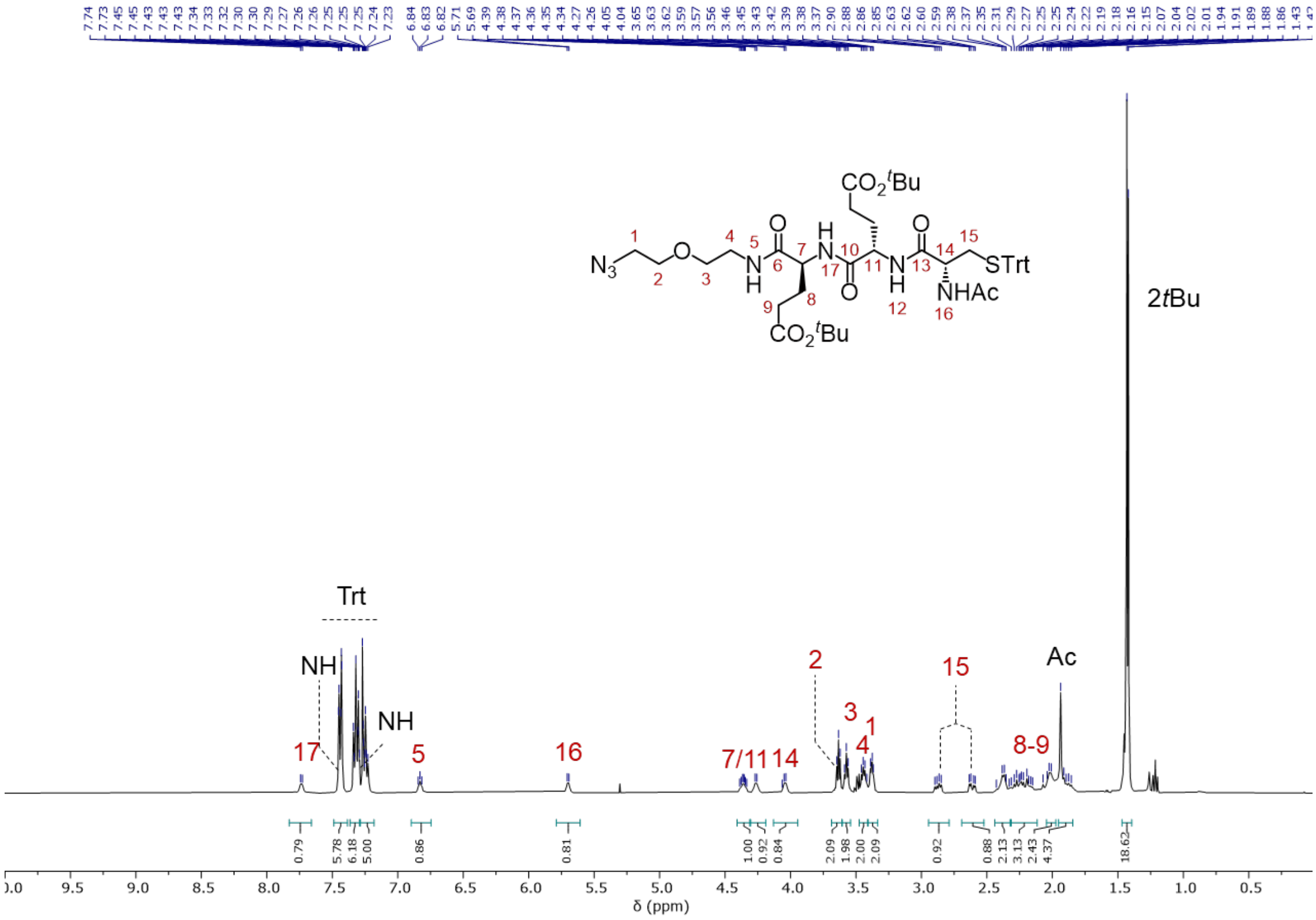


**Figure S30** | $^{1}$H NMR (400 MHz, $CDCl_3$) spectrum of compound **43**.

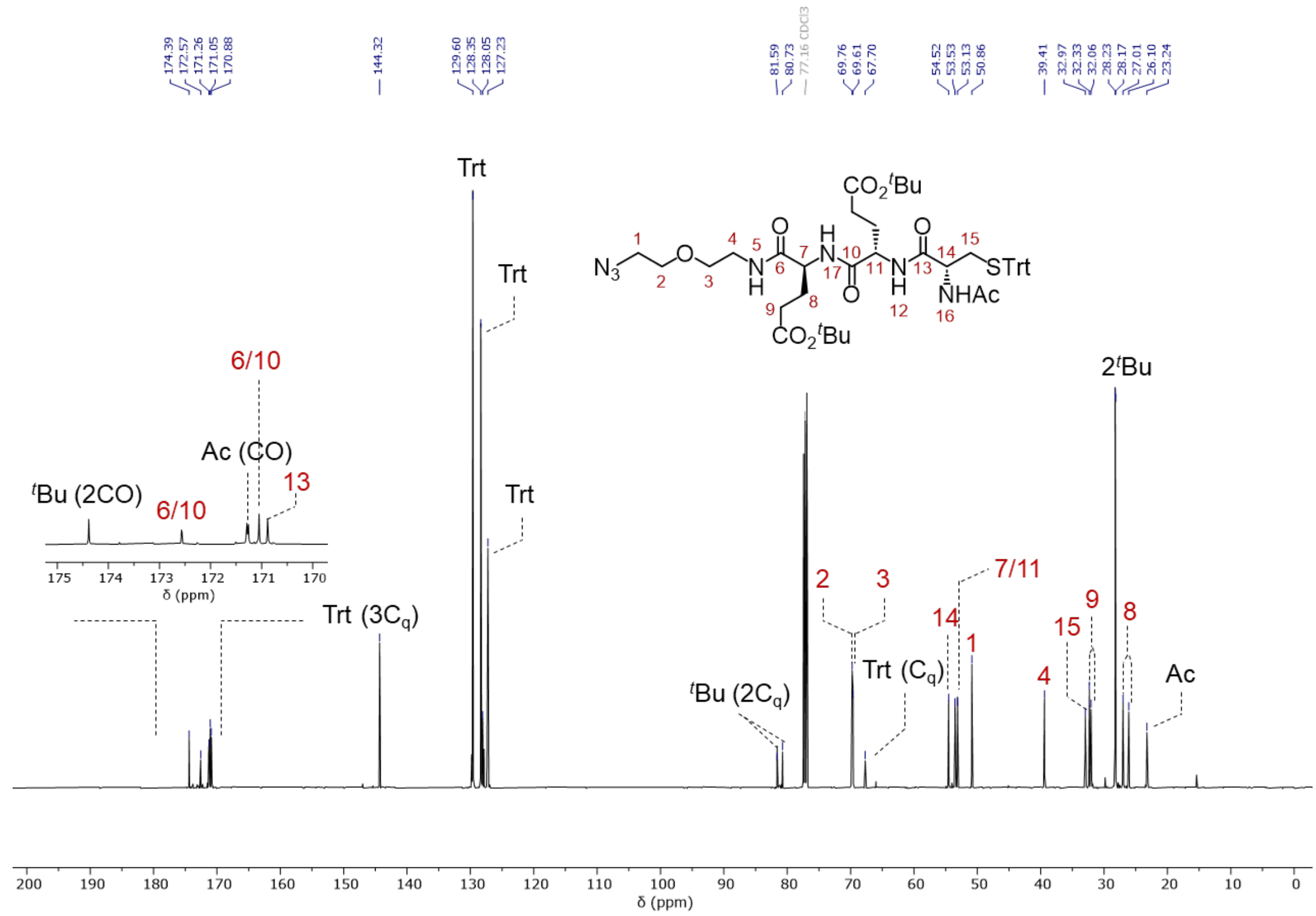

**Figure S31** | $^{13}$C NMR (126 MHz, $CDCl_3$) spectrum of compound **43**.

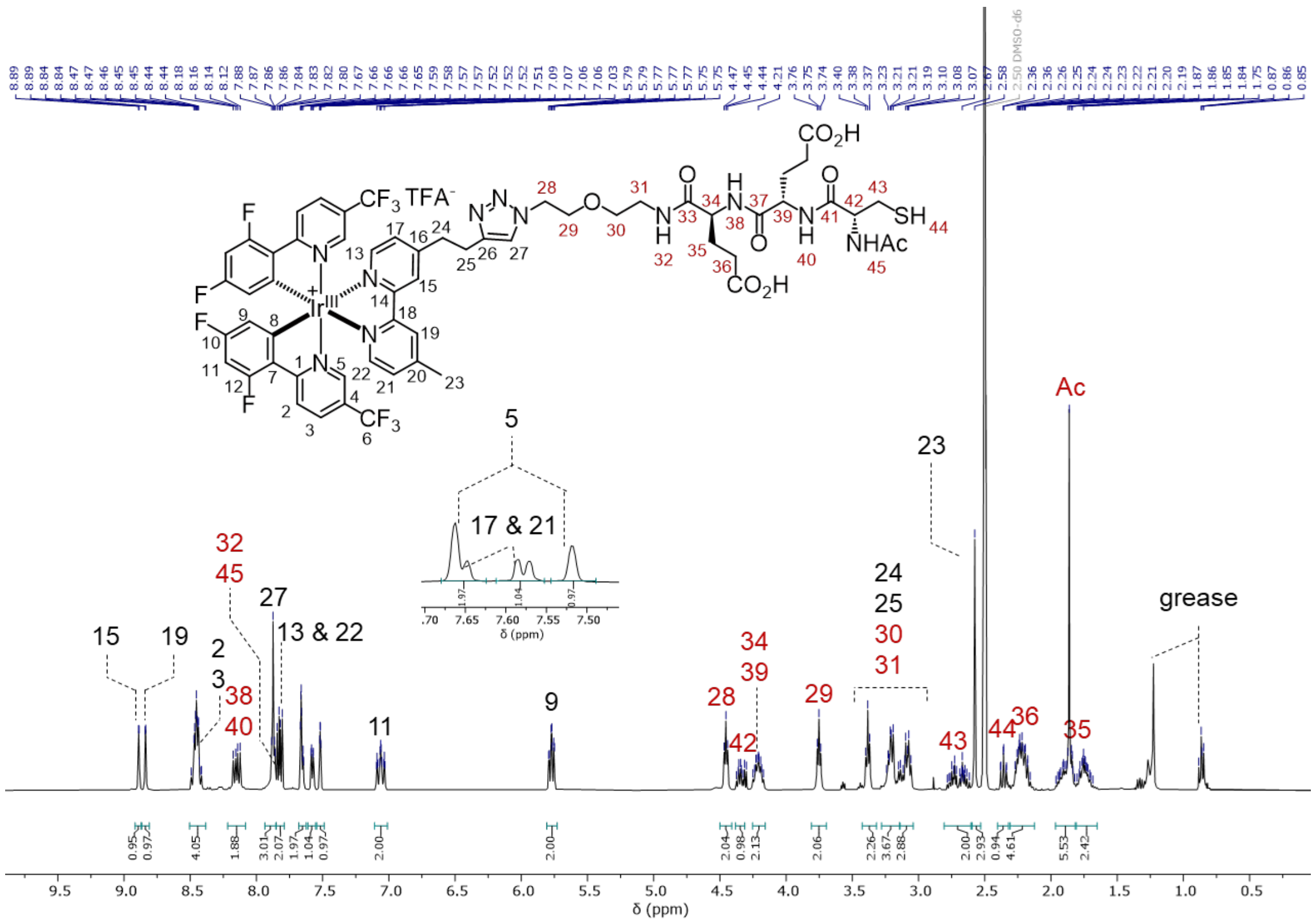

**Figure S32** | $^{1}$H NMR (400 MHz, DMSO-$d_6$) spectrum of compound **26** TFA salt.

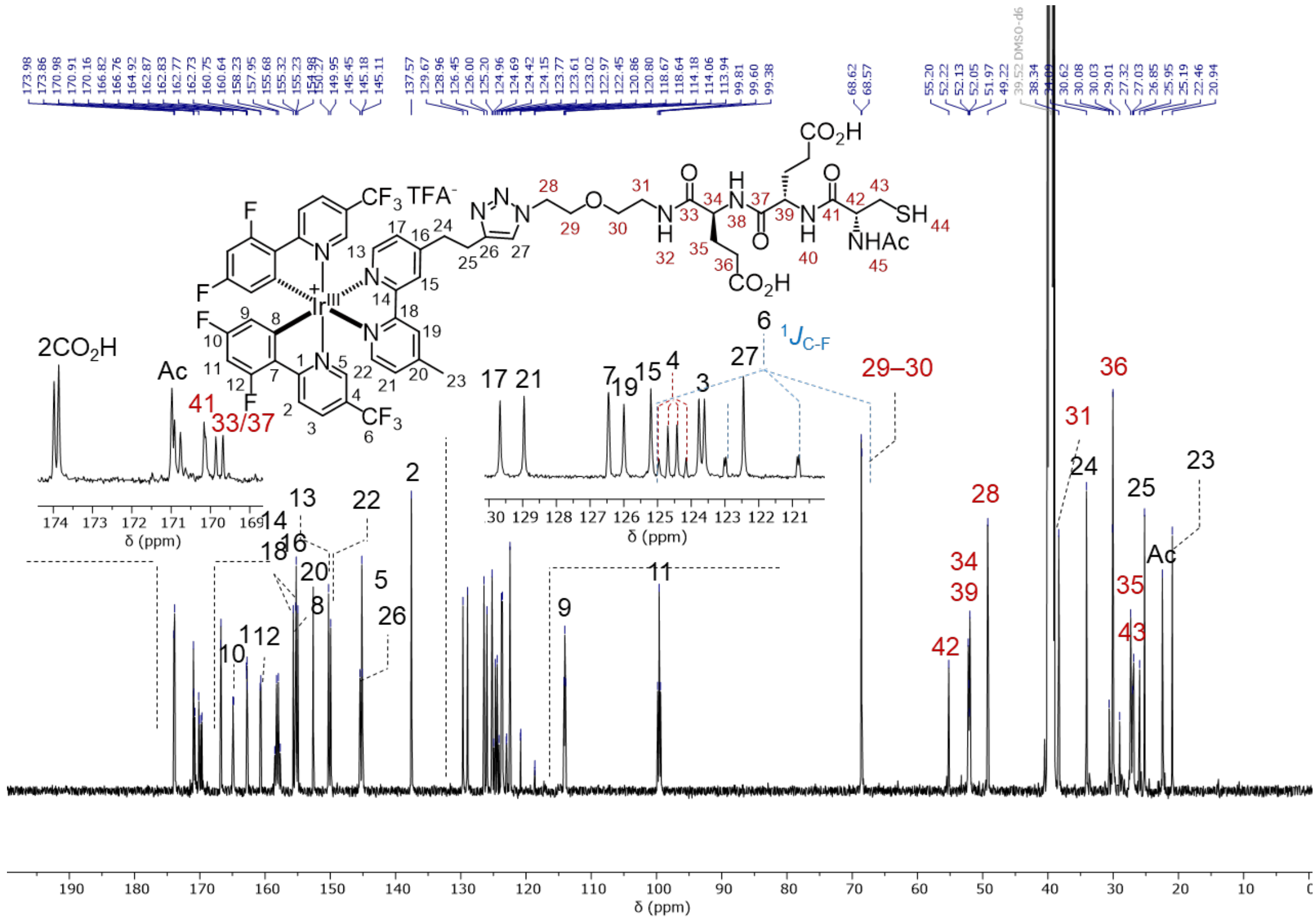

**Figure S33** | $^{13}$C NMR (126 MHz, DMSO-$d_6$) spectrum of compound **26** TFA salt.

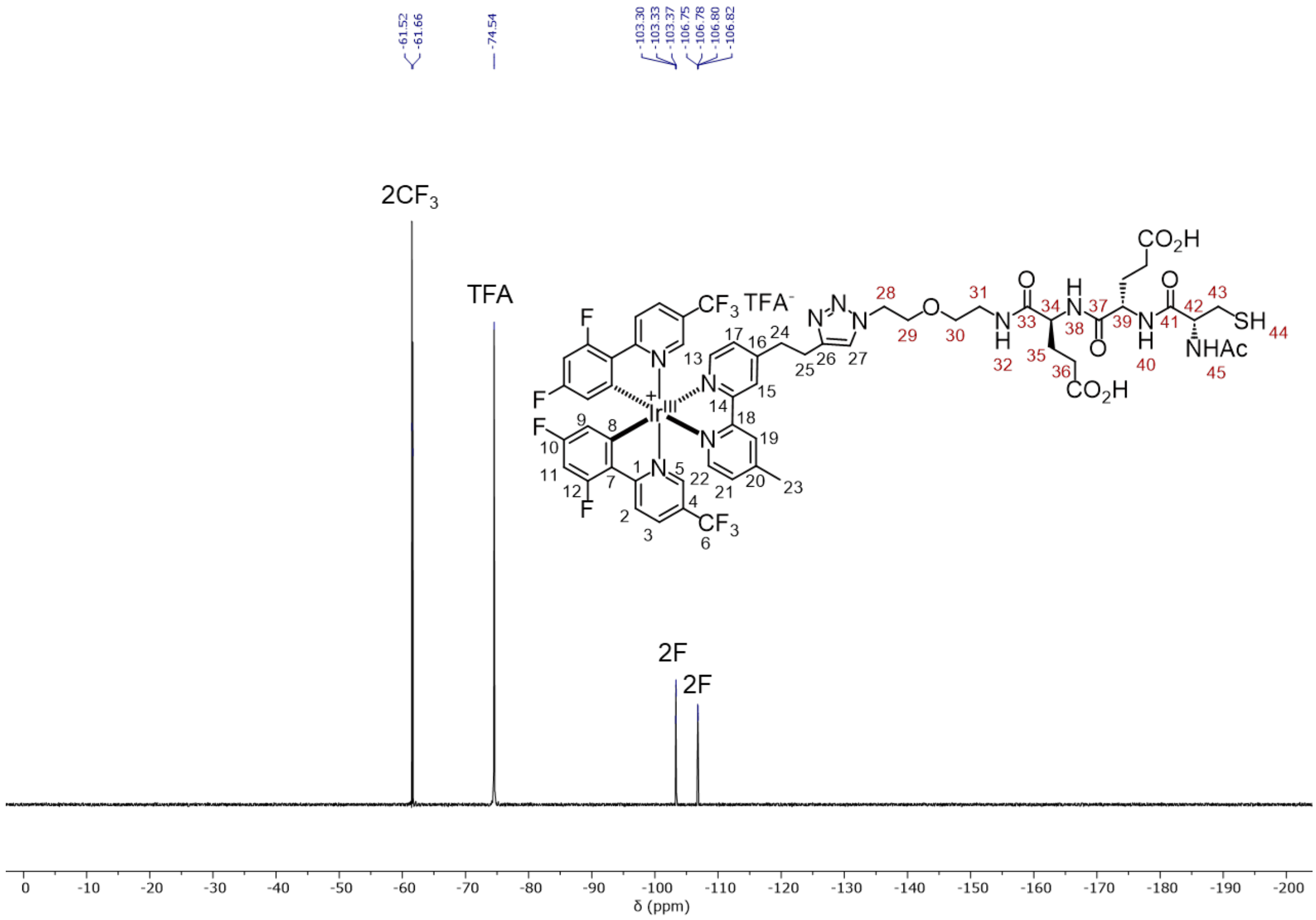

**Figure S34** | $^{19}$F NMR (282 MHz, DMSO-$d_6$) spectrum of compound **26** TFA salt.
